# Spin‐Labeled Riboswitch Synthesized from a Protected TPA Phosphoramidite Building Block

**DOI:** 10.1002/chem.202201822

**Published:** 2022-08-18

**Authors:** Frank Kaiser, Burkhard Endeward, Alberto Collauto, Ute Scheffer, Thomas F. Prisner, Michael W. Göbel

**Affiliations:** ^1^ Institute for Organic Chemistry and Chemical Biology Goethe University Frankfurt Max-von-Laue-Strasse 7 60438 Frankfurt am Main Germany; ^2^ Institute for Physical and Theoretical Chemistry Goethe University Frankfurt Max-von-Laue-Strasse 7 60438 Frankfurt am Main Germany

**Keywords:** nitroxide, PELDOR, photocaged spin label, RNA aptamer, secondary structure mapping

## Abstract

The nitroxide TPA (2,2,5,5‐tetramethyl‐pyrrolin‐1‐oxyl‐3‐acetylene) is an excellent spin label for EPR studies of RNA. Previous synthetic methods, however, are complicated and require special equipment. Herein, we describe a uridine derived phosphoramidite with a photocaged TPA unit attached. The light sensitive 2‐nitrobenzyloxymethyl group can be removed in high yield by short irradiation at 365 nm. Based on this approach, a doubly spin‐labeled 27mer neomycin sensing riboswitch was synthesized and studied by PELDOR. The overall thermal stability of the fold is not much reduced by TPA. In‐line probing nevertheless detected changes in local mobility.

## Graphical abstract

**The nitroxide TPA** (2,2,5,5‐tetramethyl‐pyrrolin‐1‐oxyl‐3‐acetylene) has been protected with a light sensitive 2‐NBOM group and connected to a uridine phosphoramidite building block. Spin‐labeled RNA can be obtained from this compound by standard protocols of oligonucleotide synthesis and used for EPR studies after light‐induced deprotection.

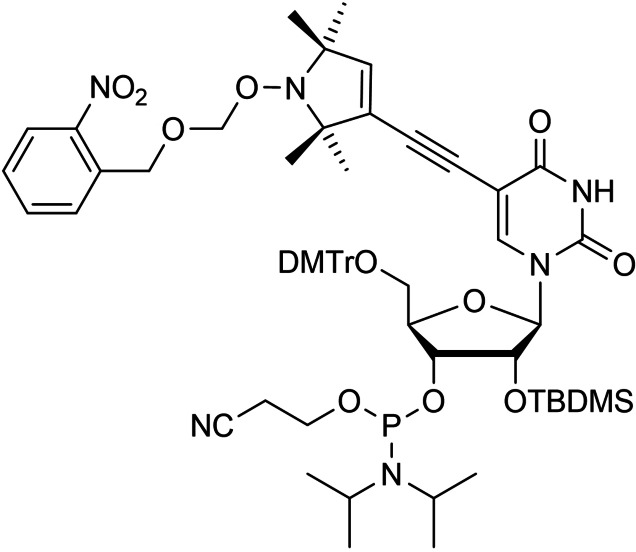

## Introduction

**Figure 1:**
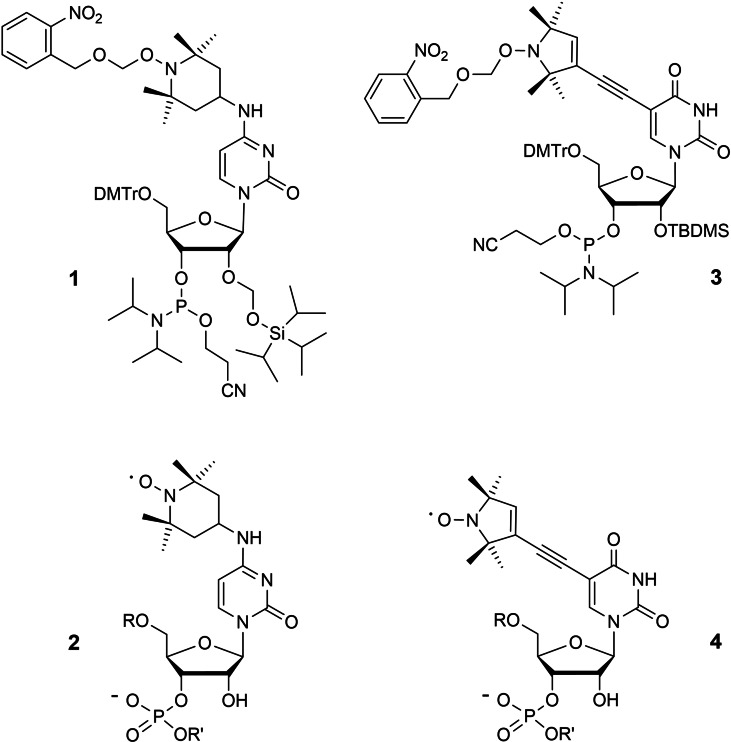
Phosphoramidite building blocks containing protected TEMPO (**1**) and TPA (**3**) spin labels. The resulting final RNA structures **2** and **4** are shown below.

Nitroxide radicals[^1^, ^2^, ^3^] are among the most useful spin labels to study the structure and dynamics of RNA by EPR spectroscopy.[^4^, ^5^, ^6^, ^7^, ^8^, ^9^, ^10^, ^11^, ^12^, ^13^, ^14^, ^15^, ^16^, ^17^, ^18^, ^19^, ^20^, ^21^, ^22^, ^23^, ^24^, ^25^, ^26^, ^27^, ^28^, ^29^, ^30^, ^31^, ^32^, ^33^, ^34^, ^35^, ^36^, ^37^, ^38^] They can be covalently attached to the base, sugar, or the phosphates of nucleotides.[^39^, ^40^, ^41^, ^42^] Although long‐living under air and in neutral aqueous buffer, reaction conditions typical for the solid‐phase synthesis of oligonucleotides and for enzymatic ligation steps are known to cause partial decomposition of nitroxides. Accordingly, spin labels are introduced after chain assembly in many cases and even noncovalent attachment is often used.[^7^, ^12^, ^21^, ^22^, ^23^] The most direct way to incorporate spin labels into DNA and RNA, however, is the use of nitroxide modified nucleoside phosphoramidites. If product strands are sufficiently short for HPLC purification, adapted reaction conditions and nitroxides with maximized steric shielding may lead to samples of high quality.^[11]^ As an alternative approach, we have developed light sensitive protecting groups for nitroxides that allowed us to apply standard conditions for solid phase RNA synthesis and enzymatic ligation.[^43^, ^44^, ^45^, ^46^] For example, we have synthesized the phosphoramidite building block **1** (Figure 1) to install the TEMPO spin label[^25^, ^47^, ^48^] in a strand of RNA (**2**).^[45]^ The 2‐nitrobenzyloxymethyl (2‐NBOM) group withstood all chemical and enzymatic steps to generate a 59mer full‐length TAR RNA. After photochemical removal of the nitrobenzyl part and elimination of formic aldehyde, the remaining hydroxylamine spontaneously reacted with air forming the nitroxide radical in excellent yield. Samples of high spectroscopic quality for pulsed electron‐electron double resonance experiments (PELDOR or DEER)[^49^, ^50^, ^51^] were thus obtained without additional purification steps.

Although compound **1** solves the problem of nitroxide instability, TEMPO itself is not the optimal spin label. Attaching this radical to cytidine residues as shown in structure **2** is known to destabilize RNA duplexes by 5–6 °C per modification and up to 10 °C when present in loop‐closing base pairs.^[47]^ A second disadvantage, when compared to rigid nitroxides such as Ç,[^24^, ^52^, ^53^, ^54^, ^55^] is the increased local mobility of TEMPO labels in RNA of type **2**. In contrast, TPA (2,2,5,5‐tetramethyl‐pyrrolin‐1‐oxyl‐3‐acetylene)[^10^, ^56^, ^57^, ^58^, ^59^] present in structure **4** combines good spectroscopic properties with low impact on duplex stabilities. The synthesis of TPA labeled RNA,[^60^, ^61^, ^62^, ^63^, ^64^] however, has been quite complicated in the past. To minimize decomposition of the radical, 5‐iodocytidine was incorporated into RNA strands by solid phase chemistry using the ACE approach that avoids acid induced detritylation and iodine as oxidation agent. In the next step, the support was removed from the synthesizer to attach TPA by manually conducted Sonogashira coupling.

Afterwards, chain extension was resumed on the synthesizer.^[63]^ Samples of excellent quality were obtained but the requirement of a dedicated ACE synthesizer and of chemical skills in the coupling step have limited the application of this method. Here we describe an alternative access to TPA labeled RNA based on the phosphoramidite **3**. As in the case of compound **1**, the photolabile 2‐NBOM group protected the nitroxide and allowed us to assemble RNA strands by unmodified reaction cycles.

## Results and Discussion

**Scheme 1:**
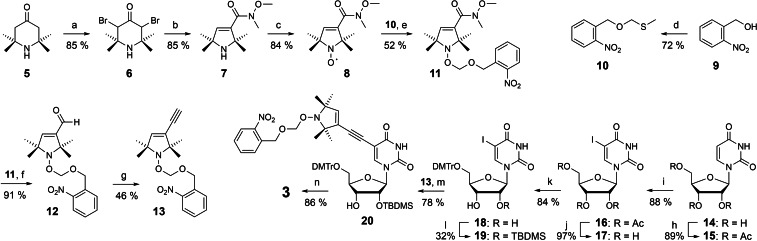
Synthesis of phosphoramidite **3** containing a protected TPA spin label. a) Br_2_, HOAc, 85 %; b) *N*,*O*‐dimethylhydroxylamine hydrochloride, Et_3_N, H_2_O, 85 %; c) *m*‐chloroperbenzoic acid, CH_2_Cl_2_, 84 %; d) DMSO, Ac_2_O, AcOH, then aqueous NaOH, 72 %; e) **10**, SO_2_Cl_2_, then addition of **8**, Cu, Cu(OTf)_2_, 4,4’‐dimethyl‐2,2‐bipyridyl, toluene, Δ, 52 %; f) DIBAL, Et_2_O, toluene, 91 %; g) dimethyl (1‐diazo‐2‐oxopropyl)phosphonate, MeOH, K_2_CO_3_, 46 %; h) Ac_2_O, DMAP, Et_3_N, AcCN, 89 %; i) I_2_, (NH_4_)_2_[Ce(NO_3_)_6_], AcCN, 88 %; j) NaOMe, MeOH, 97 %; k) 4,4’‐dimethoxytrityl chloride, pyridine, 84 %; l) *tert*‐butyldimethylsilyl chloride, DMF, imidazole, 32 %; m) **13**, (Ph_3_P)_4_Pd, CuI, Et_3_N, DMF, 78 %; n) *N*,*N*‐diisopropylaminocyanoethylphosphor amidic chloride, Et_3_N, CH_2_Cl_2_, 86 %.

The synthesis of phosphoramidite **3** is shown in Scheme 1. Compound **5** was converted into Weinreb amide **7** by bromination (**6**) and Favorskii rearrangement. The protected nitroxide **11** was then obtained by *N*‐oxidation (**8**) and reaction with 1‐(chloromethoxymethyl)‐2‐nitrobenzene. This alkyl chloride was prepared in situ from compound **10**, accessible by Pummerer rearrangement from 2‐nitrobenzylic alcohol **9**. The protection of the nitroxide is thought to involve recombination with alkyl radicals generated by reduction of the chloromethoxy‐methyl chain. Such radicals may attack CC triple bonds. We therefore preferred to introduce this group one step later, by DIBAL reduction and conversion of aldehyde **12** with the Ohira‐Bestmann reagent (**13**). Uridine **14** was transformed by a series of standard procedures into the tritylated and 2’ silylated 5‐iodouridine **19**. Sonogashira cross coupling with alkyne **13** and phosphitylation completed the synthesis of compound **3**.

**Figure 2:**
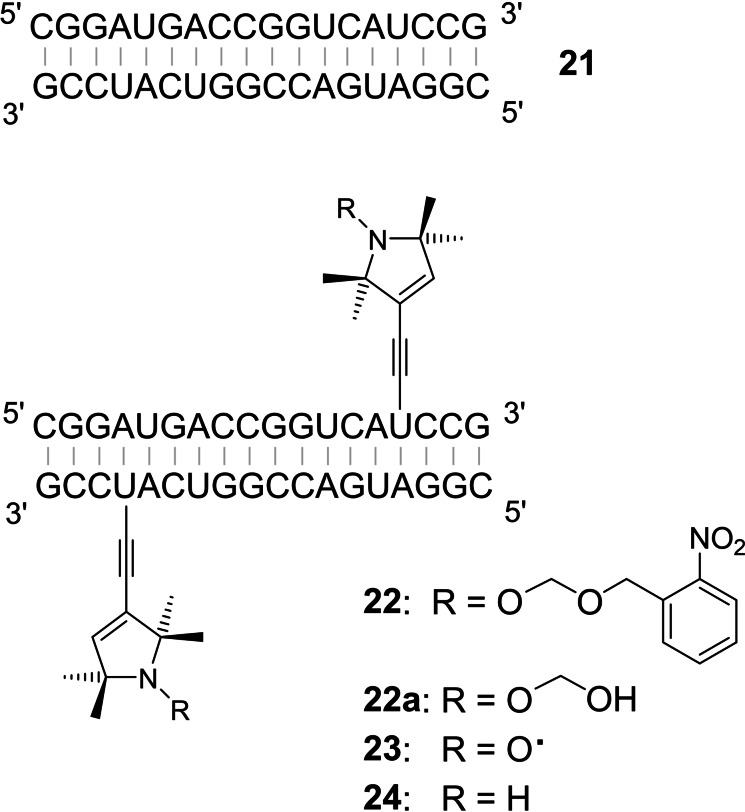
Structure of the palindromic RNAs **21**–**23**, here shown in form of a duplex. In compound **22**, the TPA group is protected by the 2‐NBOM group. Irradiation then leads to hemiacetal **22 a** that eliminates CH_2_O upon heating and forms nitroxide **23** by spontaneous air oxidation. Significant amounts of amine **24**, a possible byproduct that cannot be reoxidized, were not observed.

As a first test, we investigated the palindromic 18mer RNAs **21** and **23** (Figure 2). The chains were assembled on CPG support from 2’‐OTBDMS protected phosphoramidites. Compound **3** reacted uneventfully and did not show signs of decomposition in the subsequent coupling cycles. RNA **22**, containing the protected TPA spin label, was isolated after standard work‐up procedures and purified by HPLC. Mass spectra indicated the presence of an unmodified 2‐NBOM group (Figure S1). The photolabile nitroxide protection was then removed by irradiation of an aqueous solution for 20 min (365 nm; pH 7.4).

**Figure 3:**
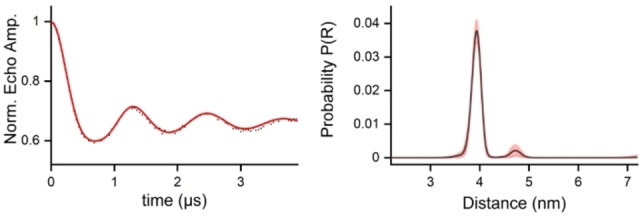
PELDOR measurement of RNA **23**. Left panel: PELDOR time trace after background correction (for original data, see Supporting Information) and fit with DEERNet^[65]^ (red) by DEER analysis.^[66]^ Right panel: Distance distribution with a maximum at 3.94 nm.

Upon heating to 90 °C for 70 min, the resulting hemiacetal **22 a** eliminated CH_2_O and was transformed into the TPA‐labeled RNA **23** by spontaneous air oxidation. The cleavage of light sensitive groups from hydroxylamines can break the weak N−O bond leading to amines. The corresponding product **24**, however, was not detected in the mass spectrum of the crude mixture after irradiation of **21** (Figure S2). Palindromic sequences form duplexes as shown in Figure 2 but may alternatively fold back into monomeric hairpins. Native gel electrophoresis shows, however, that with RNA **23** the duplex structure prevails (Figure S3). When HPLC‐purified RNA **22** was irradiated, oxidized under air and annealed as shown above, such samples of nitroxide labeled RNA could be directly used for PELDOR experiments without additional purification steps (Figure 3). The excellent modulation depth is consistent with a very high labeling degree and the absence of monomeric hairpin structures. PELDOR indicated a spin‐spin distance of 3.94 nm that agrees with the value of 3.81 nm obtained from molecular models (Spartan). In accordance with previous reports,[^61^, ^64^] the TPA spin label present in RNA **23** only marginally interferes with duplex formation: the T_m_ value of **21** (81.5 °C) is reduced to 79.8 °C in case of **23**. Thus two modifications in the duplex cause a ΔT_m_ of only 1.7 °C (Table S2).

**Figure 4:**
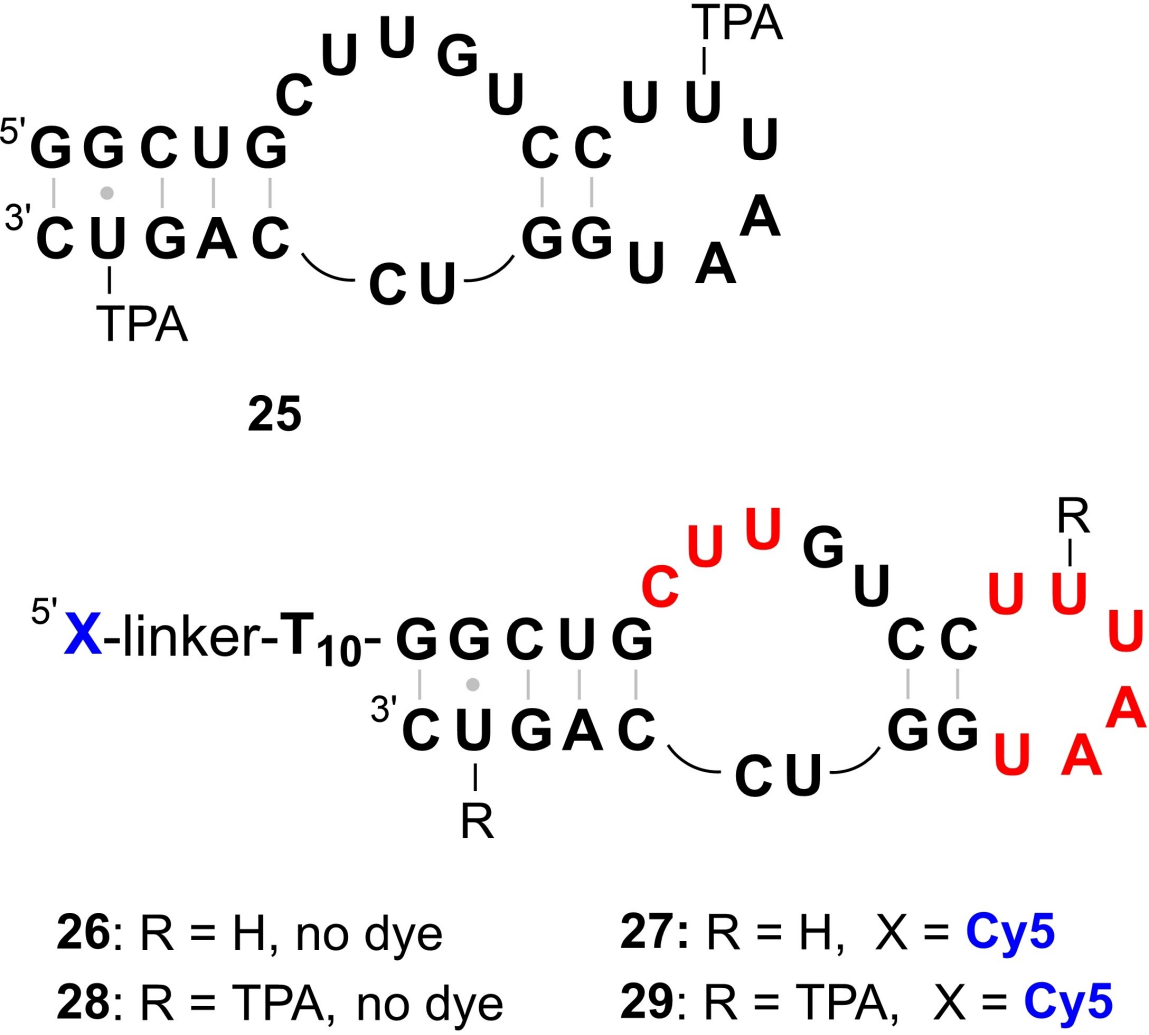
Variants of the neomycin‐responsive riboswitch prepared for this study (for details of structure see Supporting Information). RNA **25** was used for EPR spectroscopy. The dye‐labeled analogs **27** and **29** served as samples for in‐line probing to identify the conformational impact of the TPA labels. The secondary structures are represented as in a previous publication.^[64]^ However, in the unmodified RNA **27**, according to in‐line probing, only nucleotides printed in red show increased mobility.

The EPR investigation of the neomycin‐responsive riboswitch^[67]^ (Figure 4) was one of the first applications of PELDOR on a tertiary folded functional RNA.^[64]^ Composed of 27 nucleotides, this riboswitch is among the smallest known aptamers and can be readily synthesized from phosphoramidite building blocks. In 2010, a series of spin‐labeled derivatives containing one or two TPA groups in different positions was prepared and characterized by PELDOR spectroscopy. The best‐defined spin‐spin distance was observed for compound **25**, modified in positions 14 and 26.^[64]^

**Figure 5:**
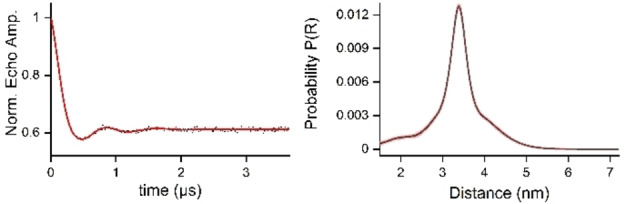
PELDOR measurement of aptamer RNA **25**. Left panel: PELDOR time trace after background correction (for original data, see Supporting Information) and fit with DEERNet^[65]^ (red) by DEER analysis.^[66]^ Right panel: Distance distribution with a maximum at 3.4 nm (sample briefly annealed at 0 °C before freezing).

We have now synthesized riboswitch **25** as described above for RNA **22** from 2’‐OTBDMS protected phosphoramidites and compound **3**. After desilylation and purification by HPLC (Figure S1), the 2‐NBOM groups were removed by irradiation for 20 min (365 nm; pH 7.4). The sample was then heated under air to 90 °C for 70 min to eliminate CH_2_O and to generate the final TPA labeled riboswitch **25** (Figure S2), identical to the material previously reported by Krstić et al.^[64]^ The PELDOR experiment again showed an excellent level of modulation depth and a spin‐spin distance of 3.4 nm, matching exactly the value published previously (Figure 5).

An important issue in EPR studies is the possible impact of sterically demanding spin labels on the structure and dynamics of the target molecule. Duplex melting temperatures are the traditional parameters to assess the overall stability of RNA folds. We have combined T_m_ measurements with in‐line probing as a method which can sensitively record more local effects that may be overlooked if just T_m_ values are considered. Towards this end the riboswitch analogs **26** and **28** were synthesized, purified and, in case of **28**, irradiated to liberate the TPA spin labels. Both RNAs carry a T_10_ tail and an alkyne linker required for the synthesis of the dye labeled analogs **27** and **29**. The T_m_ of **26** (49.5 °C) nevertheless came close to the value of 50.5 °C reported for the unmodified 27mer RNA.^[64]^ The presence of two spin labels in RNA **28** slightly reduced the overall stability (46.0 °C; Ref. [64] for RNA **25**: 48.0 °C). Addition of neomycin increased for both RNAs the values by almost 15 °C. As in the case of the 18mer RNA **23**, these data confirm the low impact of TPA on general duplex stabilities.

**Figure 6:**
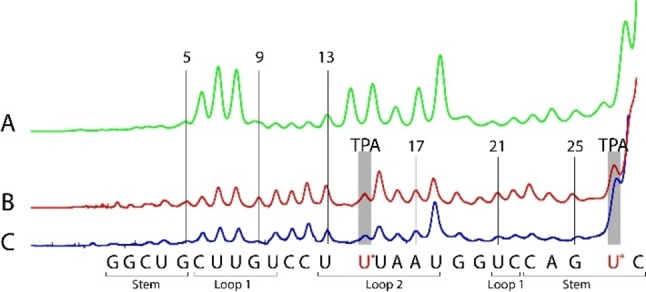
Secondary structure mapping of riboswitch RNAs **27** and **29** by in‐line probing.^[68]^ A: Incubation of RNA **27** at 37 °C (20 h, 20 mM Mg^2+^). Not much cleavage occurs in positions 9–13 indicating the presence of additional base pairs.^[69]^ B: Incubation of RNA **29** at 37 °C (20 h, 20 mM Mg^2+^). The central loop opens up. C: Incubation of RNA **29** at 4 °C (110 h, 20 mM Mg^2+^).

In‐line probing relies on the partial cleavage of 5’ end‐labeled RNA strands in the presence of high concentrations of Mg^2+^.^[68]^ The Lewis‐acidic magnesium ions bind to the phosphates and accelerate the nucleophilic attack by 2’ hydroxy groups thus cleaving strands and forming 2’,3’ cyclic phosphates. The resulting Cy5 labeled fragments are finally separated and quantified by gel electrophoresis using an ALFexpress DNA sequencer.^[45]^ Like in the S_N_2 displacement at carbon, this reaction has the stereoelectronic precondition of an in‐line alignment of nucleophile, electrophile and leaving group not given in regular helical duplexes. Cleavage is therefore restricted to positions of increased conformational mobility such as bulges and loops. In riboswitch RNAs **25**–**29** the first five nucleotides are part of a stable stem structure. Accordingly, not much cleavage is seen for these nucleotides upon treatment of the dye‐labeled RNA **27** with Mg^2+^ (Figure 6 trace A: incubation at 37 °C). Strong cleavage occurs after C(6), U(7), U(8) and in the loop region from U(13) to U(18). Interestingly, the peaks from G(9) to U(10) and U(21) to C(22) are weak. The fold shown in Figure 4 was derived from enzymatic secondary structure mapping in absence of neomycin.^[67]^ In contrast, in‐line probing indicates restricted conformational mobility of G(9) and U(10), presumably by forming base pairs with U(21) and C(22). This type of conformation, coaxial stacking of two helices while C(6) to U(8) are expelled as a bulge, was found previously by enzymatic probing and NMR spectroscopy in aminoglycoside complexes of RNA **25**.^[69]^

The presence of TPA labels in RNA **29**, however, distinctly changes the picture (Figure 6 trace B: incubation at 37 °C). First of all, the fragment ending with a TPA modified U(14) runs slower than the unmodified fragment due to its bigger size. The peak distance between U(13) and TPA−U(14) therefore is roughly twice as large. More important, the cleavage in most positions after U(8) is increased: Compared to RNA **27**, the fold of the spin‐labeled analog **29** is significantly destabilized. This effect can be reversed to some extent when in‐line probing is conducted at 4 °C (Figure 6 trace C): The RNA is still less structured as RNA **27** but the amount of cleavage declines after G(9) and after the nucleotides following U(18). Along this line, we also found that brief annealing of PELDOR samples at 0 °C before freezing resulted in a sharper spin‐spin distance distribution within RNA **25** (compare Figures S9 and S10a).

## Conclusion

Increased rigidity and reduced steric perturbation of nucleic acid structures are the advantages of TPA spin labels over TEMPO‐modified RNA (compare labeling types **4** and **2** in Figure 1). The complex and demanding synthesis of RNA samples, however, has prevented a broad use of this approach in the past. Protection of the nitroxide with a 2‐nitrobenzyloxymethyl group in phosphoramidite **3** now offers access to TPA labeled RNAs in a faster and more reliable way. Light induced liberation of nitroxide radicals[^70^, ^71^] has meanwhile found interesting applications for site‐directed spin labeling of proteins[^72^, ^73^] and also in surface chemistry.^[74]^ As an alternative to photosensitive residues, Sigurdsson recently used benzoyl groups to protect nitroxides during RNA chain assembly with phosphoramidite building blocks.[^24^, ^75^, ^76^] Benzoyl is removed after solid‐phase synthesis in the aminolysis step. Although the concept is simple and effective, it does not protect the RNA if subsequent critical steps such as enzymatic ligations are required.

The rigid Ç label and even TEMPO do not significantly change the base pair arrangements in simple RNA duplexes.^[77]^ However, when less stable folds are considered, the risk of perturbation by spin labels will increase. Even in HIV‐TAR, a robust stem‐loop structure of 59 nucleotides, in‐line probing showed increased mobility around the UCU bulge when a TEMPO label was installed in close proximity.^[45]^ Although T_m_ values suggested no major impact of the TPA labels in riboswitch RNA (**27** versus **29**), in‐line probing still detected undeniable local effects. Accordingly, it is highly recommended to combine EPR studies of RNAs with additional secondary structure mapping to rule out major spin label‐induced perturbations.

## Conflict of interest

The authors declare no conflict of interest.

1

## Supporting information

As a service to our authors and readers, this journal provides supporting information supplied by the authors. Such materials are peer reviewed and may be re‐organized for online delivery, but are not copy‐edited or typeset. Technical support issues arising from supporting information (other than missing files) should be addressed to the authors.

Supporting Information

## Data Availability

The data that support the findings of this study are available in the supplementary material of this article.
