# Supplementary material for: Spin‐Labeled Riboswitch Synthesized from a Protected TPA Phosphoramidite Building Block
Source: Chemistry. 2022 Aug 18;28(56):e202201822. doi: 10.1002/chem.202201822 (PMC9804336; doi:10.1002/chem.202201822)

Chemistry–A European Journal

Supporting Information

Spin-Labeled Riboswitch Synthesized from a Protected TPA Phosphoramidite Building Block

Frank Kaiser, Burkhard Endeward, Alberto Collauto, Ute Scheffer, Thomas F. Prisner, and Michael W. Göbel*

Supporting Information

Table of contents:

General.. ......S2
Synthesis of phosphoramidite **3**..S3
Synthesis, purification and quantification of oligonucleotides..S13
Photochemical deprotection...S16
Duplex versus hairpin structure of oligonucleotide **23**..S17
Cy5-labeling and inline-probing..S18
EPR measurements...S22
Determination of duplex stabilities...S25
References...S29
NMR spectra...S30

General

All critical steps were conducted under inert gas (argon 5.0). Glassware was dried with a heatgun in an oil pump vacuum and, after cooling to room temperature, charged with argon. The solvents for the synthesis were obtained in *p.a*. quality and used without further purification. Solvents used in reactions under oxygen-free conditions were obtained from Acros in *p.a*. quality and stored under exclusion of air und moisture via molecular sieve. Flash column chromatography was carried out with solvents of high technical quality. The silica gel (60 Å pore size, 40 – 63 µm particle size) and the TLC plates (ALUGRAM Xtra SIL G/UV_{254}) were obtained from Macherey-Nagel. ESI-MS spectra were recorded on a Thermo Fisher Scientific Surveyor MSQ and the HRMS spectra were obtained on a Thermo-Scientific MALDI-LTQ-Orbitrap XL. Oligonucleotides were analyzed via ESI mass spectrometry using a LCMS instrument with microTOF-Q II analyser (Bruker). An Agilent 1200 Series HPLC was applied as LC system. Characterization by NMR was done in deuterated solvents (DMSO-d_6 and $CDCl_3$) using an Avance-400 spectrometer from Bruker. Proton nuclear magnetic resonance (^{1}H-NMR) spectra, carbon nuclear magnetic resonance (^{13}C-NMR) spectra, silicon nuclear magnetic resonance (^{29}Si-NMR) spectra and phosphorus nuclear magnetic resonance (^{31}P-NMR) were recorded at 300 K (^{1}H-NMR: 400.1 MHz; ^{13}C-NMR: 100.6 MHz; ^{29}Si-NMR: 79.5 MHz; ^{31}P-NMR: 161.9 MHz). Chemical shifts are given as δ-value in parts per million (ppm) and coupling constants are given in Hertz (Hz). The observed resonances were calibrated against the residual proton content of the solvents (secondary standard: δ = 7.26 as singlet for $CHCl_3$ and δ = 2.50 as quintet for DMSO-d_5 in the ^{1}H-NMR; δ = 77.16 as triplet for $CDCl_3$ and δ = 39.52 as septet for DMSO-d_6 in the ^{13}C-NMR) relative to the resonance of tetramethylsilane (primary standard: δ = 0.00). The assignment of the individual signals in the ^{1}H-NMR is based on ^{1}H-^{1}H-COSY. Data are represented as follows: chemical shift, multiplicity (s = singlet, bs = broad singlet, d = doublet, dd = double doublet, t = triplet, m = multiplet), coupling constants and integration. Melting points were determined twice on a Kofler hot stage microscope.

Hydrobromide of *meso*-3,5-dibromo-2,2,6,6-tetramethylpiperidin-4-one (**6**)

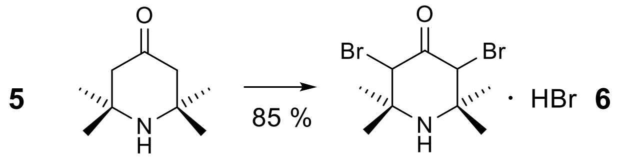

The procedure is based on the method of Wu *et al.*[1]

To a solution of 2,2,6,6-tetramethylpiperidin-4-one (**5**) (15.00 g, 96.63 mmol, 1.0 eq.) in 100 mL glacial acetic acid was slowly added a solution of bromine (9.90 mL, 193.25 mmol, 2.0 eq.) in 75 mL glacial acetic acid. The mixture was stirred over night at room temperature. Afterwards the solid was filtered off and washed with glacial acetic acid (100 mL), water (50 mL) and diethyl ether (50 mL). After drying in air, product **6** was obtained as a colorless hydrobromide salt (85%, 32.45 g, 82.37 mmol) and used without further purification. ^{1}H-NMR (400 MHz, DMSO-d$_6$): 5.48 (s, 2H, H-3/H-5), 1.70 (s, 6H, methyl), 1.35 (s, 6H, methyl) ppm. ^{13}C-NMR (100 MHz, DMSO-d$_6$): 188.9, 64.3, 60.2, 27.8, 22.1 ppm. MS (ESI): *m/z*: 312.06, 314.06, 316.06 [M+H$^+$]. HRMS: calcd. for $C_9H_{16}Br_2NO$ 311.95986, 313.95782, 315.95577 [M+H$^+$]; found 311.95954, 313.95751, 315.95524.

2,5-Dihydro-*N*-methoxy-*N*,2,2,5,5-pentamethyl-1*H*-pyrrole-3-carboxamide (**7**)

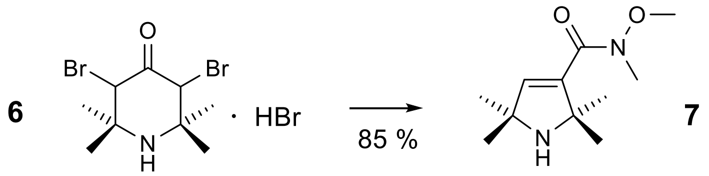

To a solution of *N,O*-dimethylhydroxylamine hydrochloride (8.91 g, 91.38 mmol, 1.25 eq.) in 50 mL water at 0 °C was added 50 mL triethylamine. At room temperature, compound **6** (28.80 g, 73.11 mmol, 1.0 eq.) was added in portions under vigorous stirring. The reaction mixture was stirred over night at room temperature. After addition of ethyl acetate, the aqueous phase was extracted four times with ethyl acetate. The combined organic phases were dried over magnesium sulphate and the solvent was removed under reduced pressure. The crude product was fractionally distilled (88 °C and <1 mbar). The product **7** was obtained as a yellow liquid (85%, 13.22 g, 62.15 mmol). Alternatively, a purification by silica gel chromatography is also possible (dichloromethane/methanol 19:1). R_f = 0.45. ^{1}H-NMR (400 MHz, CDCl$_3$): 6.05 (s, 1H, H-4), 4.33 (bs, 1H, -NH), 3.55 (s, 3H, -OCH$_3$), 3.16 (s, 3H, -NCH$_3$), 1.42 (s, 6H, methyl), 1.31 (s, 6H, methyl) ppm. ^{13}C-NMR (100 MHz, CDCl$_3$): 165.7, 140.1, 138.4, 69.3,

65.2, 61.0, 33.2, 29.7, 29.4 ppm. MS (ESI): *m/z*: 213.28 [M + H$^+$]. HRMS: calcd. for $C_{11}H_{21}N_2O_2$ [M + H$^+$]: 213.15975; found 213.15974.

2,5-Dihydro-3-[(*N*-methoxy-*N*-methylamino)carbonyl]-2,2,5,5-tetramethyl-1*H*-pyrrol-1-yloxy (**8**)

7

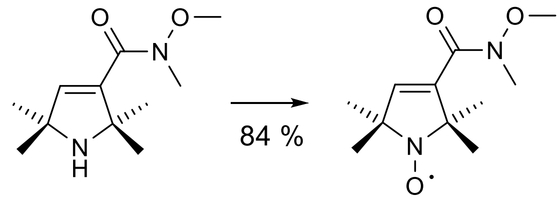

8

Compound **7** (13.22 g, 62.27 mmol, 1.0 eq.) was dissolved in 110 mL dichloromethane and cooled to 0 °C. *meta*-Chloroperoxybenzoic acid (27.91 g, 124.55 mmol, 2.0 eq., 77% purity) was added in portions. After completion of the addition, the reaction mixture was stirred for another 60 min at room temperature. Afterwards the suspension was washed with 200 mL of 20% potassium hydroxide solution. The aqueous phase was extracted twice with dichloromethane and the combined organic phase was dried over magnesium sulphate. The solvent was removed under reduced pressure. The title compound **8** was obtained as yellow needles (84%, 11.95 g, 52.58 mmol) after silica gel chromatography (cyclohexane/ethyl acetate 2:1) and recrystallisation from cyclohexane. R_f = 0.77 (dichloromethane/methanol 19:1). Mp: 57 °C (Lit: 56.0 – 56.5 °C[2]). For NMR experiments, phenyl hydrazine was added to reduce the radical to the amine. ^{1}H-NMR (400 MHz, $CDCl_3$): 6.02 (s, 1H, H-4), 3.54 (s, 3H, -OCH_3), 3.15 (s, 3H, -NCH_3), 1.35 (s, 6H, methyl), 1.25 (s, 6H, methyl) ppm. ^{13}C-NMR (100 MHz, $CDCl_3$): 165.5, 138.1, 136.8, 72.9, 69.3, 61.22, 33.4, 24.9, 24.7 ppm. EA: calcd. for $C_9H_{19}N_2O_3$ C: 58.13, H: 8.43, N: 12.33; found C: 58.31, H: 8.59, N: 12.17.

1-[([Methylthio]methoxy)methyl]-2-nitrobenzene (**10**)

9

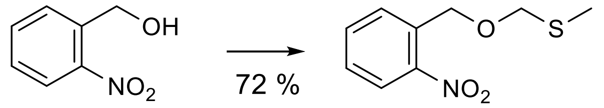

10

The procedure is based on the method of Yong *et al.*[3]

o-Nitrobenzyl alcohol **9** (5.00 g, 32.65 mmol, 1.0 eq.) was dissolved in 75 mL glacial acetic acid. Dimethyl sulfoxide (47.0 mL, 650 mmol, 20.0 eq.) was added followed by slow addition of acetic

anhydride (61.7 mL, 650 mmol, 20.0 eq., room temperature). The reaction mixture was stirred for 72 h at room temperature. The reaction mixture was transferred to a funnel and slowly added to a 10 M aqueous KOH solution cooled to 0 °C. After the addition was completed, the reaction mixture was stirred for two more hours at room temperature. The phases were separated and the aqueous phase extracted three times with diethyl ether. The combined organic phases were dried over magnesium sulphate and the solvent removed under reduced pressure, followed by silica gel chromatography (cyclohexane/ethyl acetate 10:1). Compound **10** was obtained as a yellow oil (72%, 5.00 g, 23.45 mmol). R_f = 0.51 (cyclohexane/ethyl acetate 10:1). ^{1}H-NMR (400 MHz, $CDCl_3$): 8.08 – 8.05 (m, 1H, H-3), 7.77 – 7.76 (m, 1H, H-6), 7.66 – 7.62 (m, 1H, H-5), 7.47 – 7.43 (m, 1H, H-4), 4.99 (s, 2H, nitrophenyl-CH_2-O-), 4.78 (s, 2H, -O-CH_2-S-), 2.20 (s, 3H, methyl) ppm. ^{13}C-NMR (100 MHz, $CDCl_3$): 134.5, 133.7, 129.1, 128.3, 124.9, 75.7, 66.8, 14.4 ppm. MS (ESI): *m/z*: 213.27 [M + H$^+$]. HRMS: calcd. for $C_9H_{11}NO_3S$ [M + Na$^+$]: 236.03519; found 236.03403.

N-Methoxy-*N*,2,2,5,5-pentamethyl-1-[([2-nitrobenzyl]oxy) methoxy]-2,5-dihydro-1*H*-pyrrole-3-carboxamide (**11**)

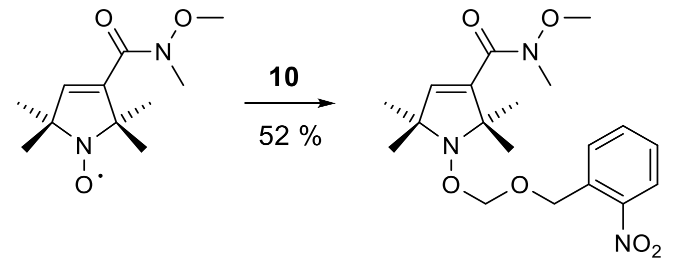

The procedure is based on the method of Weinrich *et al.*[4]

To a solution of *o*-nitrobenzyl methylthiomethyl ether (**10**) (1.00 g, 4.68 mmol, 1.5 eq) in 15 mL anhydrous dichloromethane was added dropwise sulfuryl chloride (0.44 mL, 5.39 mmol, 1.7 eq.). After the reaction solution was stirred for 60 min at room temperature, saturated sodium hydrogen carbonate solution was added. The organic solution was washed three times with saturated sodium hydrogen carbonate solution and once with water. The organic phase was then dried over magnesium sulphate and the solvent removed under reduced pressure (max. 30 °C water bath temperature). The yellow oil obtained was dissolved in 10 mL anhydrous toluene and added to a preheated mixture of compound **8** (710 mg, 3.12 mmol, 1.0 eq.), copper powder (300 mg, 4.68 mmol, 1.5 eq.), copper(II) trifluoromethanesulfonate (60 mg, 0.16 mmol, 0.05 eq.) and 4,4'-dimethyl-2,2'-bipyridyl (85 mg, 468 µmol, 0.15 eq.) in 20 mL anhydrous toluene. The reaction suspension was degassed and heated to reflux for 21 h under argon. The suspension was filtered through silica gel and leftovers eluted with dichloromethane/methanol 3:1. The solvent was removed under reduced pressure. Purification by

silica gel chromatography (cyclohexane/ethyl acetate 6:1) gave the title compound **11** as a bright yellow oil (52%, 638 mg, 2.43 mmol) which solidified after standing. R_f = 0.35 (cyclohexane/ethyl acetate 5:1). ^{1}H-NMR (400 MHz, $CDCl_3$): 8.11 (d, ^3J = 8.2 Hz, 1H, nitrophenyl H-3), 7.87 (d, ^3J = 7.8 Hz, 1H, nitrophenyl H-6), 7.65 (t, ^3J = 7.4 Hz, 1H, nitrophenyl H-5), 7.44 (t, ^3J = 8.1 Hz, 1H, nitrophenyl H-4), 6.01 (s, 1H, pyrrole H-4), 5.14 (s, 2H, nitrophenyl-CH_2-O-), 5.06 (s, 2H, -O-CH_2-O-), 3.63 (s, 3H, -O-CH_3), 3.24 (s, 3H, -N-CH_3), 1.46 – 1.31 (m, 12H, pyrrole CH_3) ppm. ^{13}C-NMR (100 MHz, $CDCl_3$): 165.9, 147.2, 138.1, 135.0, 133.9, 128.8, 128.1, 124.9, 100.6, 67.7, 61.2, 33.6, 23.6, 22.8 ppm. MS (ESI): *m/z*: 394.37 [M + H^+]. HRMS: calcd. for $C_{19}H_{28}N_3O_6$ [M + H^+]: 394.19726; found 394.19722.

2,2,5,5-Tetramethyl-1-[([2-nitrobenzyl]oxy)methoxy]-2,5-dihydro-1*H*-pyrrole-3-carbaldehyde (12)

11

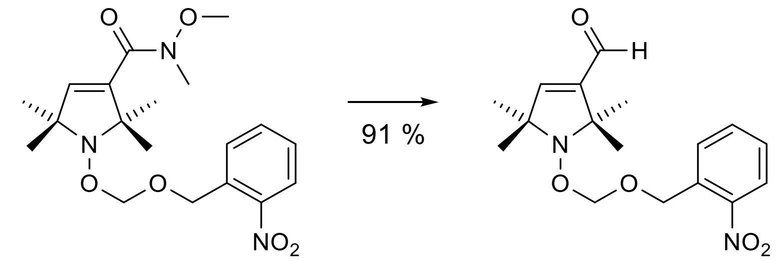

12

To a solution of compound **11** (2.06 g, 5.23 mmol, 1.0 eq.) in 40 mL anhydrous diethyl ether cooled to -78 °C, DIBAL-H in toluene (8.70 mL, 10.47 mmol, 1.2 M, 2.0 eq.) was added dropwise. After the reaction was stirred for 45 min at -78 °C, it was poured slowly into 100 mL of ice-cooled diluted hydrochloric acid. The phases were separated and the aqueous phase was extracted twice with ethyl acetate. The combined organic phase was dried over magnesium sulphate and the solvent removed under reduced pressure. The product **12** was obtained after silica gel chromatography (cyclohexane/ethyl acetate 8:1) as a colourless oil which solidified after standing (91%, 1.60 g, 4.79 mmol). R_f = 0.80 (cyclohexane/ethyl acetate 8:1). ^{1}H-NMR (400 MHz, $CDCl_3$): 9.61 (s, 1H, -CHO), 8.11 (dd, ^4J = 1.2 Hz, ^3J = 8.2 Hz, nitrophenyl H-3), 7.87 – 7.85 (m, 1H, nitrophenyl H-6), 7.66 (td, ^4J = 1.2 Hz, ^3J = 7.8 Hz, nitrophenyl H-5), 7.47 – 7.43 (m, 1H, nitrophenyl H-4), 6.51 (s, 1H, pyrrole H-4), 5.12 (s, 2H, nitrophenyl-CH_2-O-), 5.03 (s, 2H, -O-CH_2-O-), 1.43 – 1.33 (m, 12H, pyrrole CH_3) ppm. ^{13}C-NMR (100 MHz, $CDCl_3$): 188.9, 154.1, 147.2, 145.7, 134.9, 133.9, 128.7, 128.1, 125.0, 100.8, 69.5, 68.8, 67.6, 28.7, 27.9, 22.7, 22.4 ppm. MS (ESI): *m/z*: 335.23 [M + H^+]. HRMS: calcd. for $C_{17}H_{22}N_2O_5$ [M + H^+]: 335.16015; found 335.16024.

3-Ethynyl-2,2,5,5-tetramethyl-1-(((2-nitrobenzyl)oxy)methoxy)-2,5-dihydro-1*H*-pyrrole (**13**)

12 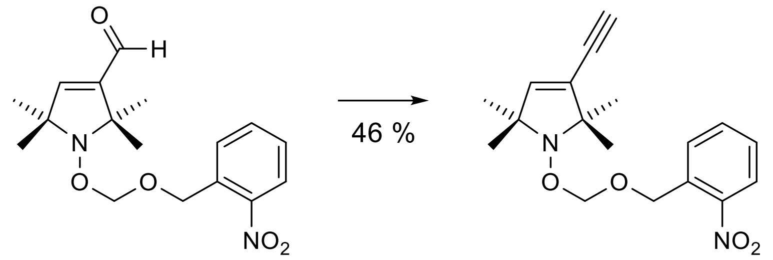 **13**

Aldehyde **12** (1.50 g, 4.49 mmol, 1.0 eq.) was dissolved in 60 mL anhydrous methanol. Potassium carbonate (3.10 g, 22.43 mmol, 5.0 eq.) and Bestmann Ohira reagent (3.02 g, 15.70 mmol, 3.5 eq.) were added successively. The reaction was stirred over night at room temperature and diluted with 50 mL diethyl ether. The organic phase was washed four times with saturated sodium chloride solution, dried over magnesium sulphate and the solvent was removed under reduced pressure. Purification by silica gel chromatography (cyclohexane/ethyl acetate 15:1) gave the title compound **13** as a colorless solid (46%, 0.68 g, 2.07 mmol). R_f = 0.62 (cyclohexane/ethyl acetate 9:1). ^{1}H-NMR (400 MHz, $CDCl_3$): 8.11 (dd, ^3J = 8.2 Hz, ^4J = 1.1 Hz, 1H, nitrophenyl H-3), 7.87 (d, ^3J = 7.9 Hz, 1H, nitrophenyl H-6), 7.66 (td, ^3J = 7.4 Hz, ^4J = 1.1 Hz, 1H, nitrophenyl H-5), 7.45 (t, ^3J = 8.1 Hz, 1H, nitrophenyl H-4), 5.86 (s, 1H, pyrrole H-4), 5.12 (s, 2H, nitrophenyl-CH_2-O-), 5.02 (s, 2H, -O-CH_2-O-), 2.99 (s, 1H, ethynyl-H), 1.34 – 1.23 (m, 12H, pyrrole CH_3) ppm. ^{13}C-NMR (100 MHz, $CDCl_3$): 147.2, 141.0, 135.1, 133.9, 128.7, 128.1, 126.9, 124.9, 100.6, 80.3, 78.3, 71.6, 69.0, 67.6, 29.3, 28.4, 23.3, 22.8 ppm. MS (ESI): *m/z*: 331.27 [M + H$^+$]. HRMS: calcd. for $C_{18}H_{23}N_2O_4$ [M + H$^+$]: 331.16578; found 331.16532.

2ʹ,3ʹ,5ʹ-Tri-*O*-acetyluridine (**15**)

14 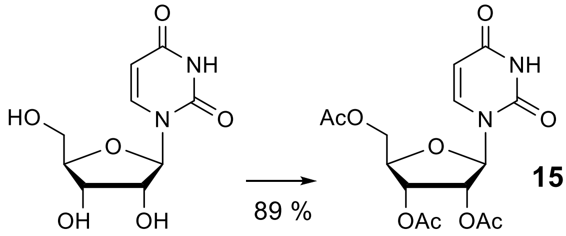 **15**

To a suspension of uridine **14** (4.88 g, 19.98 mmol, 1.0 eq.) in 100 mL acetonitrile, triethylamine (22.3 mL, 159.9 mmol, 8.0 eq.), acetic anhydride (15.1 mL, 159.9 mmol, 8.0 eq.) and a catalytic amount of 4-(dimethylamino)pyridine were added. The solution was stirred over night at room temperature. Dichloromethane and water were added and the organic phase was washed four times with water.

The organic phase was dried over magnesium sulphate and the solvent removed under reduced pressure. Purification by silica gel chromatography (dichloromethane/methanol 97:3) and recrystallization from ethanol gave compound **15** as colourless crystals (89%, 6.51 g, 17.58 mmol). R_f = 0.30 (dichloromethane/methanol 97:3). Mp: 133 °C (Lit: 130 – 132 °C[5]). ^{1}H-NMR (400 MHz, $CDCl_3$): 9.41 (bs, 1H, -NH), 7.38 (d, ^3J = 8.1 Hz, 1H, H-6), 6.04 – 6.03 (m, 1H, H-1'), 5.78 (d, ^3J = 8.1 Hz, 1H, H-5), 5.35 – 5.30 (m, 2H, H-2', H-3'), 4.36 – 4.29 (m, 3H, H-4', H-5'/5''), 2.13 (s, 3H, -OAc), 2.12 (s, 3H, -OAc), 2.09 (s, 3H, -OAc) ppm. ^{13}C-NMR (100 MHz, $CDCl_3$): 170.3, 169.8, 163.0, 150.4, 139.4, 103.6, 87.6, 80.1, 72.8, 70.3, 63.3, 20.9, 20.6, 20.5 ppm. MS (ESI): *m/z*: 371.25 [M + H$^+$]. HRMS: calcd. for $C_{15}H_{18}N_2O_9Na$ [M + Na$^+$]: 393.09045; found 393.09031.

2',3',5'-Tri-*O*-acetyl-5-iodouridine (**16**)

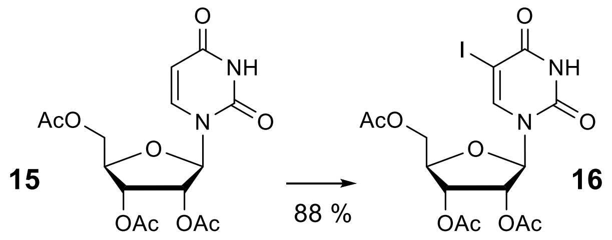

The procedure is based on a method of Asakura and Robins.[6]

A solution of compound **15** (5.73 g, 15.47 mmol, 1.0 eq.), iodine (2.36 g, 9.28 mmol, 0.6 eq.) and ammonium cerium(IV)nitrate (4.24 g, 7.74 mmol, 0.5 eq.) in 250 mL acetonitrile was stirred for 90 min at 80 °C. Afterwards the solvent was removed under reduced pressure and the residue partitioned between a mixture of ethyl acetate (100 mL), brine (50 mL) and saturated sodium thiosulphate solution (25 mL). The aqueous phase was extracted twice with ethyl acetate (50 mL). After the combined organic phase was washed with brine and water, dried over magnesium sulphate and the solvent was removed under reduced pressure. Product **16** was obtained as colorless crystals (88%, 6.76 g, 13.62 mmol) after recrystallization from ethanol. Mp: 175 °C (Lit: 177 – 178 °C[7]). ^{1}H-NMR (400 MHz, $CDCl_3$): 9.46 (bs, 1H, -NH), 7.88 (s, 1H, H-6), 6.07 – 6.04 (m, 1H, H-1'), 5.34 – 5.30 (m, 2H, H-2', H-3'), 4.40 – 4.31 (m, 3H, H-4', H-5'/5''), 2.22 (s, 3H, -OAc), 2.11 (s, 3H, -OAc), 2.09 (s, 3H, -OAc) ppm. ^{13}C-NMR (100 MHz, $CDCl_3$): 170.2, 169.75, 169.74, 159.7, 150.1, 143.9, 87.3, 80.4, 73.2, 70.3, 69.8, 63.1, 21.2, 20.6, 20.5 ppm. Signals agree with literature values. MS (ESI): *m/z*: 497.18 [M + H$^+$]. HRMS: calcd. for $C_{15}H_{17}IN_2O_9Na$ [M + Na$^+$]: 518.98709; found 518.98624.

5-Iodouridine (**17**)

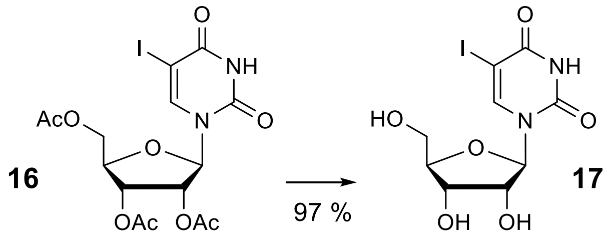

The procedure is based on a method of Meneghesso *et al.*[8]

To a solution of compound **16** (12.74 g, 25.67 mmol, 1.0 eq.) in 200 mL anhydrous methanol was added dropwise a solution of sodium methoxide (5.40 M, 14.3 mL, 77.0 mmol, 3.0 eq.) in methanol. The reaction was stirred for 120 min at room temperature and then neutralized with Dowex® 50WX8 50 – 100(H). After filtration, the solvent was removed under reduced pressure. The crude product was purified by recrystallization from ethanol to obtain a colorless solid of **17** (97%, 9.23 g, 24.94 mmol). Mp: 190 °C decomposition (Lit: 205 – 210 °C decomposition[7]). ^{1}H-NMR (400 MHz, DMSO-d$_6$): 11.66 (bs, 1H, -NH), 8.47 (s, 1H, H-6), 5.72 (d, ^{3}J = 4.7 Hz, 1H, H-1'), 5.39 (d, ^{3}J = 5.2 Hz, 1H, 2'-OH), 5.24 (t, ^{3}J = 4.6 Hz, 1H, 5'-OH), 5.05 (d, ^{3}J = 4.9 Hz, 1H, 3'-OH), 4.05 – 4.04 (m, 1H, H-2'), 3.99 – 3.96 (m, 1H, H-3'), 3.87 – 3.85 (m, 1H, H-4'), 3.70 – 3.65 (m, 1H, H-5'/5''), 3.59 – 3.54 (m, 1H, H-5'/5'') ppm. ^{13}C-NMR (100 MHz, DMSO-d$_6$): 160.5, 150.4, 145.1, 88.3, 84.7, 73.9, 69.4, 69.3, 60.2 ppm. Signals agree with literature values. MS (ESI): *m/z*: 369.22 [M - H$^+$]. HRMS: calcd. for $C_9H_{11}IN_2O_6Na$ [M + Na$^+$]: 392.95540; found 392.95536.

5'-O-[Bis(4-methoxyphenyl)phenylmethyl]-5-iodouridine (**18**)

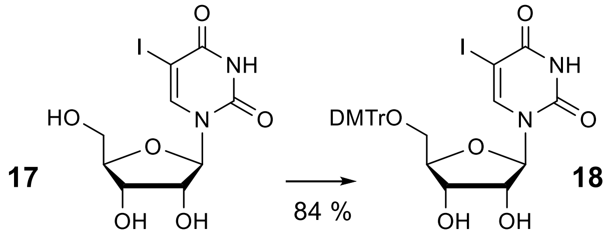

To a solution of 5-iodouridine **17** (8.50 g, 22.97 mmol, 1.0 eq.) in 150 mL anhydrous pyridine were added five servings 4,4'-dimethoxytrityl chloride (10.12 g, 29.86 mmol, 1.3 eq.) 30 min apart. The solution was stirred for another 60 min at room temperature. The solvent was then removed under reduced pressure (max. 30 °C water bath temperature) and the residue co-evaporated several times

with toluene. The residue was partitioned between ethyl acetate and saturated sodium hydrogen carbonate solution (v/v: 1:1). After the organic phase was washed with saturated sodium hydrogen carbonate solution, brine and water, it was dried over magnesium sulphate and the solvent was removed under reduced pressure (max. 30 °C water bath temperature). Purification by silica gel chromatography (acetone/cyclohexene/triethylamine 90:10:1) gave the desired product as a white foam (84%, 12.95 g, 19.26 mmol). R_f = 0.45 (eluent see above). ^{1}H-NMR (400 MHz, DMSO-d$_6$): 11,75 (s, 1H, -NH), 8.00 (s, 1H, H-6), 7.42 – 7.40 (m, 2H, trityl-H), 7.34 – 7.28 (m, 6H, trityl-H), 7.25 – 7.21 /m, 1H, trityl-H), 6.91 – 6.89 (m, 4H, trityl-H), 5.74 (d, ^3J = 5.4 Hz, 1H, H-1'), 5.45 (d, ^3J = 5.6 Hz, 1H, 2'-OH), 5.13 (d, ^3J = 5.6 Hz, 1H, 3'-OH), 4.19 (q, ^3J = 5.4 Hz, 1H, H-2'), 4.03 (q, ^3J = 4.9 Hz, 1H, H-3'), 3.98 – 3.95 (m, 1H, H-4'), 3.74 (s, 6H, -OCH$_3$), 3.23 – 3.15 (m, 2H, H-5'/5'') ppm. ^{13}C-NMR (100 MHz, DMSO-d$_6$): 160.4, 158.1, 150.3, 144.7, 144.4, 135.5, 135.4, 129.7, 128.0, 127.7, 126.7, 113.2, 88.6, 85.9, 83.1, 73.2, 70.0, 63.5, 55.1 ppm. MS (ESI): *m/z*: 673.10 [M + H$^+$]. HRMS: calcd. for $C_{30}H_{29}IN_2O_8Na$ [M + Na$^+$]: 695.08608; found 695.08634.

5'-*O*-[Bis(4-methoxyphenyl)phenylmethyl]-2'-O-[(1,1-dimethylethyl)dimethylsilyl]-5-iodouridine (**19**)

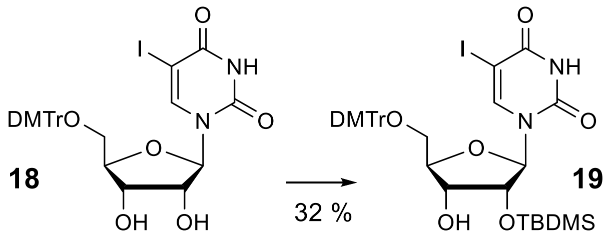

Compound **18** (10.00 g, 14.87 mmol, 1.0 eq.) and imidazole (8.10 g, 118.97 mmol, 8.0 eq.) were dissolved in 250 mL anhydrous dimethylformamide followed by slow addition of *tert*-butyldimethylsilyl chloride (3.59 g, 23.79 mmol, 1.6 eq.). The reaction mixture was stirred for 22 h at room temperature. Afterwards 250 mL of saturated sodium hydrogen carbonate solution was added and the solution extracted with dichloromethane. The organic phase was dried over magnesium sulphate and the solvent removed under reduced pressure (max. 30 °C water bath temperature). Purification by silica gel chromatography (cyclohexane/acetone 3:1 + 1% triethylamine) gave the title compound **19** as a colorless foam (32%, 3.70 g, 4.70 mmol). The 3'-*O*-TBDMS isomer and the bisilylated product could be deprotected with 1 M tetrabutylammonium fluoride solution and used again after silica gel chromatography (acetone/cyclohexane/triethylamine 90:10:1). R_f = 0.25 (acetone/cyclohexane/ triethylamine 90:10:1). ^{1}H-NMR (400 MHz, DMSO-d$_6$): 11.81 (bs, 1H, -NH), 8.05 (s, 1H, pyrimidine-H),

7.41 – 7.39 (m, 2H, trityl-H), 7.34 – 7.28 (m, 6H, trityl-H), 7.25 – 7.22 (m, 1H, trityl-H), 6.91 – 6.89 (m, 4H, trityl-H), 5.81 (d, ^3J = 5.6 Hz, 1H, H-1’), 5.14 (d, ^3J = 5.8 Hz, 1H, 3’-OH), 4.31 (t, ^3J = 5.3 Hz, 1H, H-2’), 4.02 – 4.00 (m, 1H, H-4’), 3.98 – 3.95 (m, 1H, H-3’), 3.74 (s, 6H, -OCH_3), 3.23 – 3.22 (m, 2H, H-5’/5’’), 0.84 (s, 9H, -$SiC(CH_3)_3$), 0.05 (s, 3H, -$SiCH_3$), 0.03 (s, 3H, -$SiCH_3$) ppm. ^{13}C-NMR (100 MHz, DMSO-d_6): 160.5, 158.3, 150.3, 144.8, 143.9, 143.8, 135.32, 135.28, 129.8, 128.4, 128.1, 127.6, 126.93, 126.91, 113.4, 87.9, 86.1, 83.7, 75.5, 70.2, 70.0, 63.4, 55.2, 31.5, 25.7, 18.0, -4.8, -5.2 ppm. ^{29}Si-NMR (79.5 MHz, DMSO-d_6): 21.6 ppm. MS (ESI): *m/z*: 785.46 [M - H^+]. HRMS: calcd. for $C_{36}H_{43}IN_2O_8SiNa$ [M + Na^+]: 809.17256; found 809.17511.

Nucleoside **20** with protected TPA spin label

19

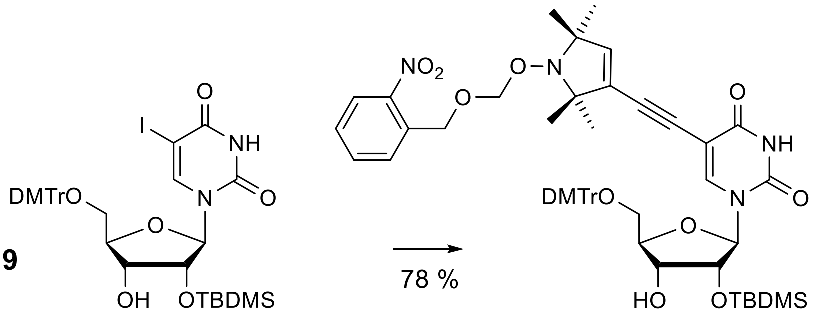

20

To a solution of compound **19** (2.00 g, 2.54 mmol, 1.0 eq.) and alkyne **13** (840 mg, 2.54 mmol, 1.0 eq.) in 25 mL anhydrous dimethylformamide were added triethylamine (2.6 mL, 18.5 mmol, 7.2 eq.), copper(I) iodide (116 mg, 610 µmol, 0.24 eq.) and tetrakis(triphenylphosphine)palladium (352 mg 305 µmol, 0.12 eq.). The reaction mixture was stirred for 21 h at room temperature and then diluted with water and dichloromethane. The aqueous phase was extracted with dichloromethane. The combined organic phase was dried over magnesium sulphate and the solvent was removed under reduced pressure (max. 30 °C water bath temperature). Purification by silica gel chromatography (cyclohexane/acetone 3:1 + 1% triethylamine) yielded **20** as an off white foam (78%, 1.95 g, 1.97 mmol). R_f = 0.29 (cyclohexane/acetone 3:1 + 1% triethylamine). ^{1}H-NMR (400 MHz, DMSO-d_6): 11.78 (bs, 1H, -NH), 8.09 (d, ^3J = 8.2 Hz, nitrophenyl H-3), 8.04 (s, 1H, pyrimidine H), 7.77 (m, 2H, nitrophenyl H-5, H-6), 7.60 – 7.56 (m, 1H, nitrophenyl H-4), 7.41 – 7.39 (m, 2H, trityl-H), 7.29 – 7.25 (m, 6H, trityl-H), 7.20 – 7.16 (m, 1H, trityl-H), 6.86 – 6.83 (m, 4H, trityl-H), 5.80 (d, ^3J = 4.7 Hz, 1H, H-1’), 5.28 (bs, 1H, pyrrole H), 5.13 (d, ^3J = 5.6 Hz, 1H, 3’-OH), 4.99 (s, 2H) and 4.89 (s, 2H, nitrophenyl-CH_2-O- and -O-CH_2-O-), 4.33 (t, ^3J = 4.5 Hz, 1H, H-2’), 4.03 – 4.00 (m, 2H, H-3’, H-4’), 3.72 – 3.70 (m, 6H, -OCH_3), 3.29 – 3.19 (m, 2H, H-5’/5’’), 1.08 – 0.92 (m, 12H, pyrrole CH_3), 0.85 (s, 9H, -$SiC(CH_3)_3$), 0.06 (s, 3H, -$SiCH_3$), 0.05 (s, 3H, -$SiCH_3$) ppm. ^{13}C-NMR (100 MHz, DMSO-d_6): 161.1, 158.1, 149.4, 147.1, 144.6, 142.0, 139.0, 135.33, 135.26, 134.0, 133.8, 130.1, 129.60, 129.55, 128.63, 128.59, 127.9, 127.5, 126.7,

126.4, 124.6, 113.2, 99.7, 98.9, 88.5, 86.9, 86.0, 84.1, 83.4, 75.9, 70.7, 69.6, 68.4, 66.7, 55.0, 39.5, 25.6, 17.9, -4.8, -5.2 ppm. ^{29}Si-NMR (79.5 MHz, DMSO-d$_6$): 21.5 ppm. MS (ESI): *m/z*: 990.77 [M + H$^+$]. HRMS: calcd. for $C_{54}H_{65}N_4O_{12}Si$ [M + H$^+$]: 989.43628; found 989.43555.

Uridine-based phosphoramidite building block **3**

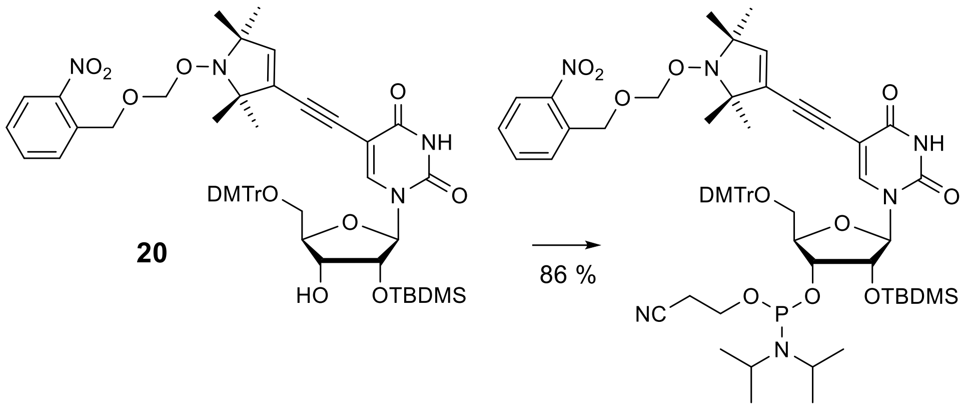

To a solution of compound **20** (1.82 g, 1.84 mmol, 1.0 eq.) in 60 mL anhydrous dichloromethane were added triethylamine (1.60 mL, 9.20 mmol, 5.0 eq.) and *N*,*N*-diisopropylaminocyanoethylphosphor amidic chloride (871 mg, 3.68 mmol, 2.0 eq.) successively. The reaction was stirred over night at room temperature and then quenched with saturated sodium hydrogen carbonate solution. After 10 min the phases were separated and the aqueous phase was extracted with dichloromethane. The organic phase was dried over magnesium sulphate and the solvent removed under reduced pressure (max. 30 °C water bath temperature). Purification by silica gel chromatography (cyclohexane/acetone 1:4) yielded phosphor amidite **3** as a colorless foam (86%, 1.89 g, 1.59 mmol). R_f = 0.21, 0.18 (cyclohexane/acetone 1:4, mixture of two diastereomers). ^{1}H-NMR (400 MHz, DMSO-d$_6$; two diastereomers): 11.76 (bs, 1H, -NH), 8.09 (m, 2H, nitrophenyl H-3 and pyrimidine H), 7.78 (m, 2H, nitrophenyl H-5, H-6), 7.61 – 7.55 (m, 1H, nitrophenyl H-4), 7.42 – 7.38 (m, 2H, trityl-H), 7.30 – 7.26 (m, 6H, trityl-H), 7.21 – 7.17 (m, 1H, trityl-H), 6.86 – 6.83 (m, 4H, trityl-H), 5.92 (d, ^3J = 6.6 Hz) and 5.84 (d, ^3J = 5.8 Hz, together 1H, H-1’), 5.27 (s) and 5.26 (s, together 1H, pyrrole H), 4.99 (s, 2H) and 4.88 (s, 2H, nitrophenyl-CH$_2$-O- and -O-CH$_2$-O-), 4.54 (m,) and 4.49 (t, ^3J = 5.3 Hz, together 1H, H-2’), 4.25 – 4.16 (m, 1H, H-4’), 4.13 – 4.08 (m, 1H, H-3’), 3.84 – 3.74 (m, 2H, -PO-CH$_2$-), 3.70 – 3.69 (m, 6H, -OCH$_3$), 3.55 – 3.50 (m, 2H, isopropyl-H), 3.43 – 3.16 (m, 2H, H-5’), 2.78 – 2.75 (m, 2H, -CH$_2$-CN), 1.11 – 0.92 (m, 24H, 4 pyrrole CH$_3$ and 4 isopropyl CH$_3$), 0.86 (s) and 0.84 (s, together 9H, -SiC(CH$_3$)$_3$), 0.08 and 0.05 and 0.03 (s, together 6H, -SiCH$_3$) ppm. ^{13}C-NMR (100 MHz, DMSO-d$_6$): 177.35, 160.93, 160.80, 158.20, 158.15, 149.47, 147.05, 139.32, 139.22, 135.13, 134.96, 134.87, 133.95, 133.74, 129.63, 129.58, 129.48, 128.61, 128.58, 128.27, 127.90, 127.85, 127.55, 127.54, 127.40, 126.84, 126.29, 126.27, 124.59,

118.66, 113.24, 113.19, 99.67, 99.37, 87.09, 86.97, 86.47, 86.38, 83.70, 70.67, 68.40, 66.66, 64.31, 54.95, 25.50, 25.46, 24.43, 24.37, 24.28, 24.22, 24.17, 19.78, 17.70, 17.63, -4.91, -5.19 ppm. ^{29}Si-NMR (79.5 MHz, DMSO-d_6): 22.67, 21.74 ppm. ^{31}P-NMR (161.9 MHz, DMSO-d_6): 149.55, 148.43 ppm. HRMS: calcd. for $C_{63}H_{81}KN_6O_{13}PSi$ [M + K^+]: 1227.50056; found 1227.49949.

Synthesis, purification and quantification of oligonucleotides

An Expedite Nucleic Acid Synthesis System from PerSeptive Biosystems (Expedite) and an Applied Biosystems Model 392 (ABI) were used for RNA synthesis. For purification by HPLC, a Jasco LC-900 HPLC system equipped with a Jasco UV-975 detector (detection at 254 nm) was used. Reversed phase (RP) HPLC was performed with a preparative Phenomenex Jupiter 4 µm Proteo 90 Å (250 x 10 mm) column. For anion exchange (AE) a semi-preparative BioLC™ DNAPac™ PA-100 (250 x 9 mm) column was used. All RNA samples were concentrated in a SpeedVac (Christ or Savant). Water was treated with DEPC and autoclaved before use. All aqueous stock solutions also contain DEPC treated water.

At both the Expedite Nucleic Acid Synthesis System from PerSeptive Biosystem and the Applied Biosystem Model 392 the standard protocols for a 1 µmol scale were used. Unmodified RNA/DNA phosphor amidites (Bz-A-CE, Ac-C-CE, G(dmf)-CE and U-CE (all 2'-O-TBDMS-protected) as well as dT-CE) were purchased from Link Technologies likewise the CPG solid support for the self-packed columns as well as acetonitrile, which was used as solvent. The terminal alkyne linker **S1** and the Cy5 azide **S2** were obtained from Lumiprobe. 3% Trichloroacetic acid in dichloromethane (deblock solution) was purchased from Sigma for the Expedite and from emp Biotech for the ABI. Acetic anhydride in THF (Cap A), *N*-methyl imidazole in THF/pyridine (Cap B) and iodine in pyridine/H_2O/THF (oxidizer) were both obtained from JT Baker. 0.3 M 5-benzylthio-1-*H*-tetrazole in acetonitrile (activator) was ordered from Link Technologies.

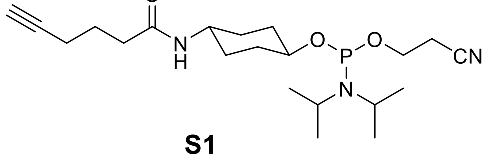

S1

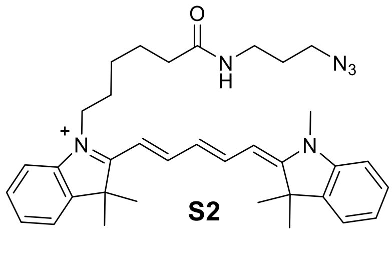

S2

Table S1. RNA strands synthesized in this study.

RNA	Sequence	Modifications
**21**	5’-CGGAUGACCGGUCAUCCG-3’	
**22**	5’-CGGAUGACCGGUCAXCCG-3’	X = protected spin label
**S3**	5’-GGCUGCUUGUCCUXUAAUGGUCCAGXC-3’	X = protected spin label
**26**	5’-YTTTTTTTTTTGGCUGCUUGUCCUUUAAUGGUCCAGUC-3’	Y = terminal alkyne
**S4**	5’-YTTTTTTTTTTGGCUGCUUGUCCUXUAAUGGUCCAGXC-3’	X = protected spin label<br>Y = terminal alkyne

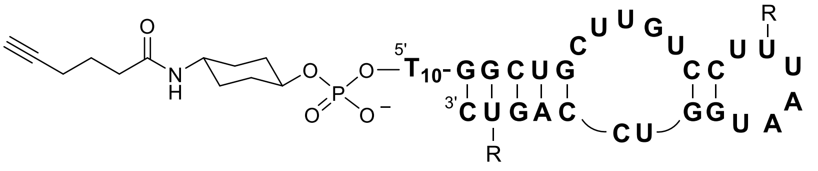

26: R = H **S4**: R = protected TPA **28**: R = free TPA

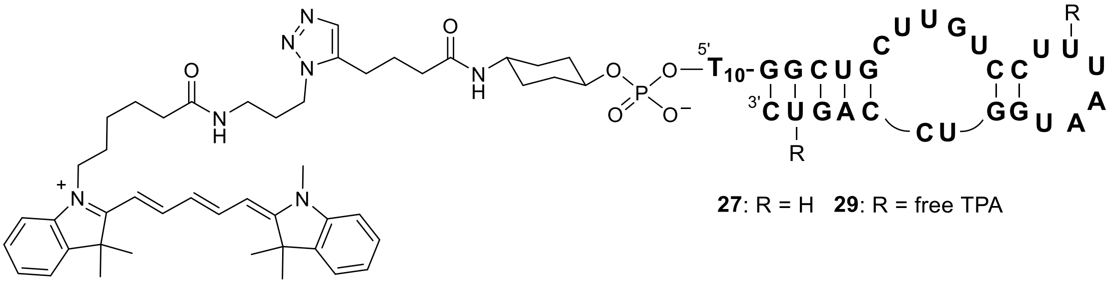

27: R = H **29**: R = free TPA

To isolate the RNAs after strand assembly, they must first be cleaved from the solid support. For this purpose, the CPG was transferred from the column into 2 mL reaction vials and suspended with 2 mL of a mixture of ethanol and 32% ammonia (1:3). The suspension was incubated for 16 h at 37 °C. The supernatant was removed and the CPG washed twice with water. The fractions were combined and evaporated to dryness. For cleavage of the 2’-protecting groups, the residue was treated with 300 µL of a mixture containing *N*-methyl-2-pyrrolidone (NMP), triethylamine (TEA) and triethylamine trihydrofluoride (TEA*3HF) (6:3:4) for 90 min at 65 °C. Then 1.2 mL *n*-butanol was added, and the suspension was stored for 72 h at -20 °C. Afterwards the suspension was centrifugated at 18620 g and 4 °C for 90 min. The supernatant was discarded, and the crude RNA purified by AE-HPLC. Conditions for AE-HPLC: RNA **21** and **22**: A: water, B: 1 M LiCl; gradient: 0 – 70% B within 32 min; flow: 5 mL/min; all other RNAs **S3**-**S5**: A: water, B: 1 M LiCl; gradient: 0 – 80% B within 50 min; flow: 5 mL/min. In all cases the column was heated to 80 °C. RP-HPLC was performed to remove the LiCl from the RNA samples and for further purification. Conditions for RP-HPLC of all RNAs: A: hexafluoroisopropanol (HFIP)/TEA buffer (400 mM HFIP, 16.2 mM TEA, pH 7.8), B: methanol; gradient: 5 – 95% B within 28 min; flow: 4 mL/min. Column temperature: 60 °C. Irradiation of RNAs **22**, **S3**, and **S4** (see below)

deprotected the spinlabels to form RNAs **23**, **25**, and **28**. Finally, RNAs **26** and **28** were converted into the dye-labeled riboswitches **27** and **29** by conjugation with the Cy5 azide **S2** (see below).

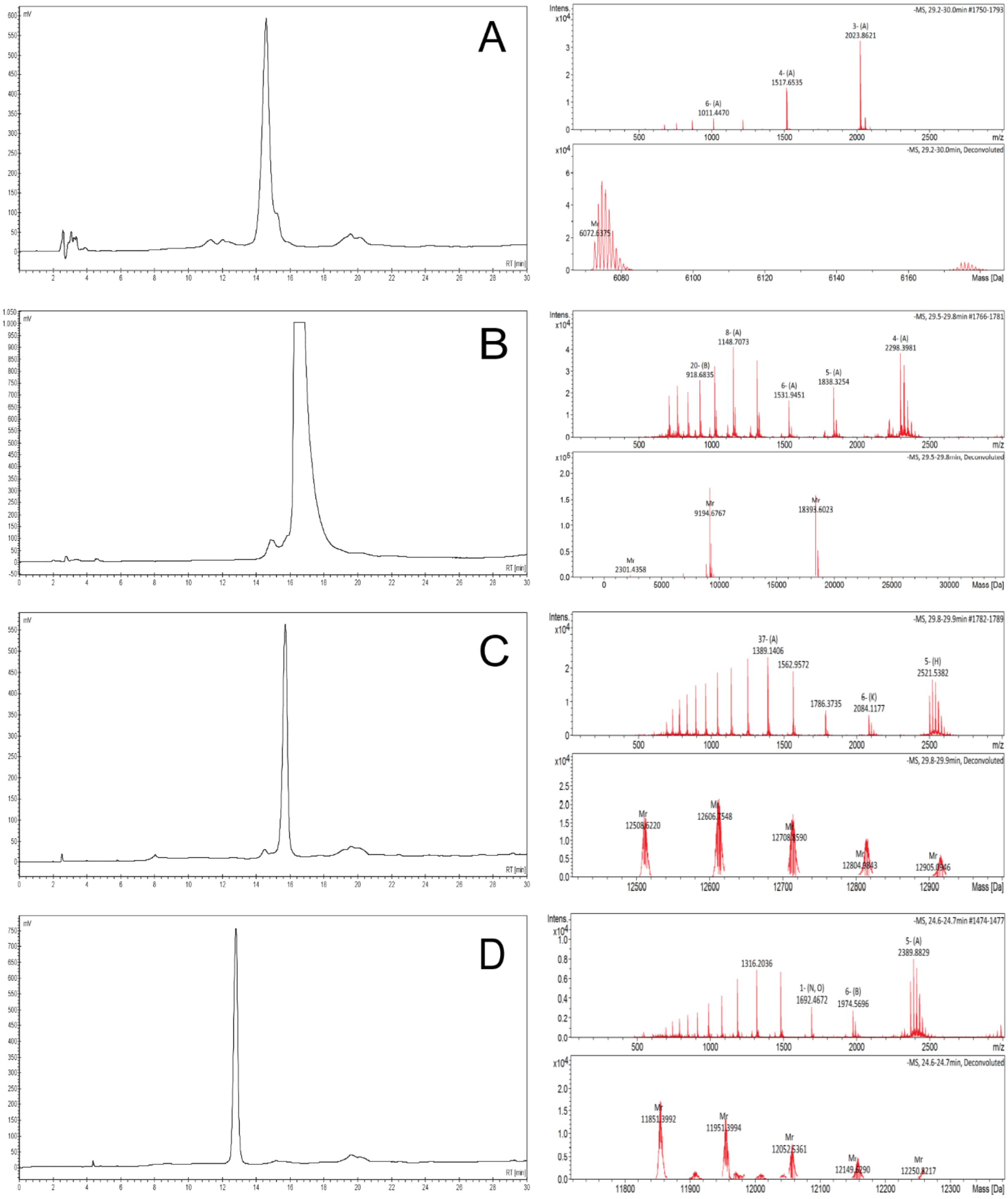

Figure S1. RP-HPLC and mass spectra of RNAs. **A**: 18mer RNA **22** (protected TPA), calculated mass: 6071.9; found: 6072.6; **B**: neomycin aptamer **S3** (2 protected TPA labels), calculated mass: 9192,4; found: 9194.7; **C**: neomycin aptamer **S4** (2 protected TPA labels and alkyne linker), calculated mass: 12503.9; found: 12508.6; **D**: neomycin aptamer **26** (alkyne linker, no TPA label), calculated mass: 11847.6; found: 11851.4). The mass spectra of **S4** and **26** also show signals of Et_3N adducts.

The amount of isolated RNA was determined via UV spectrometry on a Nanodrop 2000c (Thermo Scientific) using Lambert-Beer's law. The extinction coefficients were determined by a nearest neighbour model according to Cantor *et al.*[9]. For the modified nucleotide, identical increments were used as for the unmodified one. The oligonucleotides were analysed by mass spectrometry using a LCMS instrument composed of an Agilent 1200 Series LC system and a microTOF-Q II analyser. HPLC column: XBridge Peptide BEH C18 (300 Å, 3.5 µM, 250 mm x 2.1 mm). HPLC conditions: A: methanol, B: HFIP/TEA buffer (400 mM HFIP, 16.2 mM TEA, pH 7.8); gradient: constant 5% A from 0 – 5 min, 5 – 20% A from 5 – 7 min, 20 – 60% A within 7 – 22 min; flow: 0.25 mL/min. The corresponding HPLC chromatograms and mass spectra are shown in Figure S1.

Photochemical deprotection

Photochemical deprotection of RNA was performed in a round glass cuvette (Carl Roth, 50 mm × 10 mm diameter). A custom-built setup containing three light-emitting diodes was used (Nichia NCCU033, 365 nm, each with 100 mW optical output power)[10]. The NBOM-protected sample was irradiated for 20 min in buffer pH 7.4 (100 mM NaCl, 10 mM NaH_2PO_4/Na_2HPO_4). Afterwards the RNA sample was heated and annealed on a Biometra T-Personal Thermocycler to eliminate the hemiacetal according to the following protocol[4]:

time [min]	temperature [°C]	gradient [°C/s]
	20.0 → 90.0	3.0
70.00	90.0	
	90.0 → 20.0	0.1
1.00	20.0	
	20.0 → 16.0	3.0

After irradiation and annealing, samples were analyzed by RP-HPLC. Conditions: A: HFIP/TEA buffer (400 mM HFIP, 16.2 mM TEA, pH 7.8), B: methanol; gradient: 5 – 95% B within 28 min; flow: 4 mL/min, temperature: 60 °C. No reduced spin labels of the general structure **24** could be detected.

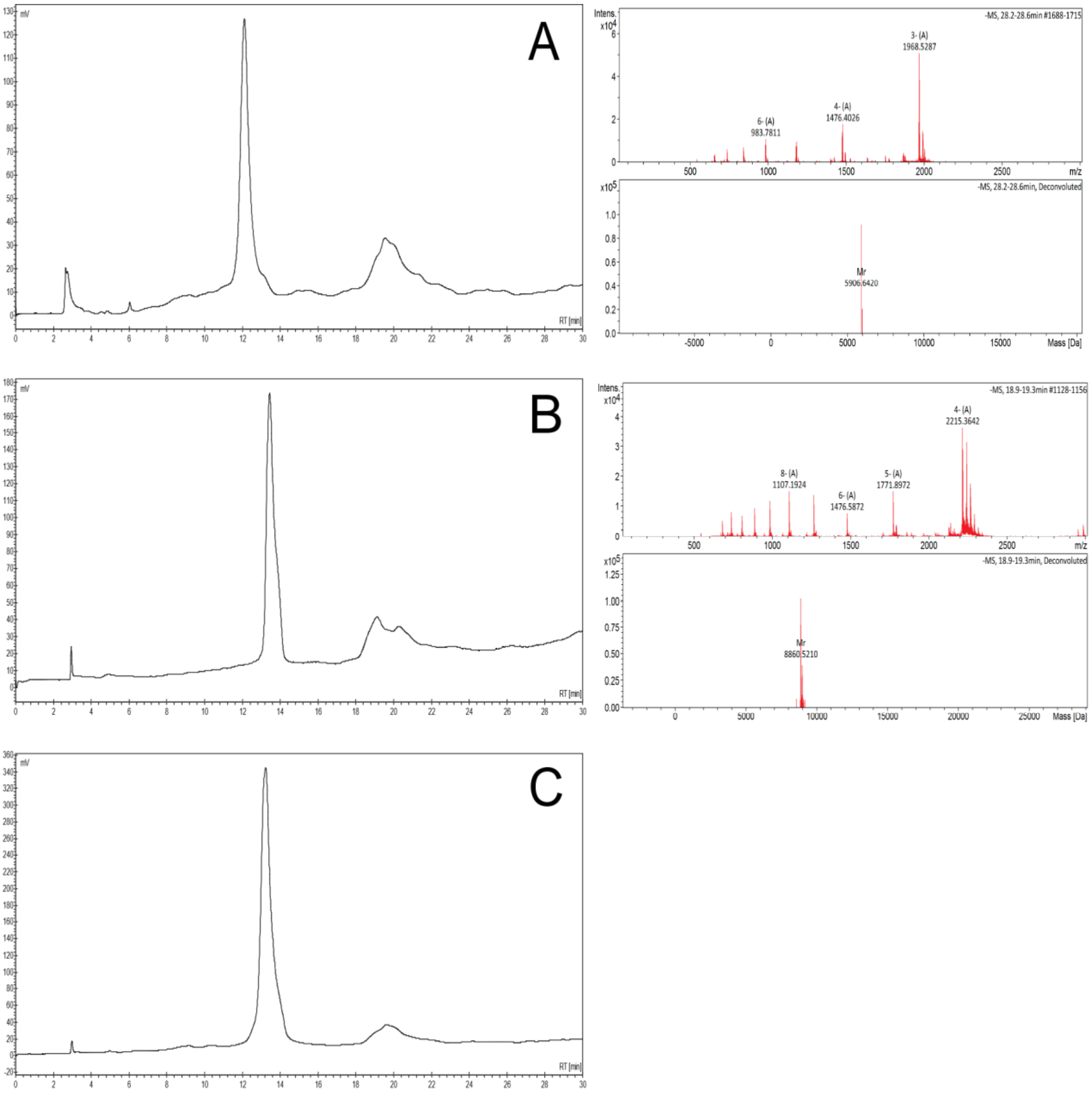

Figure S2. **A**: RP-HPLC chromatogram of the irradiated and annealed spin labeled RNA **23** and the corresponding mass spectrum (calculated: 5905.9; found: 5906.6); **B**: RP-HPLC chromatogram of the irradiated and annealed double labeled neomycin aptamer **25** and the corresponding mass spectrum (calculated: 8860.3; found: 8860.5); **C**: RP-HPLC chromatogram of the irradiated and annealed double labeled neomycin aptamer **28**. After deprotection, RNA **28** was directly converted into **29** by conjugation with the Cy5 reagent **S2** (see below; mass spectrum of **29** see Figure S4). The peaks appearing between 18 to 22 min are caused by the gradient, not by the samples.

Duplex versus hairpin structure of oligonucleotide 23

The monolabeled RNA **23** has a palindromic sequence which can form either hairpin or duplex structures. With the help of the NUPACK tool, the sequence was selected in such a way that, according to the prediction, it does not form any hairpin structures (see Figure S3). To check if the prediction is

correct, a native PAGE gel was performed (12% acrylamide, 7M urea, room temperature, visualization with Sybr gold). For the result see also Figure S3.

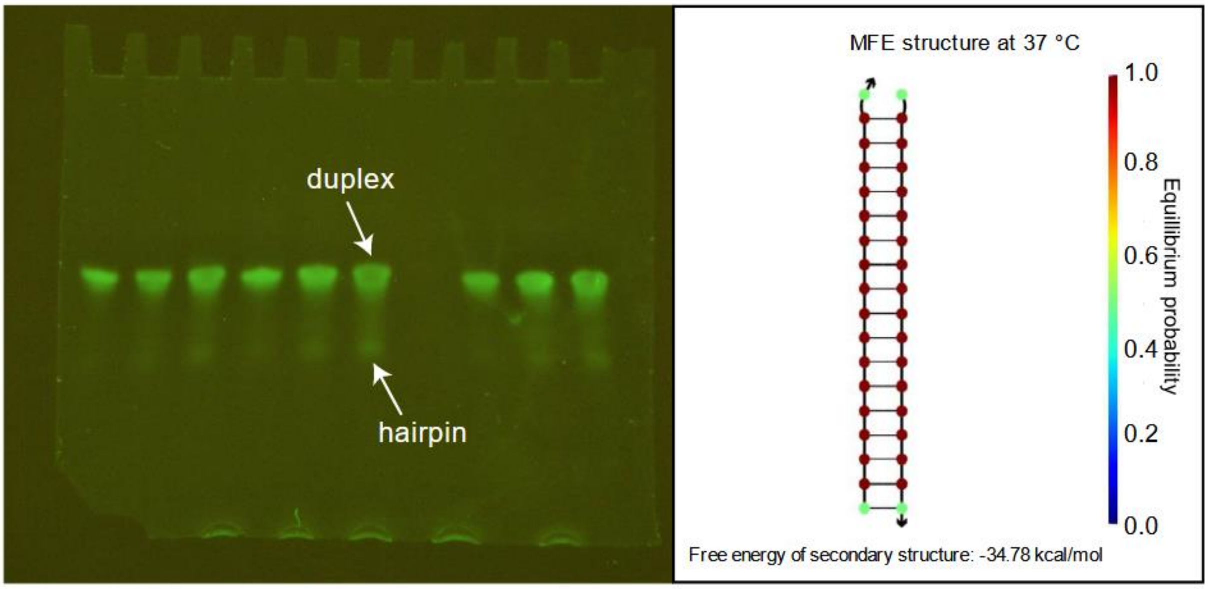

Figure S3. Left: Native PAGE gel of RNA **23**. Palindromic strands in general can form duplexes and monomeric hairpin structures. For RNA **23**, however, the duplex structure by far dominates (slower running spot on the gel). Right: Structure prediction by NUPACK.[11]

Cy5-labeling and inline-probing

To perform in-line probing experiments on the ALFexpress DNA sequencer, a fluorescent Cy5 label must be attached to the RNA. For that purpose, the alkyne modified samples were conjugated with Cy5 azide reagent **S2** via click reaction. Because photochemical deprotection of the spin labels would bleach out the Cy5 dye, this conjugation step was performed afterwards. The following stock solutions were used to achieve the indicated final concentrations:

Reagents	Final concentration	Stock solution
Oligonucleotide	20 µM	Varied
DMSO	50 vol%	—
Cu-TBTA-complex	0.5 mM	10 mM in DMSO/water (55:45)
Azide	30 µM	10 mM in DMSO
Ascorbic acid	0.5 mM	5 mM

First the modified oligonucleotides **26** and **28** were dissolved in water in an Eppendorf vial. Then 1 M triethylammonium acetate buffer pH = 7.0 was added to a final concentration of 0.2 M. DMSO, azide **S2** and ascorbic acid were added one after the other. The solution was homogenised after each addition and finally the air was displaced from the Eppendorf reaction vessel with argon. Cu-TBTA was added, and air was displaced with argon again. The reaction mixture remained at room temperature for 72 h. After addition of 20 µL 3 M sodium acetate buffer, pH = 6.9, and 1 mL ethanol, the components were mixed and stored at -20 °C for 60 min. Afterwards the suspension was centrifuged at 18620 g and 4 °C for 90 min, the supernatant was discarded. The blue pellet was washed with 1 mL ethanol, centrifuged at 18620 g and 4 °C for 10 min, the supernatant was discarded again. The clicked RNA samples were purified using a 16% DPAGE gel.

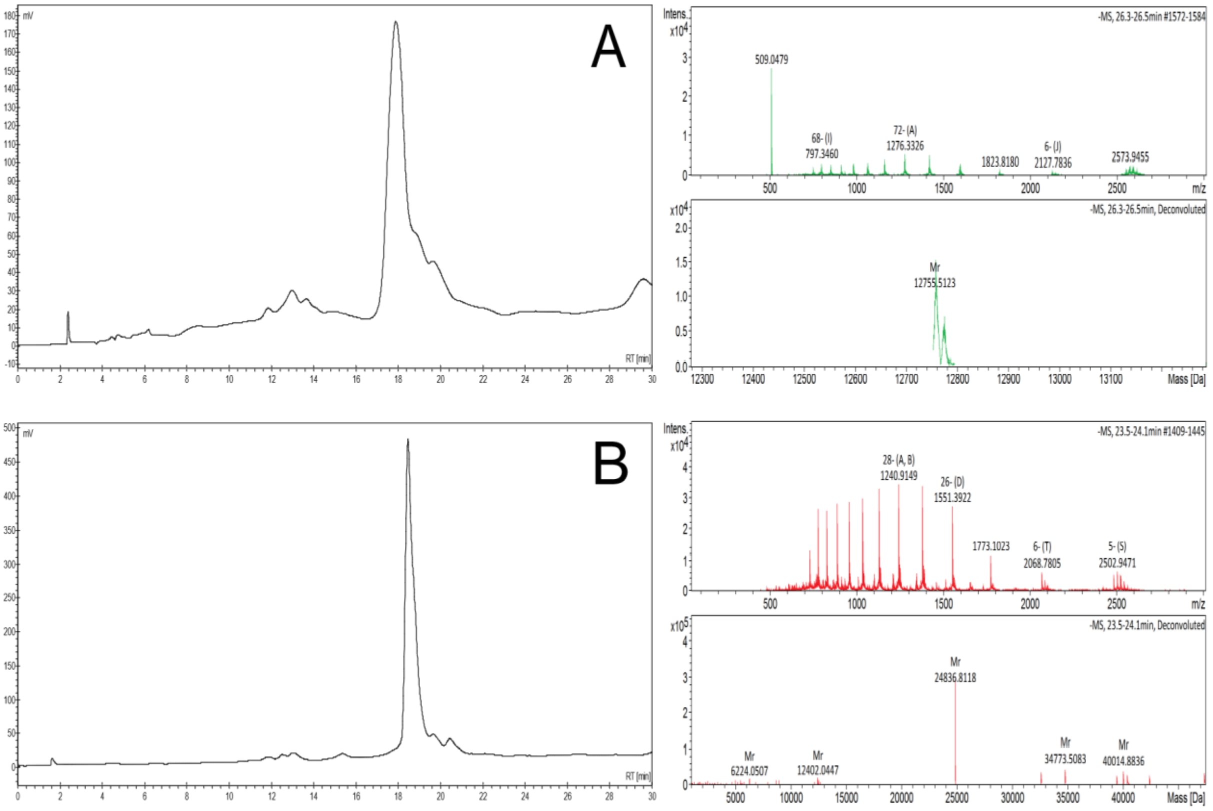

Figure S4. **A**: RP-HPLC chromatogram after click reaction of the irradiated and annealed Cy5 and TPA labeled neomycin aptamer **29** and the corresponding mass spectrum (calculated: 12737.2; found: 12755.5 (M+Na$^+$); **B**: RP-HPLC chromatogram after click reaction of the Cy5 labeled unmodified neomycin aptamer **27** and the corresponding mass spectrum which shows in the deconvolution mainly the doubled mass (calculated: 12413.0; found: 24836.8).

After purification, the RNA (final concentration 150 nM) was mixed with in-line probing buffer (50 mM Tris-HCl (pH 8.3), 20 mM $MgCl_2$, 100 mM KCl) in a total volume of 10 µL and then incubated for 20 h at

37 °C or 110 h at 4 °C. In control reactions the in-line probing buffer was replaced by 50 mM Tris-HCl (pH 8.0) buffer, incubation conditions were maintained. Prior to gel electrophoresis one volume of urea loading buffer x2 (8 M urea, 20 mM EDTA (pH 8.0), 0.2 % crocein orange) was added to each sample and 10 µL were loaded on a denaturing PAGE (16 % or 12 % monomer, 7 M urea). Following running conditions on a DNA sequencing device (ALFexpress, Amersham Biosciences) were chosen: 1500 V (maximum), 60 mA (maximum), 30 W (constant), 55 °C, 2 s sampling interval and 450 minutes running time. The resulting reads were mapped against a hydrolysis ladder (generated by the partial hydrolysis of RNAs **27** and **29**).

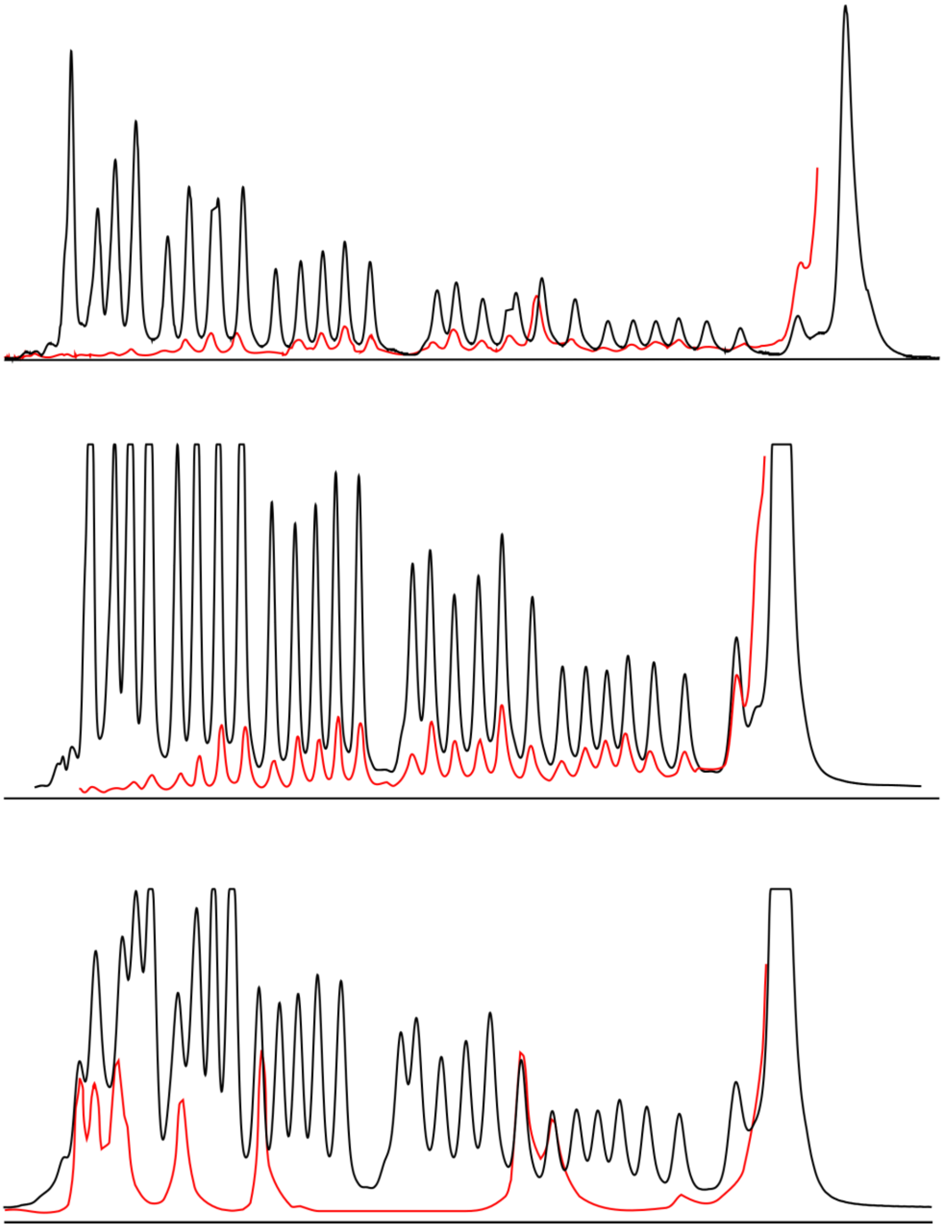

Figure S5. Top: In-line probing of RNA **29** (red curve; 2 TPA labels) at 4 °C. Middle: In-line probing of RNA **29** (red curve) at 37 °C. Bottom: Partial RNAse T1 digestion of RNA **29** (red curve, the peak of G(1) is split in two). The black curve reflects the hydrolysis ladder of the corresponding run.

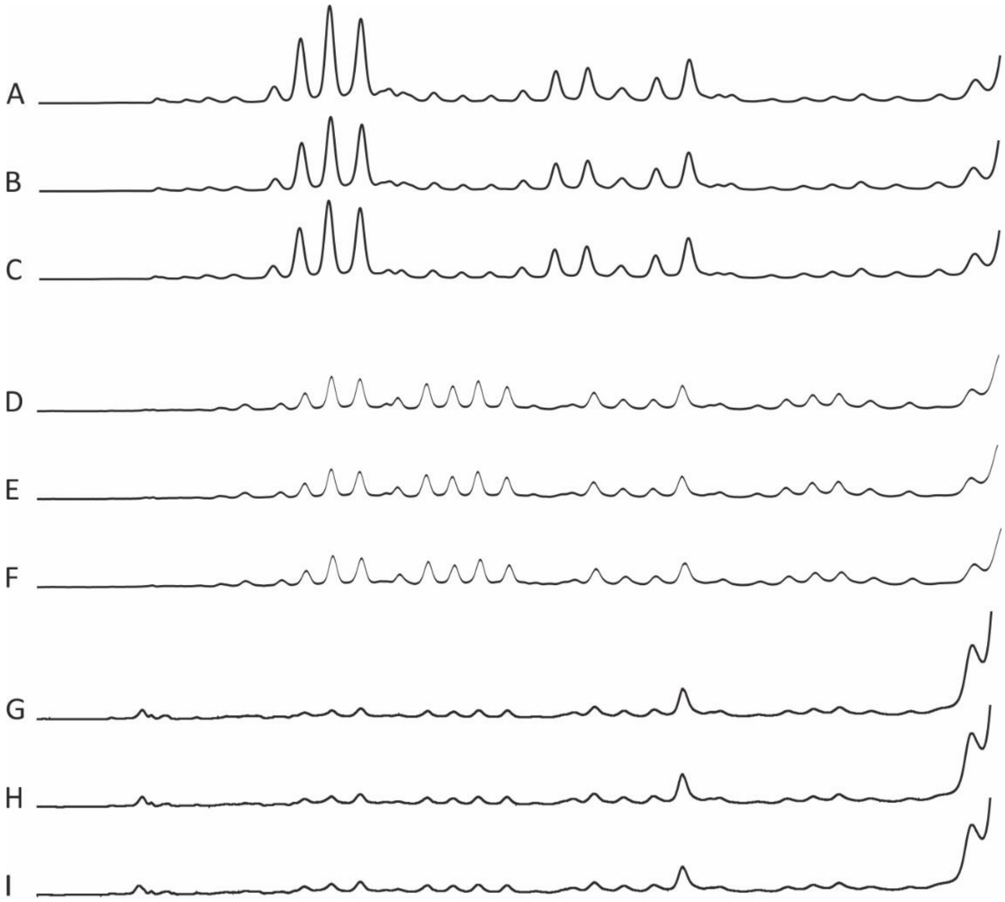

Figure S6. Reproducibility of in-line probing. Three independent repetitions of each experiment are shown. Lanes **A** – **C**: RNA **27** (no spin labels), incubation at 37 °C. Lanes **D** – **F**: RNA **29** (two TPA labels), incubation at 37 °C. Lanes **G** – **I**: RNA **29**, incubation at 4 °C.

EPR measurements

cw-EPR Spectroscopy: After irradiation and annealing of the samples, the continuous wave (cw) EPR spectra were measured at X-Band (9.6 GHz) on a Bruker EMXnano spectrometer equipped with a standard cavity at room temperature with the following experimental parameters: 2.00 mW microwave power, 1.0 G modulation amplitude, 100 KHz modulation frequency, 1.8 ms time constant, sweep time of 20 s and 4 averages.

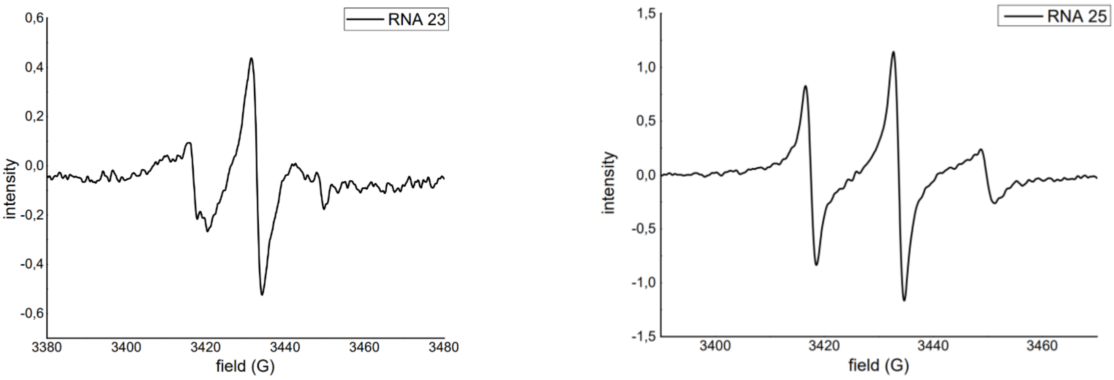

Figure S7. Left: cw-EPR spectrum of RNA **23**. Right: cw-EPR spectrum of RNA **25**.

PELDOR Spectroscopy: After adding 20 % (v/v) deuterated glycerol as a cryoprotectant, 10 µL of the samples were transferred into 1.6 mm outer diameter quartz EPR tubes (Suprasil, Wilmad LabGlass), annealed at the given temperature and frozen in liquid nitrogen. PELDOR experiments were recorded at Q-Band (33.8 GHz) and 50 K on a Bruker ELEXSYS E580 spectrometer equipped with a continuous-flow helium cryostat (CF935, Oxford Instruments), a temperature control system (ITC502, Oxford Instruments) and a 150 W TWT (Applied Systems Engineering Inc.) amplifier using a Bruker EN5107D2 cavity resonator. We used all rectangular pulses and a separate (not phase locked) mw source for the pump pulses. The deadtime-free four-pulse PELDOR sequence [12] was used for all experiments with pulse lengths of 22 ns (RNA **23**) / 32 ns (RNA **25**) for the probe pulses (π/2 and π) and 12 ns for the pump pulse (π). The pump pulse frequency was set to the maximum of the echo-detected field swept spectrum and resonator profile maximum. The frequency of the probe pulses 80 MHz (RNA **23**) 70 MHz (RNA **25**) lower to the pump pulse frequency. To average deuterium modulations, the first inter-pulse delay was increased by 16 ns for eight steps. All time traces where done with a two step phase cycling (alternating π/2 by 180°).

PELDOR data analysis: Each recorded PELDOR time trace was analysed by Tikhonov regularization (not shown) using DeerAnalysis 2022.[13] In addition, each time trace was fitted using the DEERNet (Spinach SVN Rev 5662) method to get a confidence interval of the distance distribution.[14] The DEERNet fit includes the dimensionality of the background, which was usually close to three-dimensional.

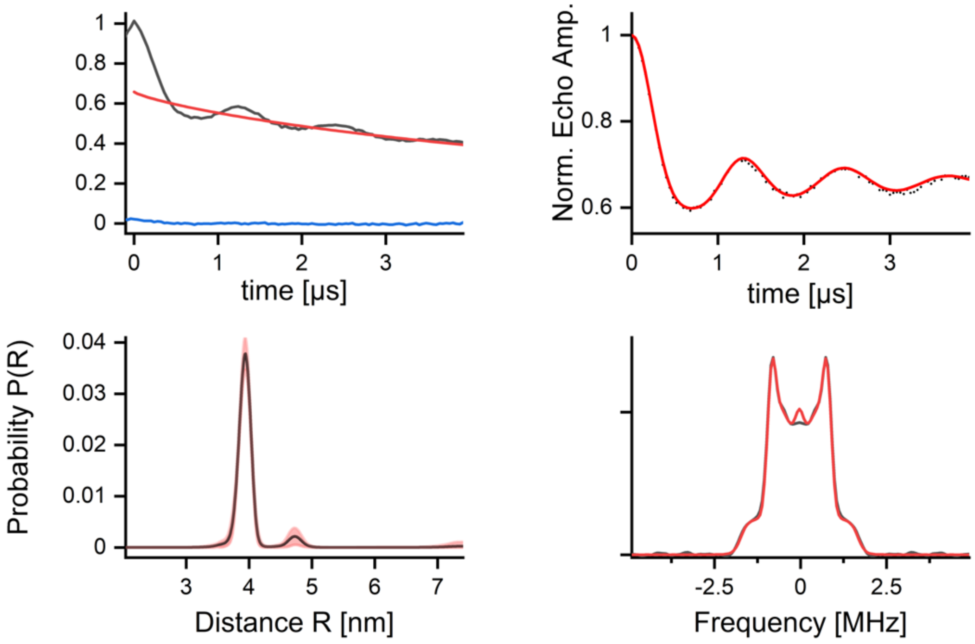

r.m.s. error of fit: 0.013276

Figure S8. **DeerAnalysis results on RNA 23**: Top left normalized PELDOR time trace (black: real, blue: imag part) and background (red). Top right background corrected PELDOR time trace (black dots) and DEERNet fit (red). Bottom left distance probability (black) and 95% confidence band (red shad). Bottom right dipolar peak pattern (black from PELDOR, red DEERNet fit. R.m.s. error estimated by DeerAnalysis. Total measurement time 13h.

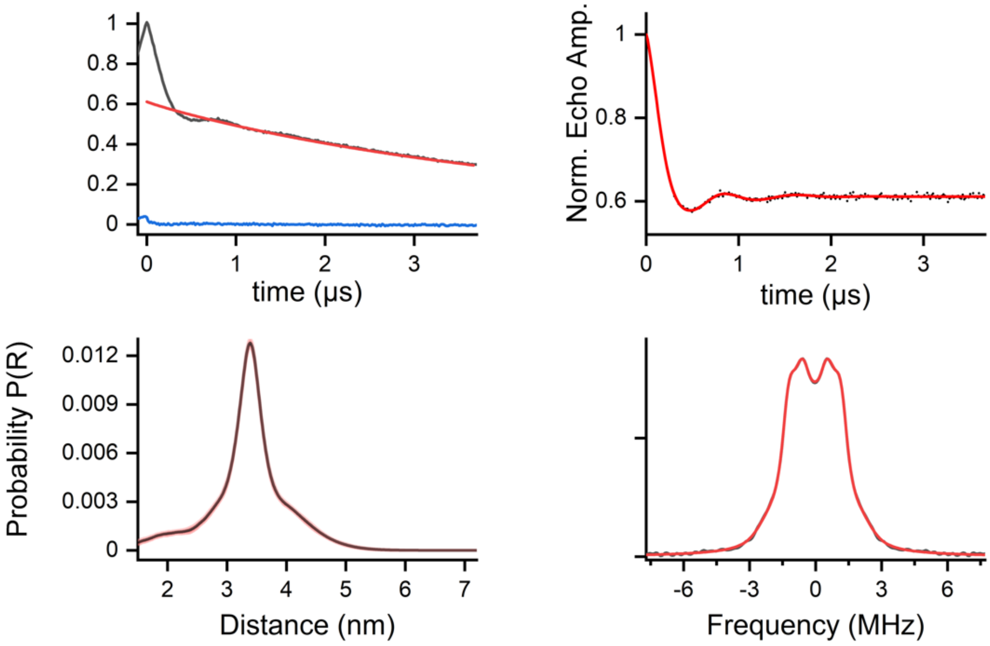

r.m.s. error of fit: 0.006432

Figure S9. DeerAnalysis results on RNA 25: Sample annealed at 0 °C for 20 min before freezing. Top left normalized PELDOR time trace (black: real, blue: imaginary part) and background (red). Top right background-corrected PELDOR time trace (black dots) and DEERNet fit (red). Bottom left distance probability (black) and 95% confidence band (red shadowed area). Bottom right dipolar peak pattern (black from PELDOR, red DEERNet fit). R.m.s. error estimated by DeerAnalysis. Total measurement time 15h.

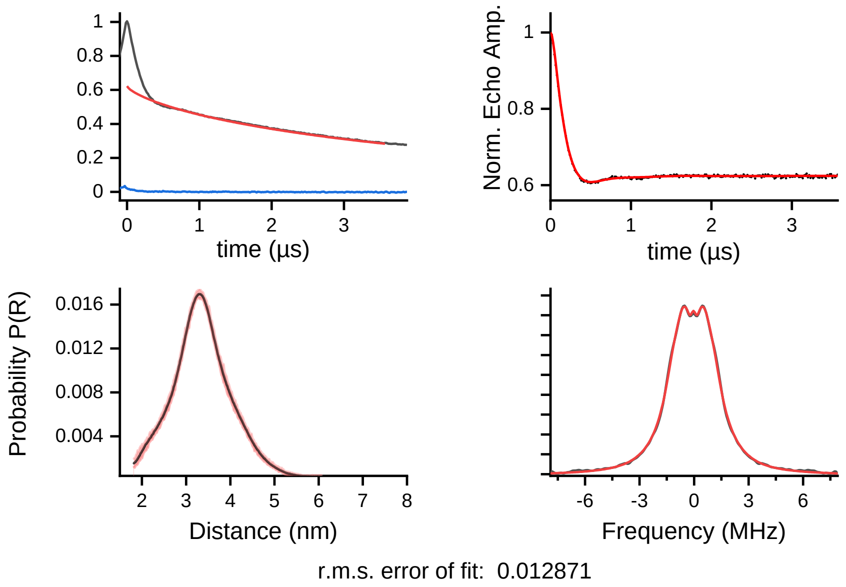

Figure S10a. DeerAnalysis results on RNA 25: Control experiment 1. Sample kept at room temperature before freezing. Compared to Figure S9 the distance distribution is broader but the maximum is almost unchanged. Top left normalized PELDOR time trace (black: real, blue: imaginary part) and background (red). Top right background-corrected PELDOR time trace (black dots) and DEERNet fit (red). Bottom left distance probability (black) and 95% confidence band (red shadowed area). Bottom right dipolar peak pattern (black from PELDOR, red DEERNet fit). r.m.s. error estimated by DeerAnalysis. Total measurement time 1h.

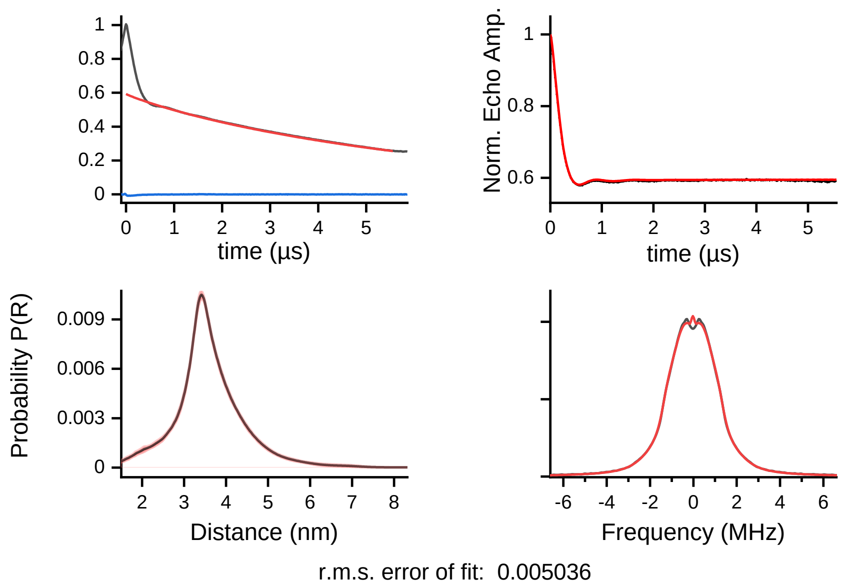

Figure S10b. DeerAnalysis results on RNA 25: Control experiment 2. The sample was kept at -20°C for 8 days before freezing. However, no significant change resulted from the extended annealing period (compare with Figure S9). Top left normalized PELDOR time trace (black: real, blue: imaginary part) and background (red). Top right background-corrected PELDOR time trace (black dots) and DEERNet fit (red). Bottom left distance probability (black) and 95% confidence band (red shadowed area). Bottom right dipolar peak pattern (black from PELDOR, red DEERNet fit). r.m.s. error estimated by DeerAnalysis. Total measurement time 13h.

Determination of duplex stabilities

Melting curves were measured on a JASCO V-650 UV-VIS double-beam spectrometer. 1 mL samples were prepared with a 1 µM concentration in buffer (100 mM NaCl, 10 mM Na_2HPO_4/NaH_2PO_4, pH 7.4). Changes in absorption were detected at 260 nm. A temperature gradient of 1 °C per minute was used. To avoid hysteresis effects, melting temperatures were calculated by fitting at least five independent heating and cooling cycles to a sigmoidal function (DoseResp). The experimental error was determined using the standard deviation with the individual heating and cooling cycles. The graphs used to calculate the melting points are shown. Ten equivalents of neomycin B relative to RNA have been used.

	**26**	**26** + neomycin	**28**	**28** + neomycin	18mer **21**	18mer **23**
Heating cycle 1	52.2 °C	68.4 °C	49.6 °C	63.4 °C	83.6 °C	82.6 °C
Heating cycle 2	52.3 °C	66.2 °C	49.3 °C	64.1 °C	84.4 °C	83.1 °C
Heating cycle 3	52.4 °C	66.5 °C	49.5 °C	63.7 °C	83.6 °C	82.2 °C
Heating cycle 4	52.3 °C	67.5 °C	49.5 °C	63.7 °C	84.2 °C	83.4 °C
Heating cycle 5	52.3 °C	67.1 °C	49.4°C	63.7 °C	84.2 °C	83.6 °C
Heating cycle 6	52.2 °C			63.0 °C		
Cooling cycle 1	52.2 °C	62.6 °C	43.2 °C	58.7 °C	79.4 °C	77.2 °C
Cooling cycle 2	46.8 °C	61.6 °C	42.5 °C	58.0 °C	78.5 °C	78.3 °C
Cooling cycle 3	46.9 °C	61.6 °C	43.3 °C	57.8 °C	79.1 °C	76.9 °C
Cooling cycle 4	47.0 °C	61.7 °C	43.1 °C	57.8 °C	78.2 °C	76.6 °C
Cooling cycle 5	47.0 °C	62.6 °C	43.1 °C	58.0 °C	77.9 °C	76.2 °C
Cooling cycle 6	47.1 °C			57.8 °C		
average melting point	49.5 °C	64.2 °C	46.0 °C	60.8 °C	81.5 °C	79.8 °C
standard deviation	± 2.8 °C	± 2.8 °C	± 3.4 °C	± 2.9 °C	± 2.9 °C	± 3.2 °C
ΔT_{m1}	+ 14.7 °C		+ 14.8 °C			
ΔT_{m2}			-3.5 °C	-3.4 °C	-1.7 °C	

Table S2. Compilation of individual points of inflection of each cooling or heating cycle as well as the resulting melting point and the standard deviation. ΔT_{m1}: The increase in melting point due to the addition of neomycin B; ΔT_{m2}: The decrease in melting points due to the incorporation of the spin labels.

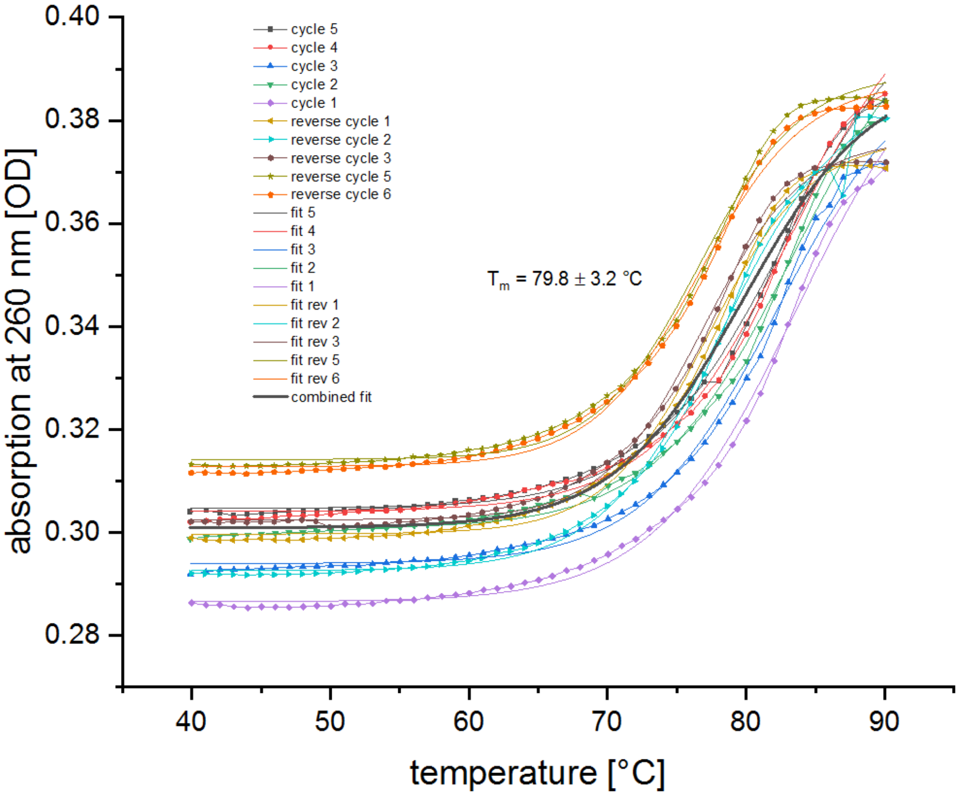

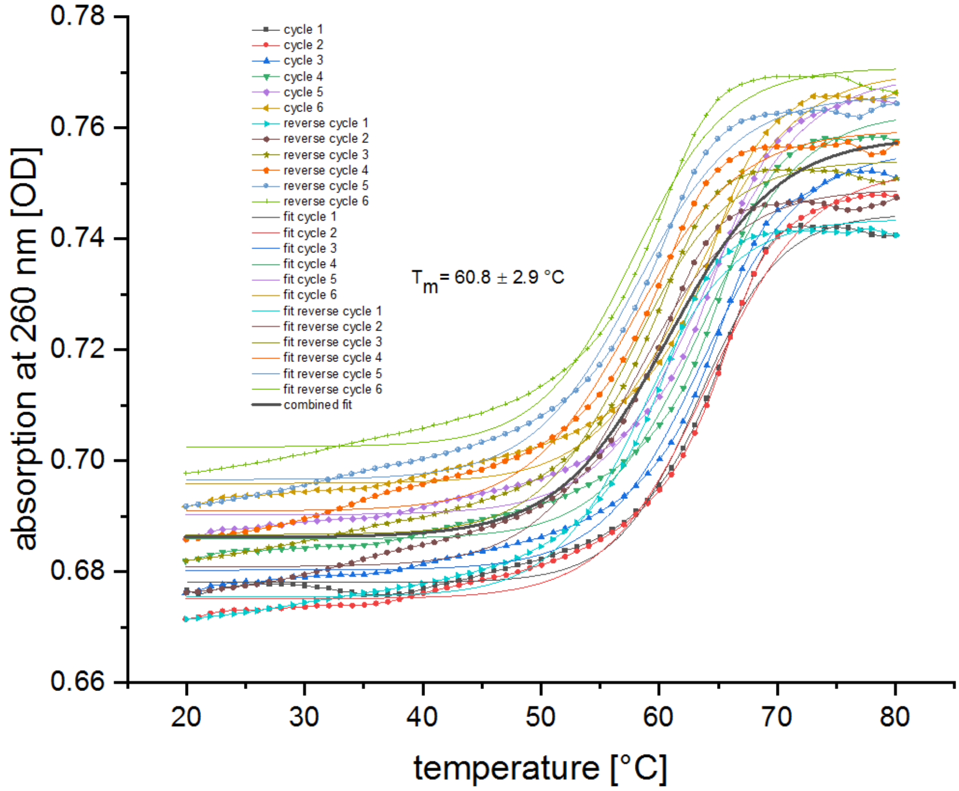

Figure S11. Top: Melting curves of the spin labeled 18mer RNA **23**. Down: Aptamer RNA **28** in the presence of 10 equivalents of neomycin B.

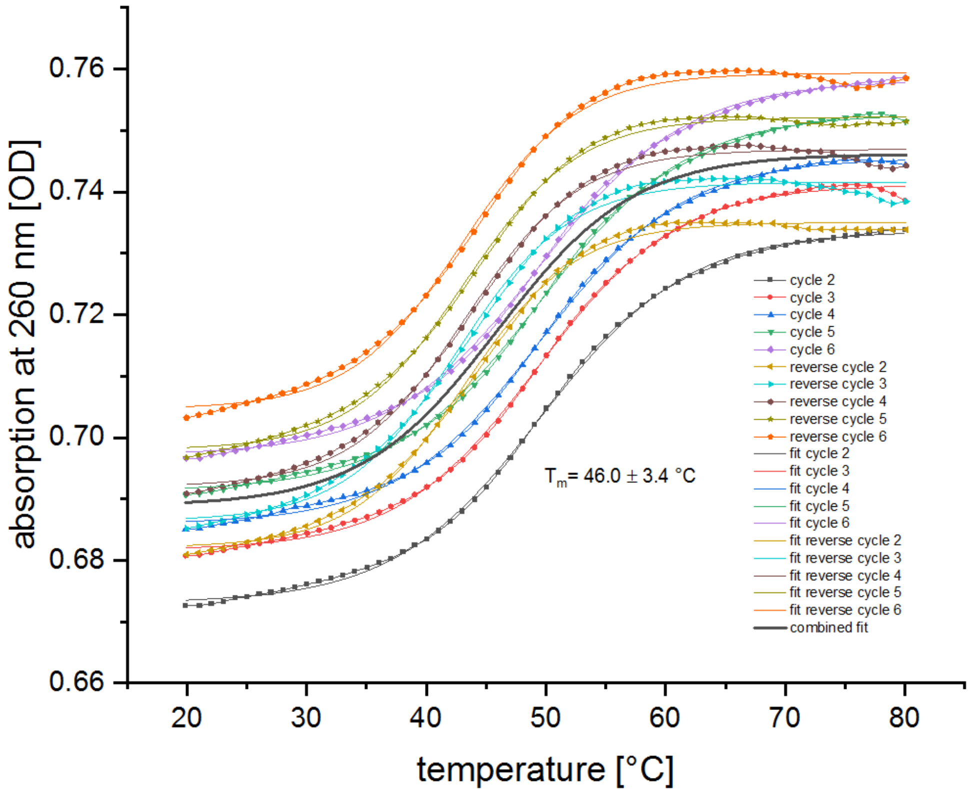

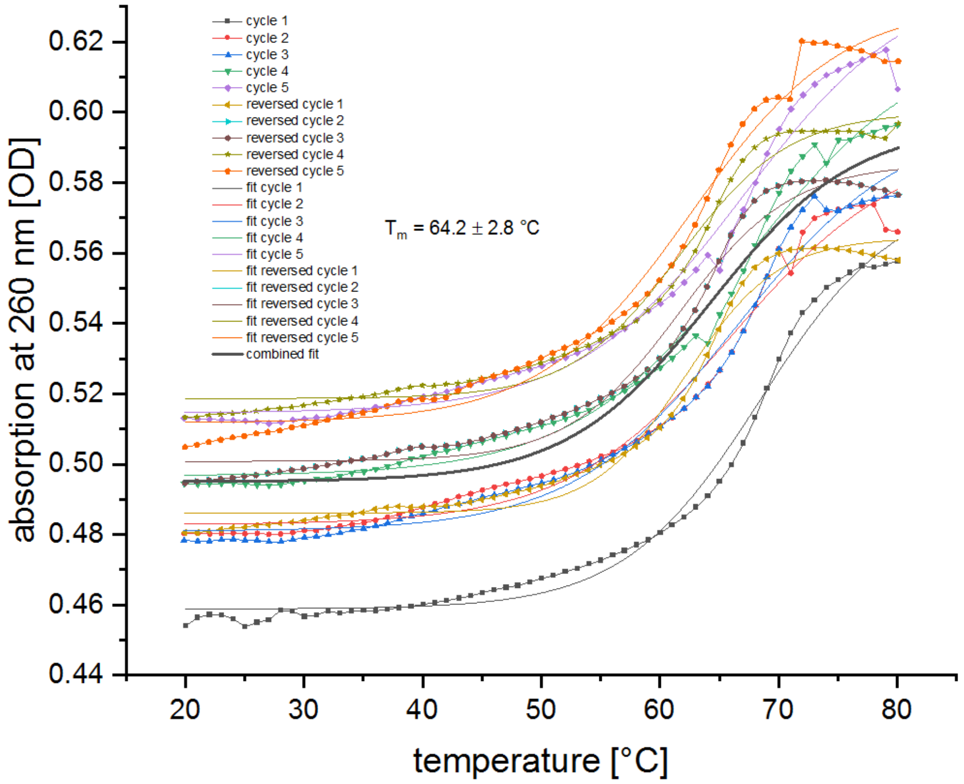

Figure S12. Top: Aptamer RNA **28** in absence of neomycin; Down: Aptamer RNA **26** in the presence of 10 equivalents of neomycin B.

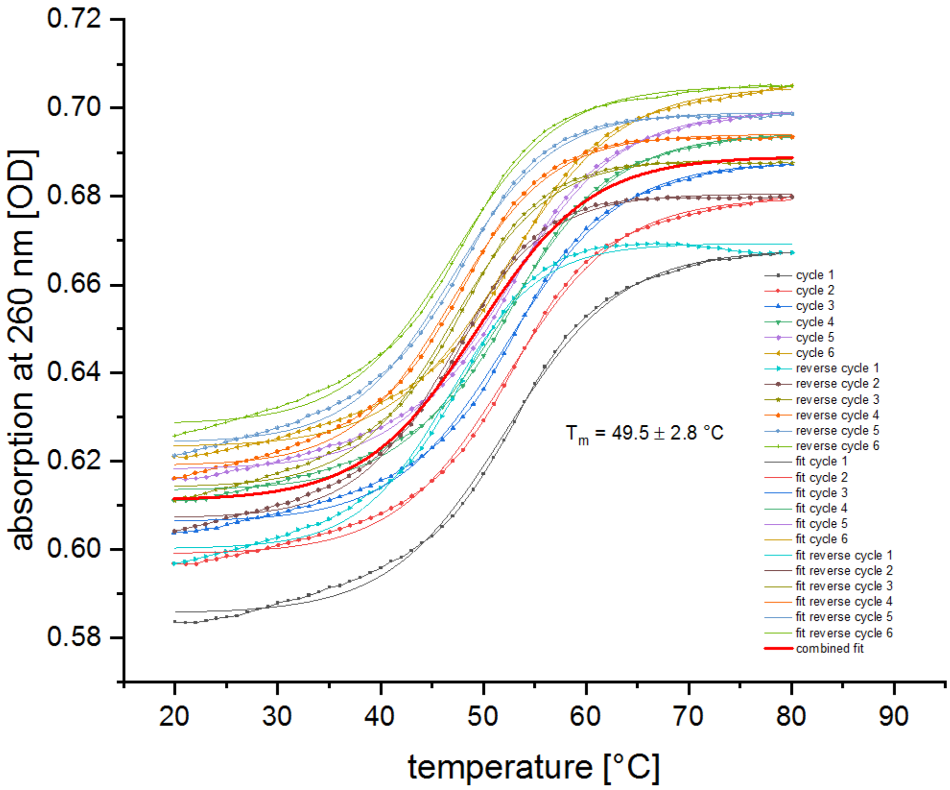

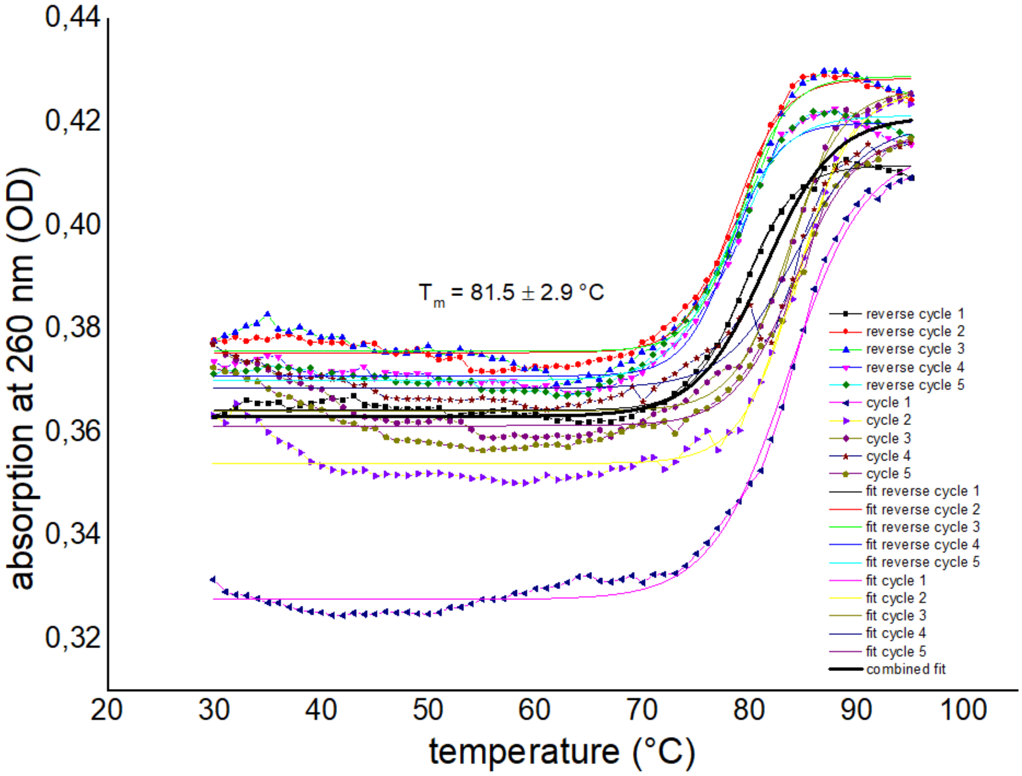

Figure S13. Top: Aptamer RNA **26** in absence of neomycin. Down: 18mer RNA **21**.

References

(1) Wu, H.; Coble, V.; Vasalatiy, O.; Swenson, R. E.; Krishna, M. C.; Mitchell, J. B. An efficient synthesis of 3-(N-piperidinemethyl)-2, 2, 5, 5-tetramethyl-1-oxy-3-pyrroline, a promising radioprotector for cancer radiotherapy. *Tetrahedron Lett.* **2014**, *55* (40), 5570–5571.

(2) Stork, S. W. Facile Synthesis of 3-Formyl-2,2,5,5-tetramethyl-1-oxypyrroline. *Synthesis* **1999**, *8*, 1309–1312.

(3) Yong, P. K.; Banerjee, A. Photochemistry of 2-nitrobenzyl enol ethers: oxidative C=C bond scission. *Org. Lett.* **2005**, *7* (12), 2485–2487.

(4) Weinrich, T.; Jaumann, E. A.; Scheffer, U.; Prisner, T. F.; Göbel, M. W. A Cytidine Phosphoramidite with Protected Nitroxide Spin Label: Synthesis of a Full-Length TAR RNA and Investigation by In-Line Probing and EPR Spectroscopy. *Chem. Eur. J.* **2018**, *24* (23), 6202–6207.

(5) Žemlička, J.; Smrt, J.; Šorm, F. Nucleic acid components and their analogues. XLVIII. Synthesis and structure of nitrogen mustard derivatives of cytidine and 6-azacytidine. *Collect. Czech. Chem. Commun.* **1964**, *29* (3), 635–644.

(6) Asakura, J.; Robins, M. J. Cerium(IV)-mediated halogenation at C-5 of uracil derivatives. *J. Org. Chem.* **1990**, *55* (16), 4928–4933.

(7) Kumar, V.; Malhotra, S. V. Ionic liquid mediated synthesis of 5-halouracil nucleosides: key precursors for potential antiviral drugs. *Nucleosides, Nucleotides & Nucleic acids* **2009**, *28* (9), 821–834.

(8) Meneghesso, S.; Vanderlinden, E.; Stevaert, A.; McGuigan, C.; Balzarini, J.; Naesens, L. Synthesis and biological evaluation of pyrimidine nucleoside monophosphate prodrugs targeted against influenza virus. *Antivir. Res.* **2012**, *94* (1), 35–43.

(9) Cantor, C. R.; Tinoco, I. Absorption and Optical Rotatory Dispersion of Seven Trinucleoside Diphosphates. *J. Mol. Biol.* **1965**, *13* (1), 65–77.

(10) Seven, I.; Weinrich, T.; Gränz, M.; Grünewald, C.; Brüß, S.; Krstić, I.; Prisner, T. F.; Heckel, A.; Göbel, M. W. Photolabile Protecting Groups for Nitroxide Spin Labels. *Eur. J. Org. Chem.* **2014**, (19), 4037–4043.

(11) Zadeh, J. N.; Steenberg, C. D.; Bois, J. S.; Wolfe, B. R.; Pierce, M. B.; Khan, A. R.; Dirks, R. M.; Pierce, N. A. NUPACK: analysis and design of nucleic acid systems. *J. Comput. Chem.* **2011**, *32*, 170–173.

(12) Pannier, M.; Veit, S.; Godt, A.; Jeschke, G.; Spiess, H. W. Dead-Time Free Measurement of Dipole–Dipole Interactions between Electron Spins. *J. Magn. Reson*. **2000**, *142* (2), 331–340.

(13) Jeschke, G.; Chechik, V.; Ionita, P.; Godt, A.; Zimmermann, H.; Banham, J.; Timmel, C. R.; Hilger, D.; Jung, H. DeerAnalysis2006 - a Comprehensive Software Package for Analyzing Pulsed ELDOR Data. *Appl. Magn. Reson.* **2006**, *30* (3–4), 473–498.

(14) Worswick, S. G.; Spencer, J. A.; Jeschke, G.; Kuprov, I. Deep Neural Network Processing of DEER Data. *Sci. Adv.* **2018**, *4* (8), eaat5218.

3,5-dibromo-2,2’,6,6’-tetramethylpiperidin-4-one (**6**)

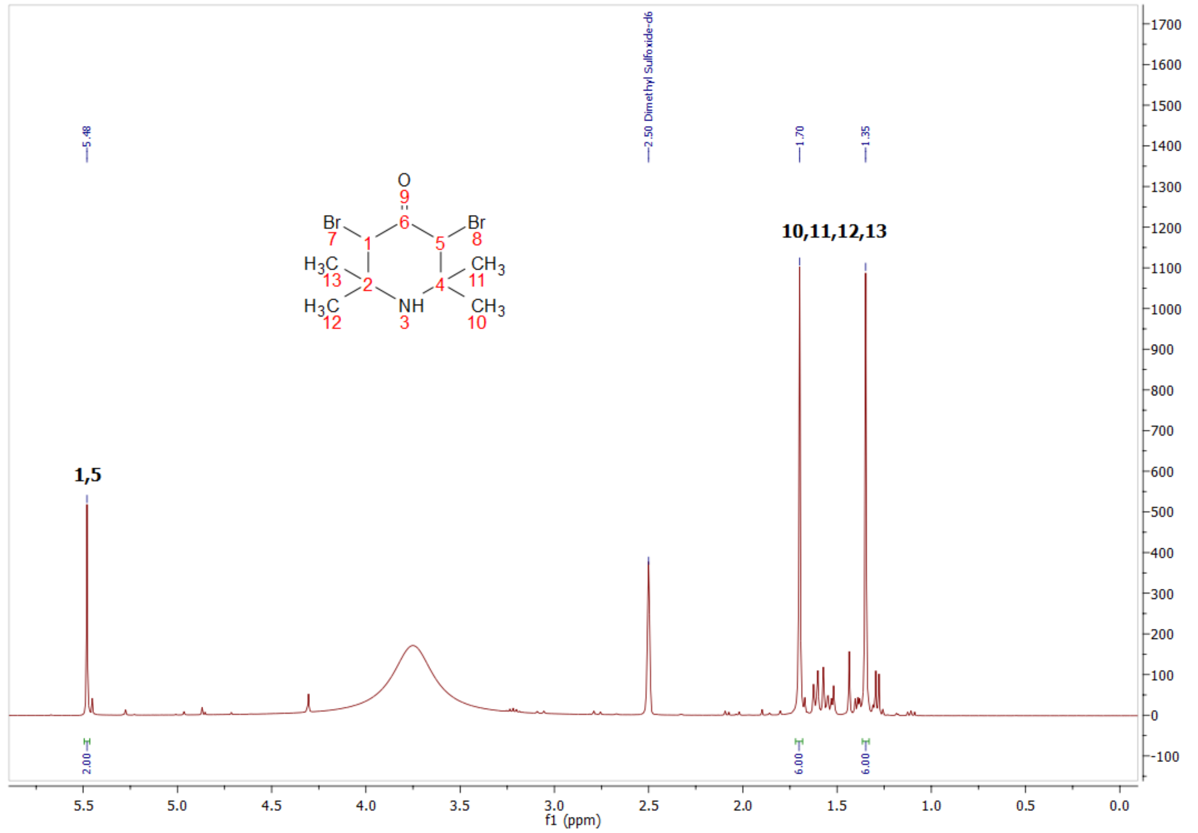

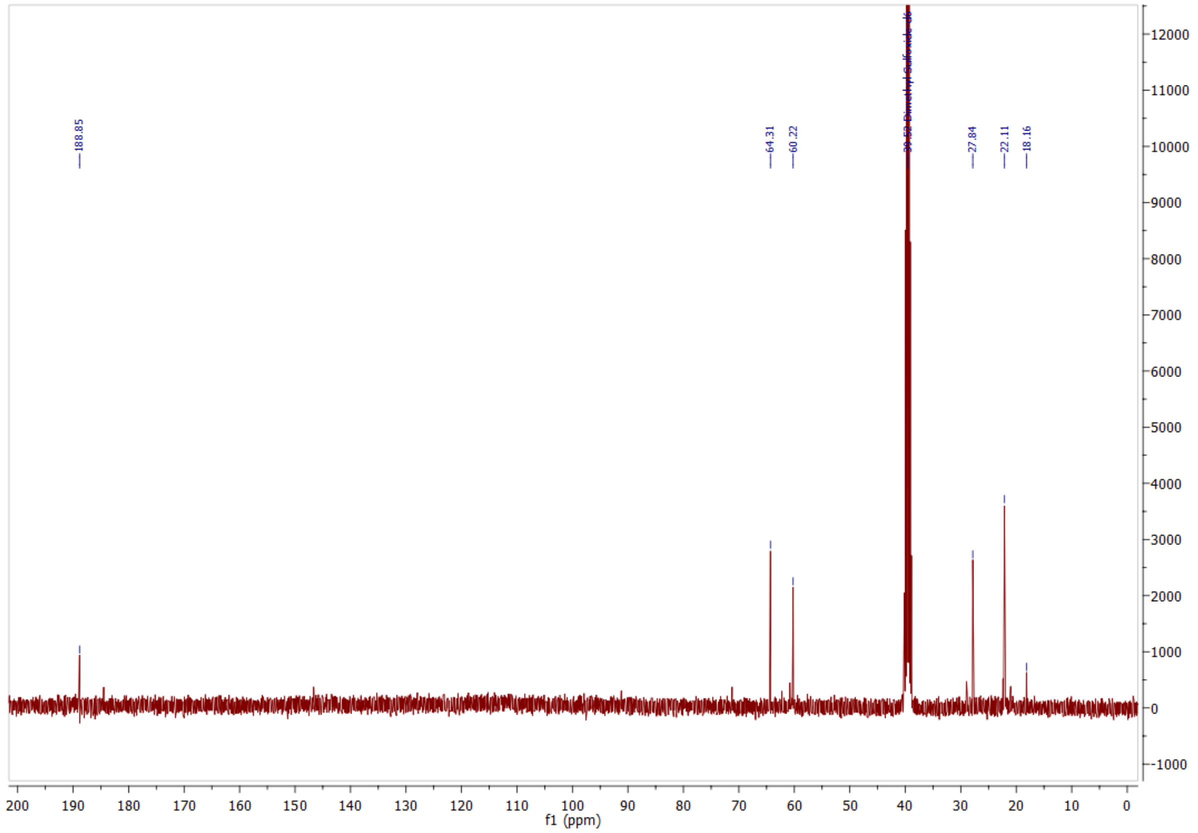

2,5-dihydro-*N*-methoxy-*N*,2,2,5,5-pentamethyl-1*H*-pyrrole-3-carboxamide (**7**)

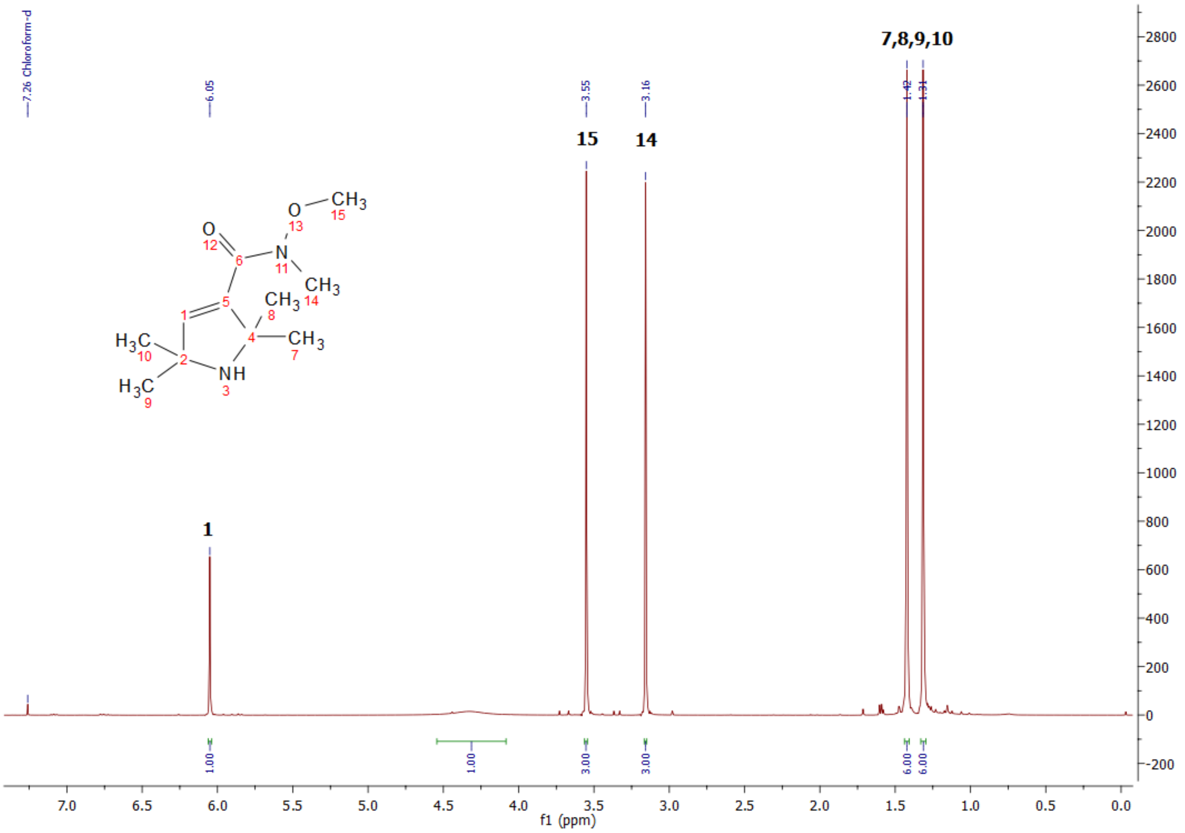

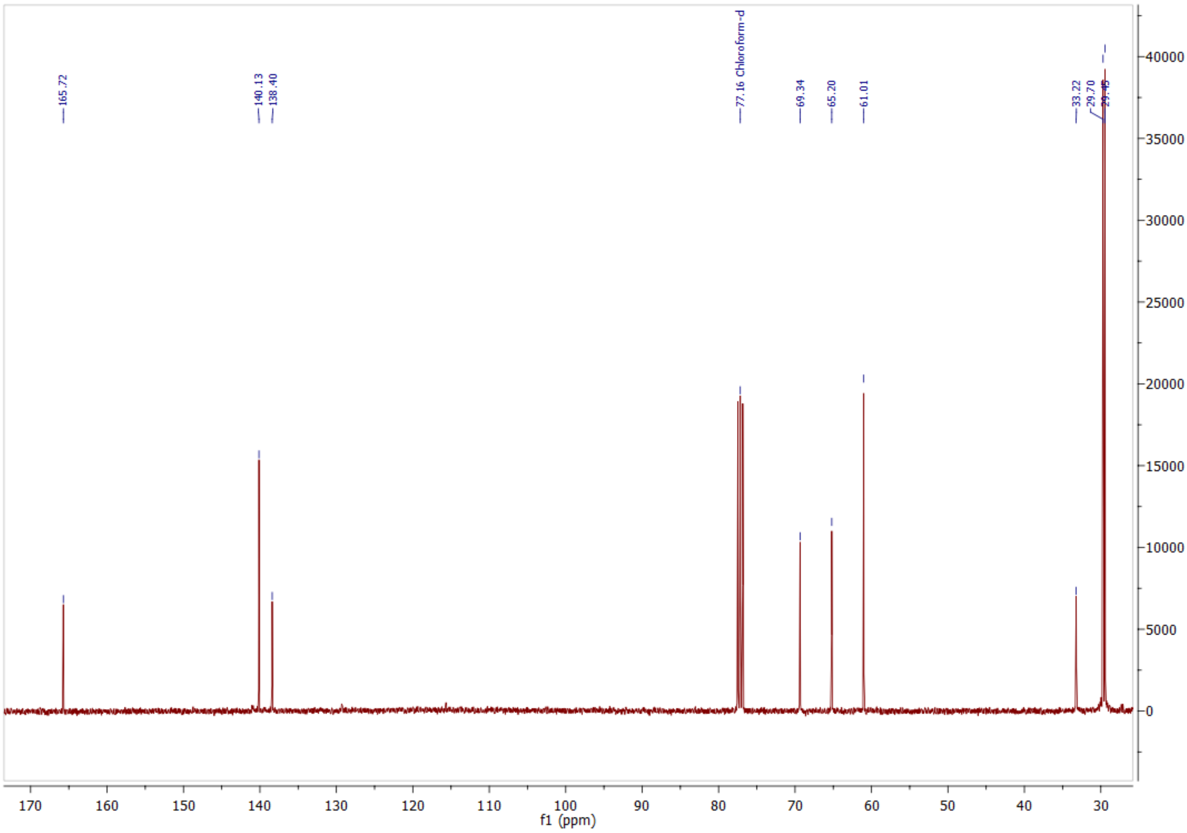

2,5-dihydro-3-[(methoxymethylamino)carbonyl]-2,2,5,5-tetramethyl-1*H*-pyrrol-1-yloxy (**8**)

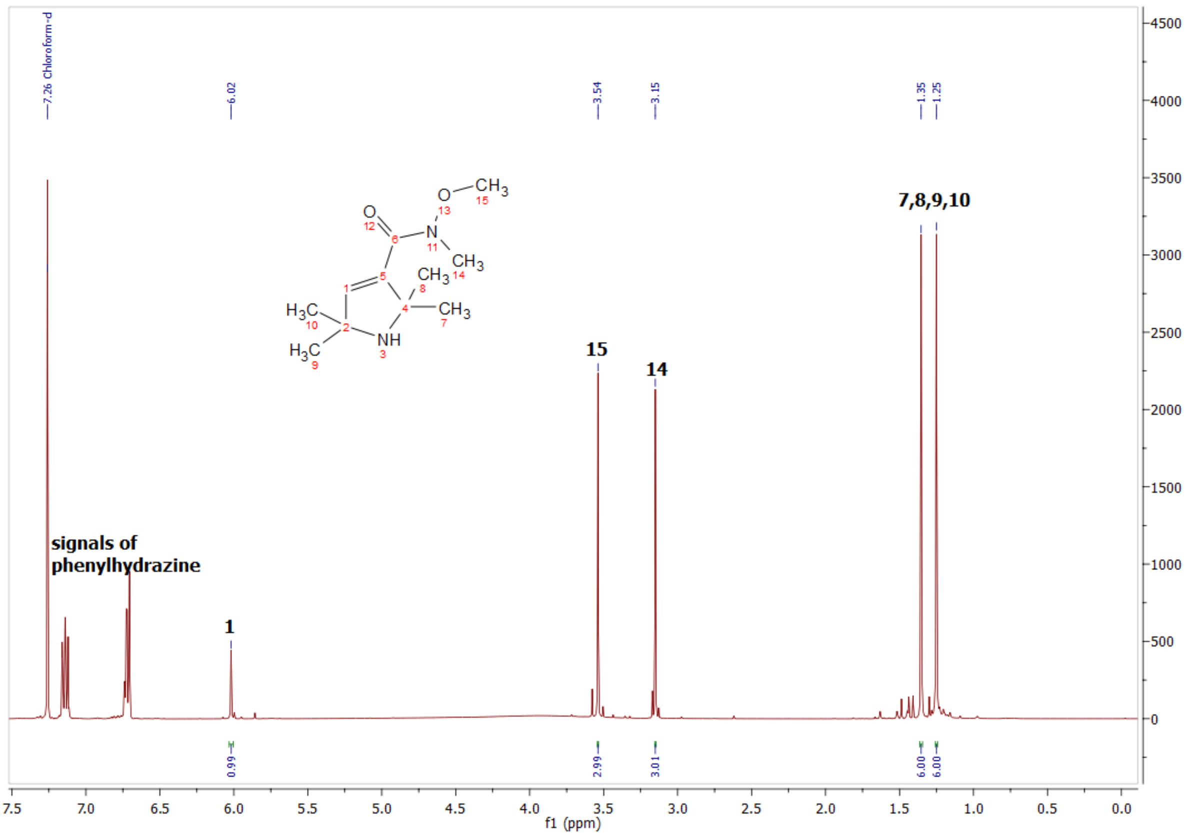

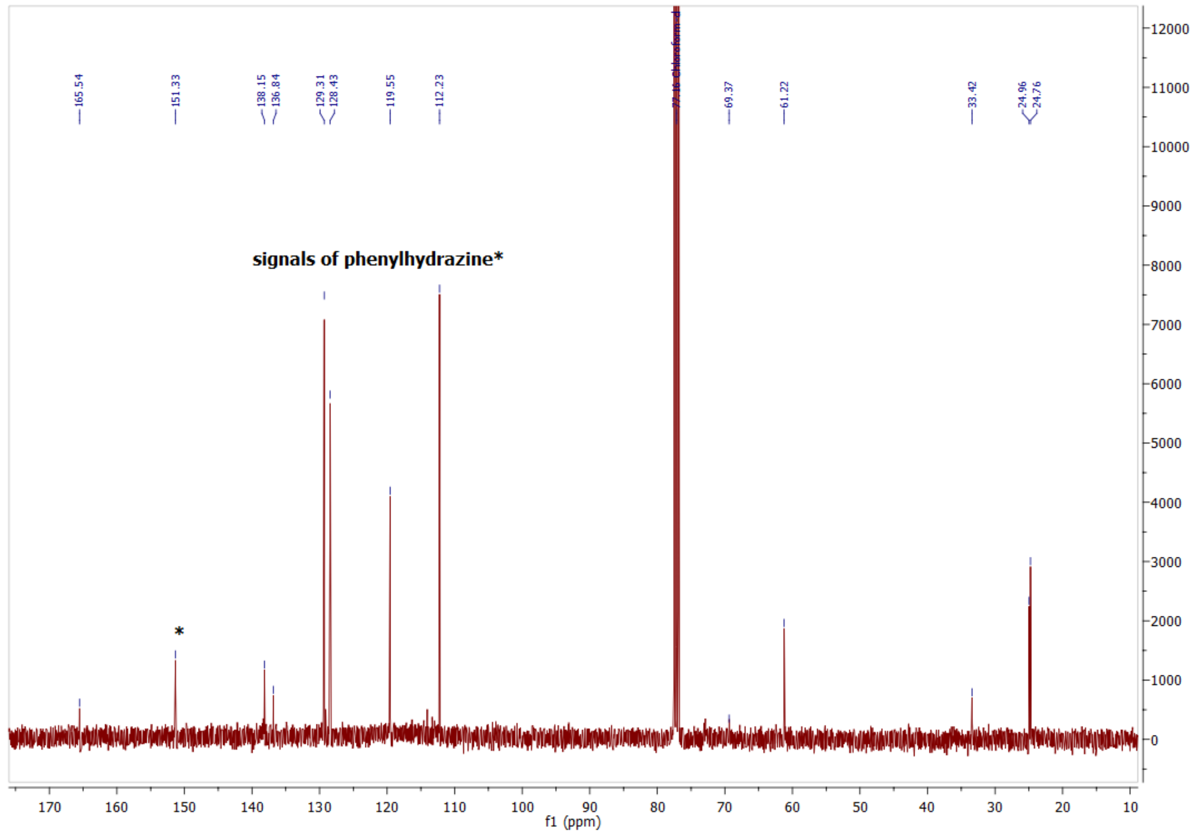

1-[([methylthio]methoxy)methyl]-2-nitrobenzene (**10**)

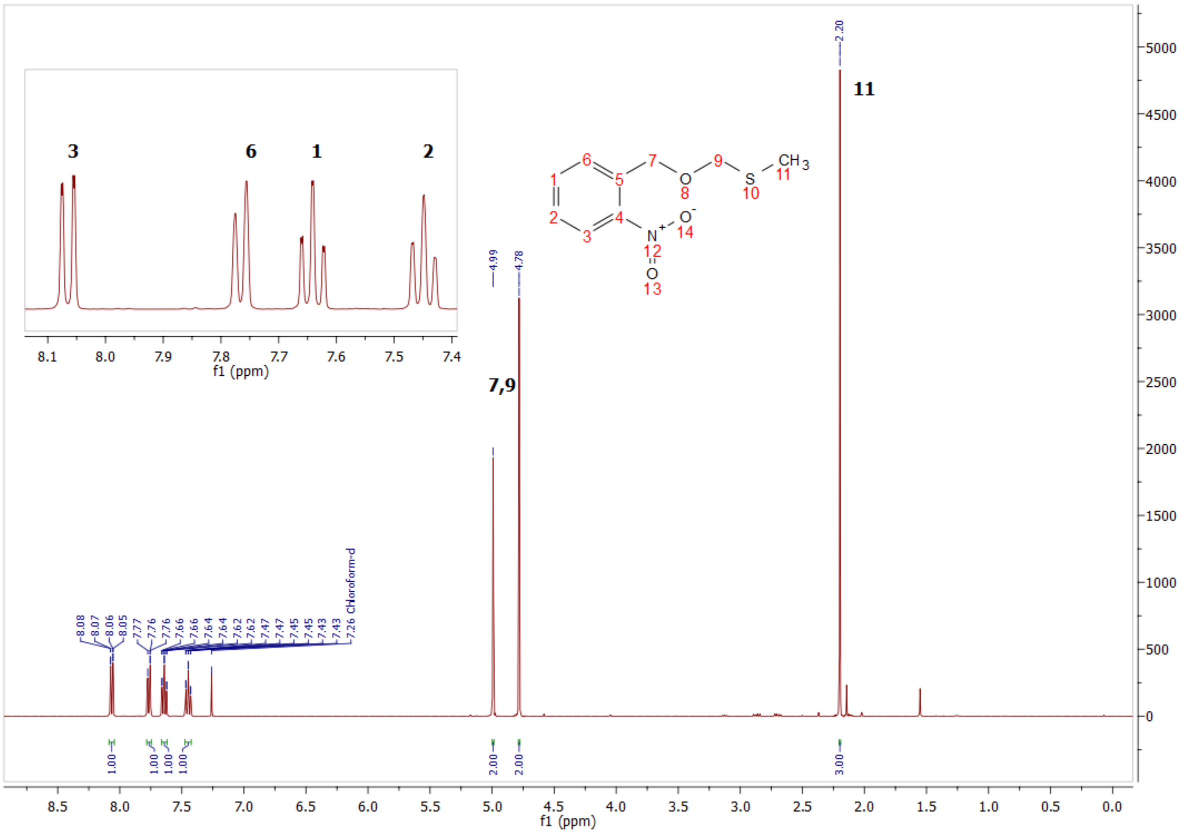

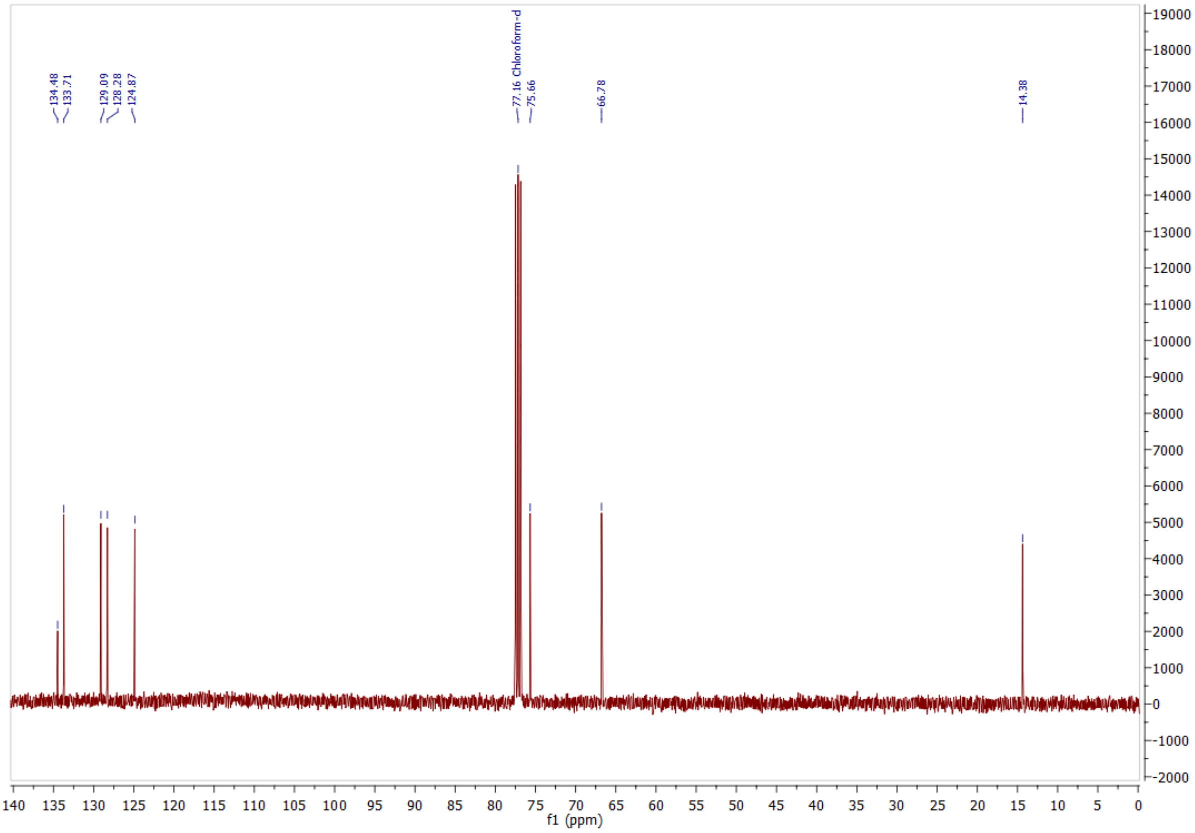

N-methoxy-*N*,2,2,5,5-pentamethyl-1-[([2-nitrobenzyl]oxy) methoxy]-2,5-dihydro-1*H*-pyrrole-3-carboxamide (**11**)

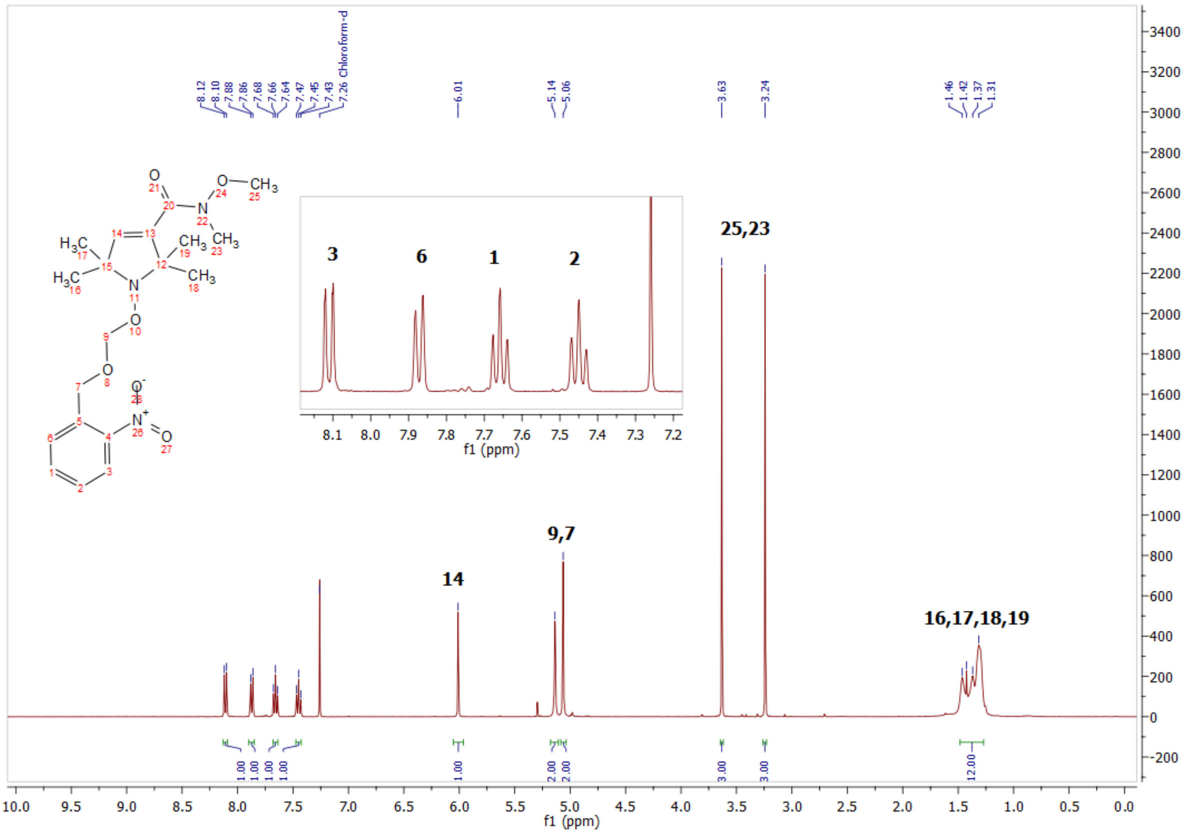

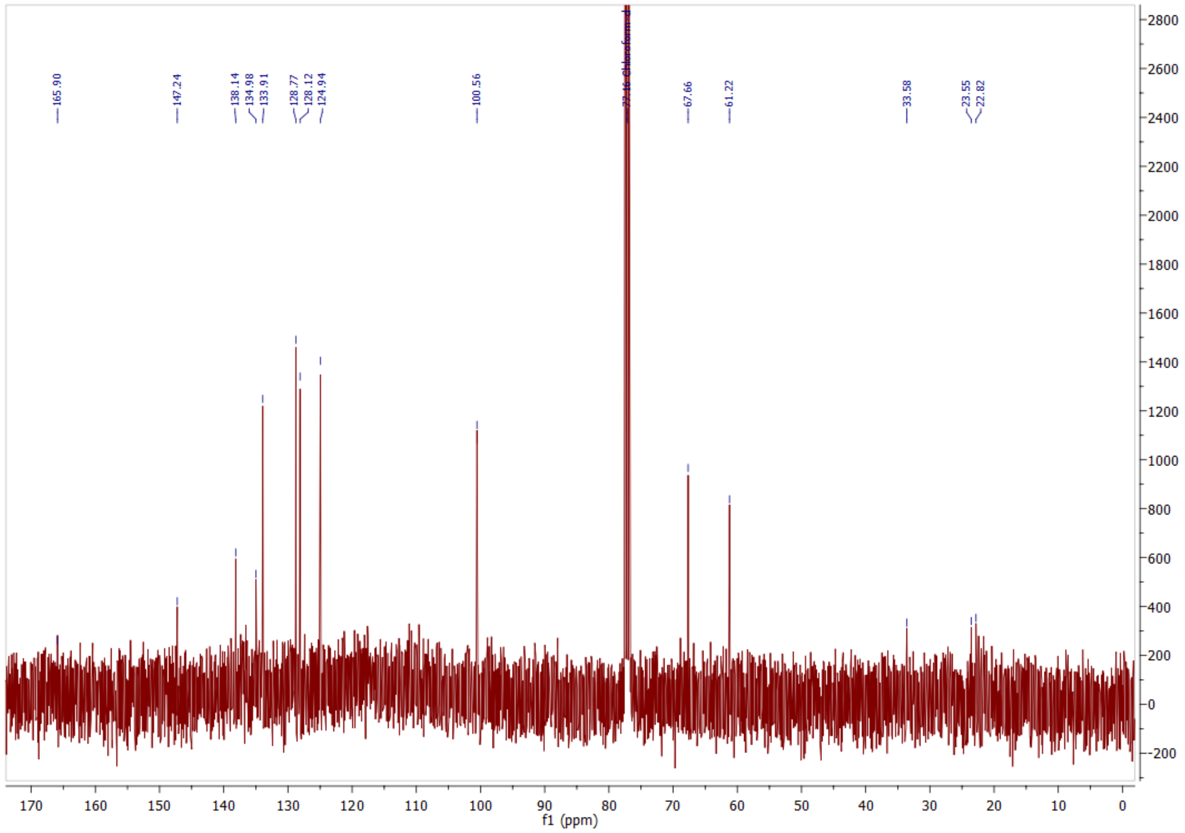

2,2,5,5-tetramethyl-1-[([2-nitrobenzyl]oxy)methoxy]-2,5-dihydro-1*H*-pyrrole-3-carbaldehyde (**12**)

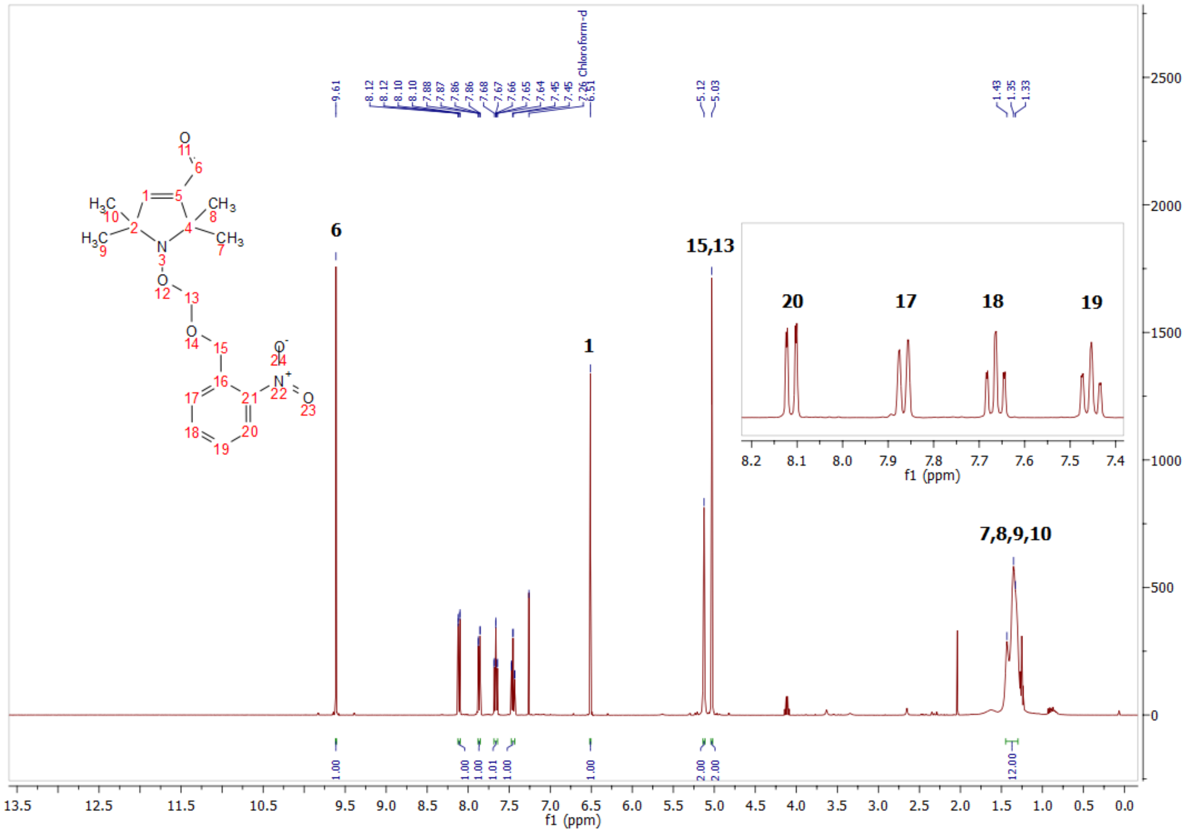

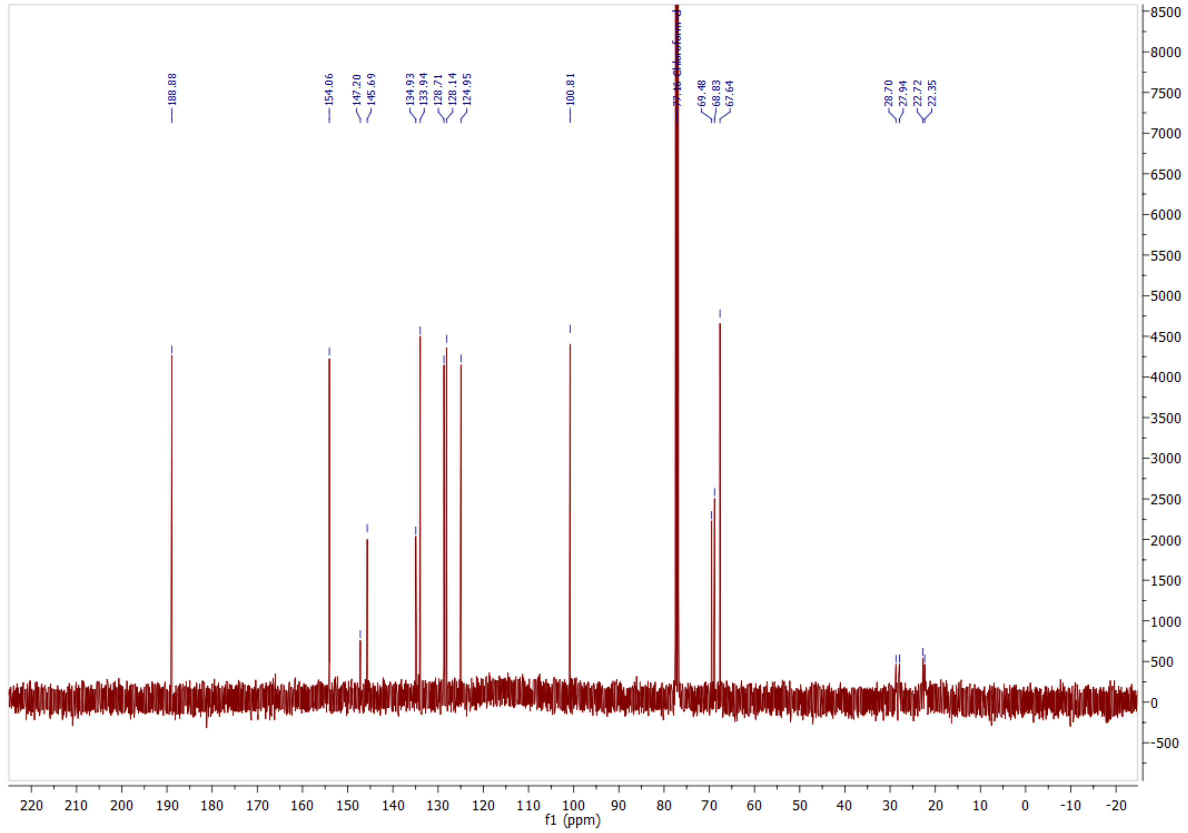

3-ethynyl-2,2,5,5-tetramethyl-1-(((2-nitrobenzyl)oxy)meth oxy)-2,5-dihydro-1*H*-pyrrole (**13**)

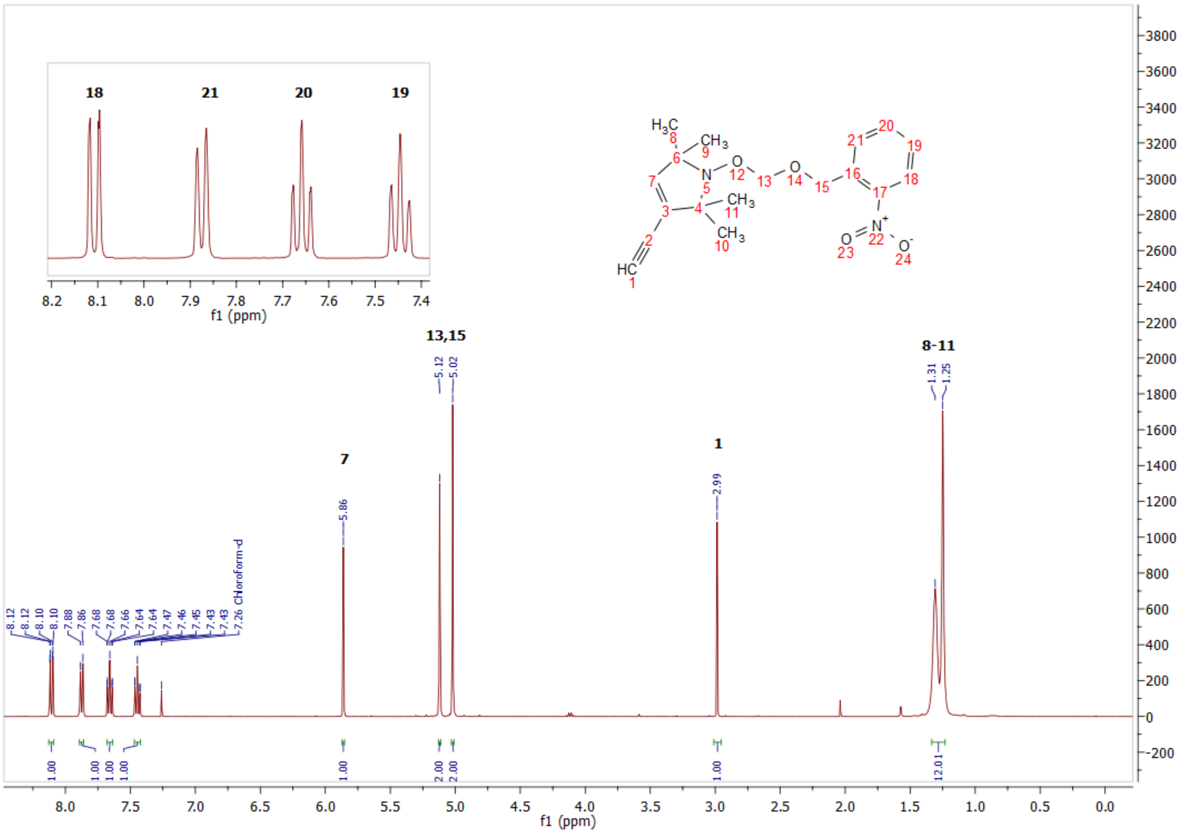

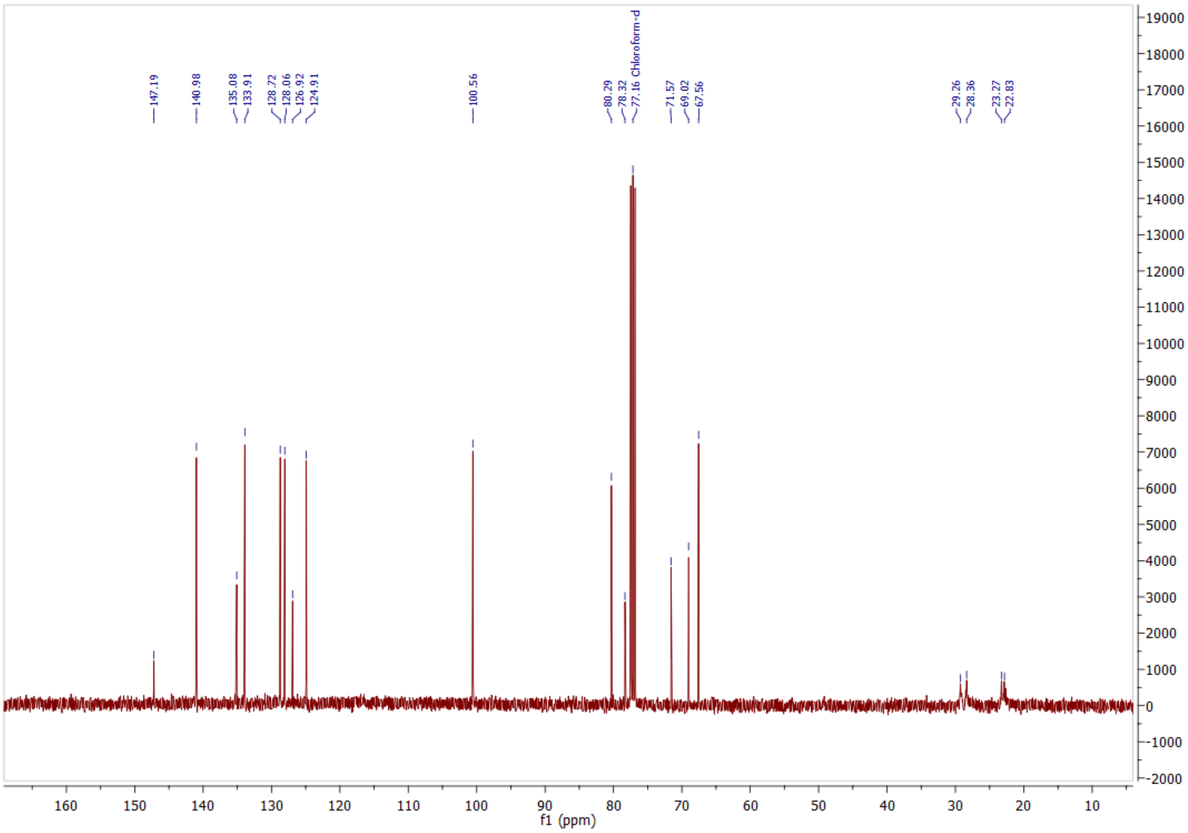

2‘,3‘,5‘-tri-*O*-acetyluridine (**15**)

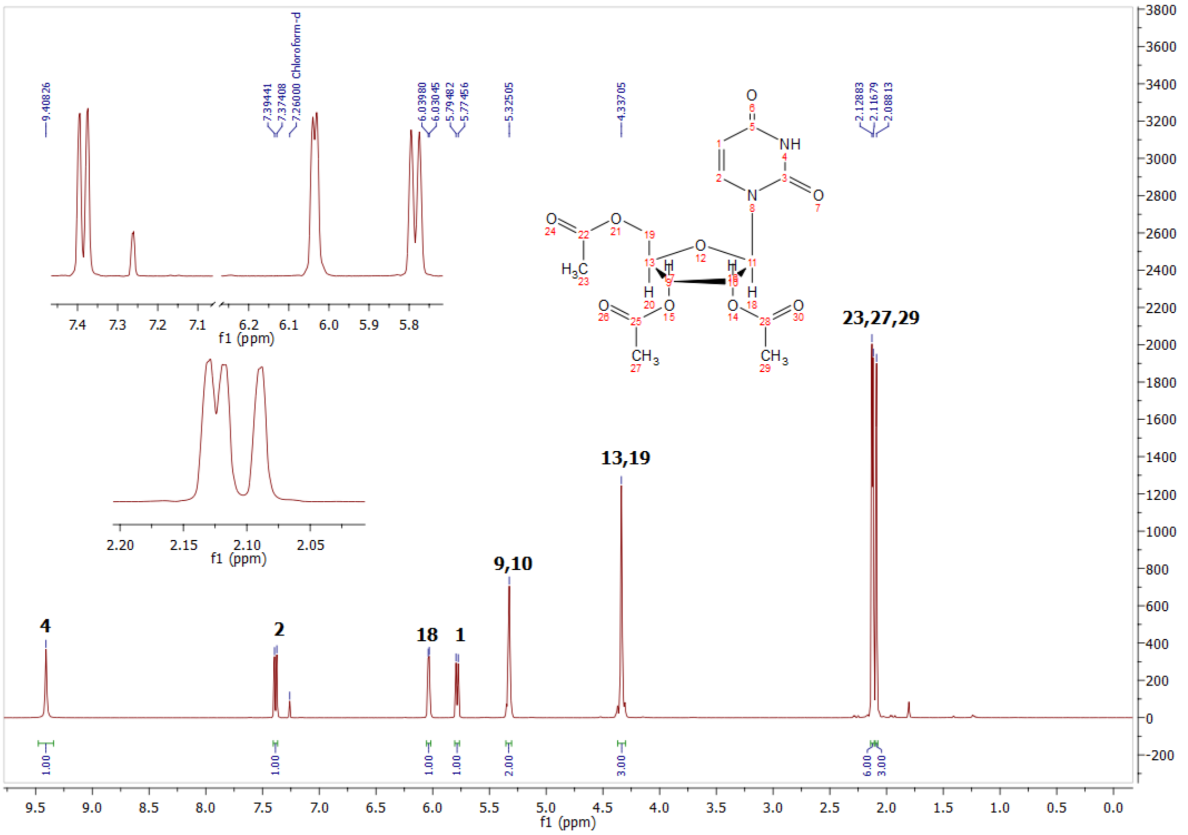

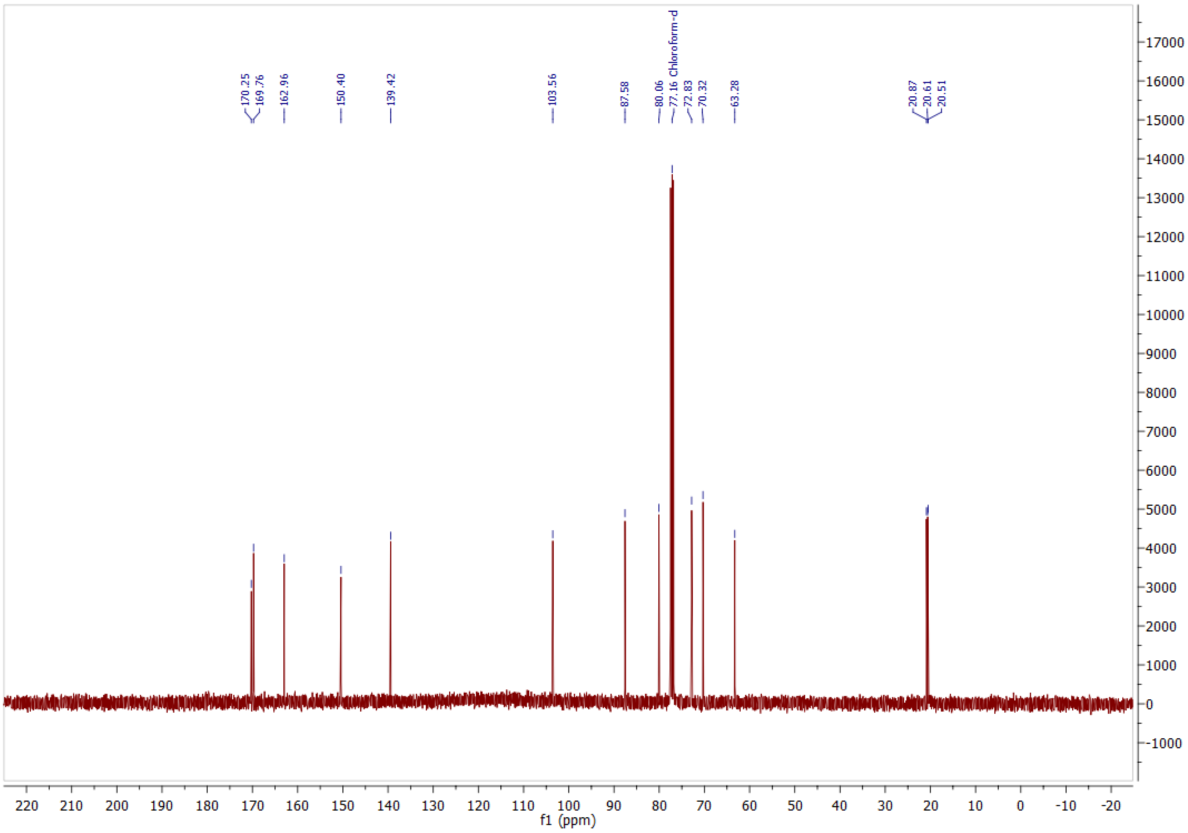

2’,3’,5’-tri-*O*-acetyl-5-iodouridine (**16**)

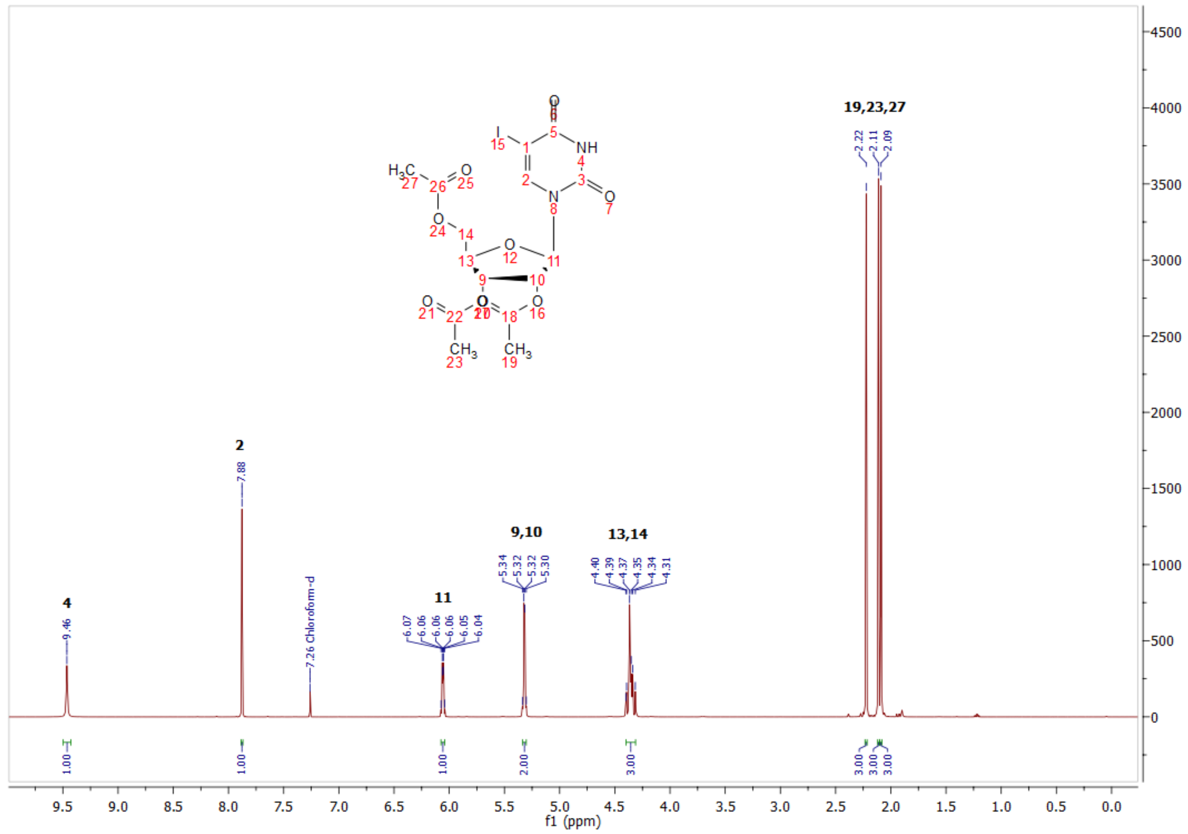

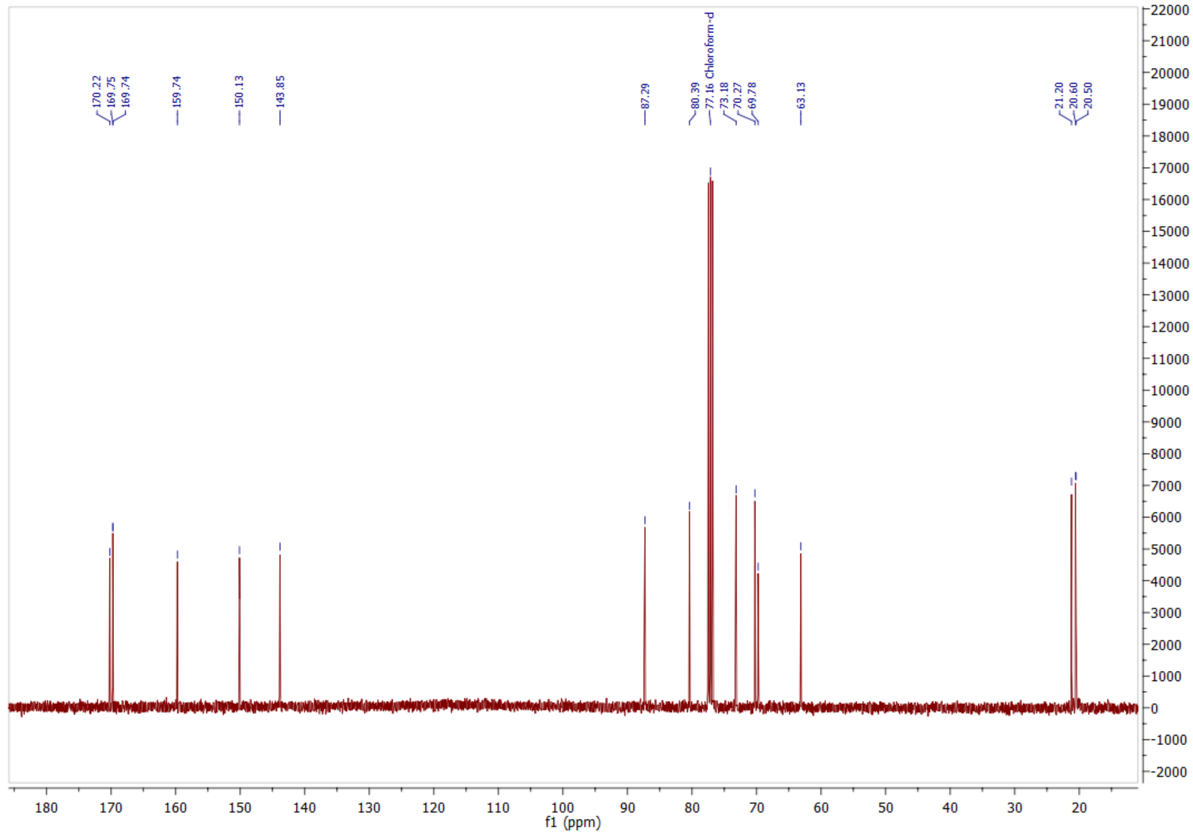

5-iodouridine (**17**)

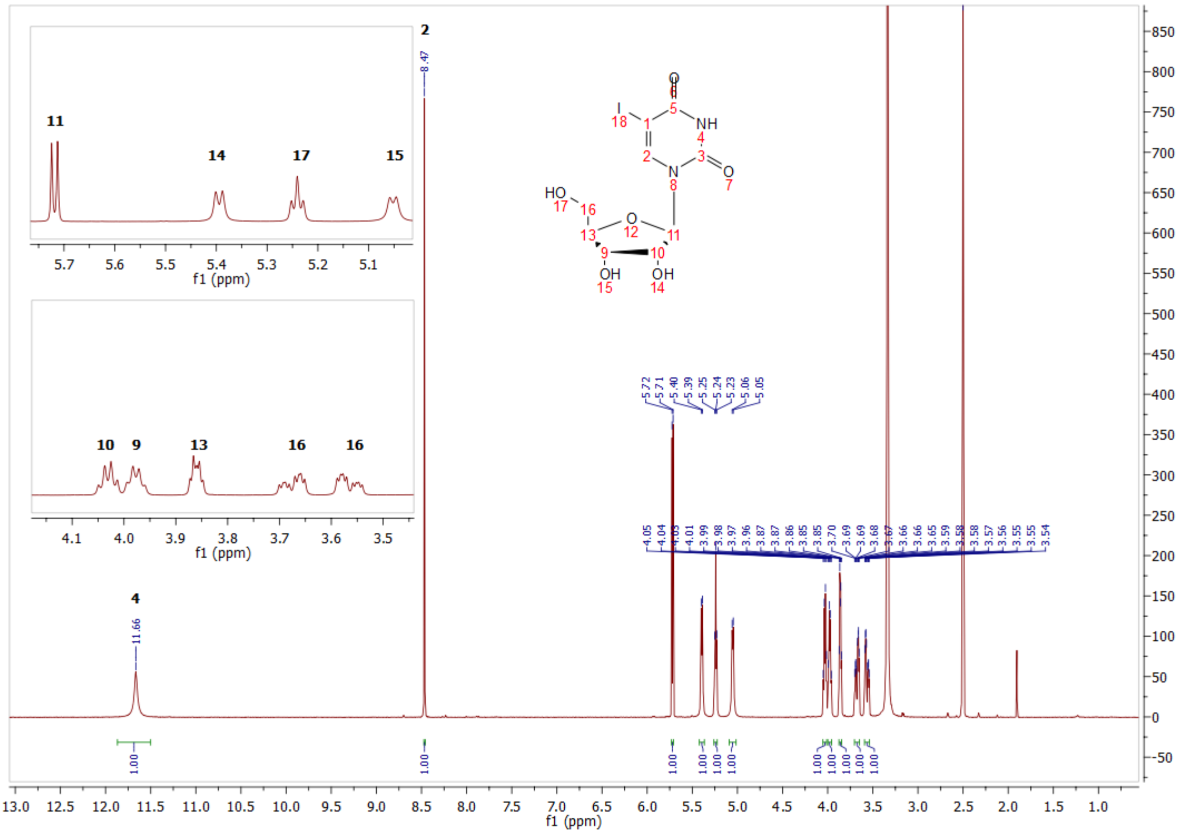

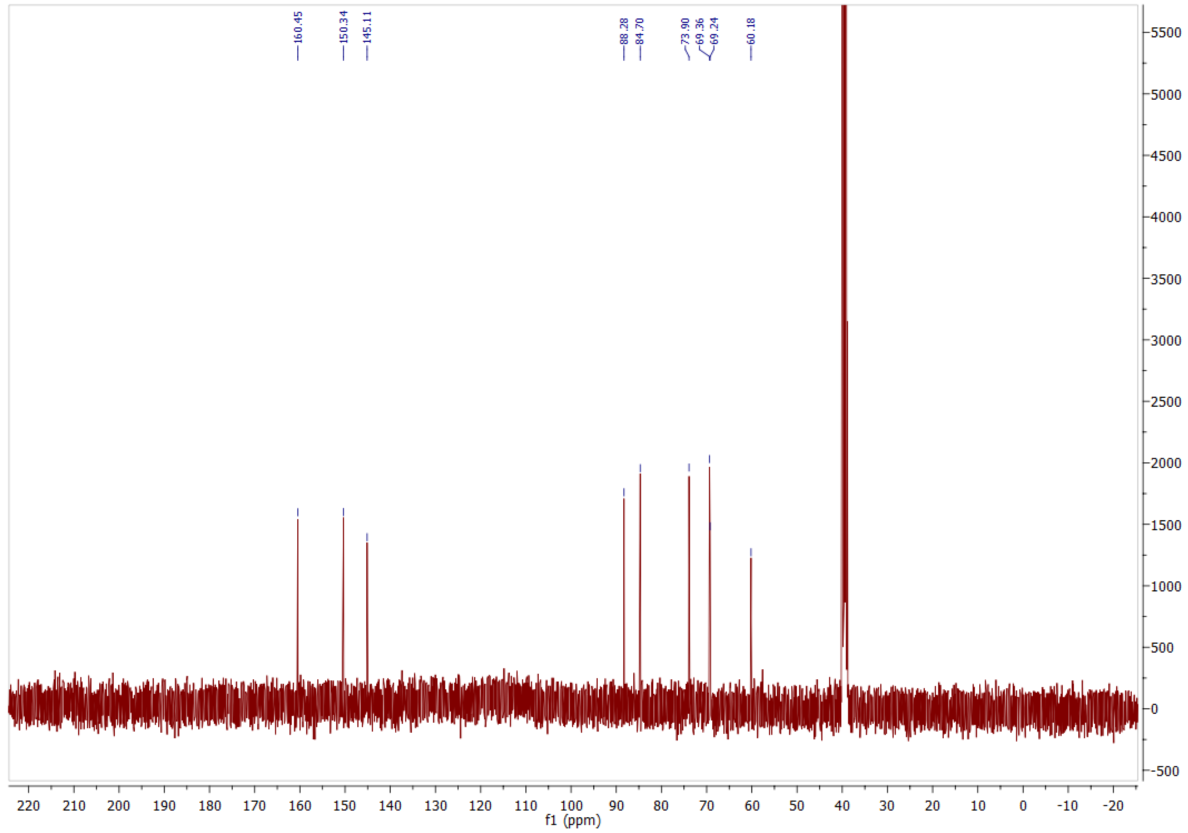

5’-*O*-[bis(4-methoxyphenyl)phenylmethyl]-2’-O-[(1,1-dimethylethyl)dimethylsilyl]-5-iodouridine (**19**)

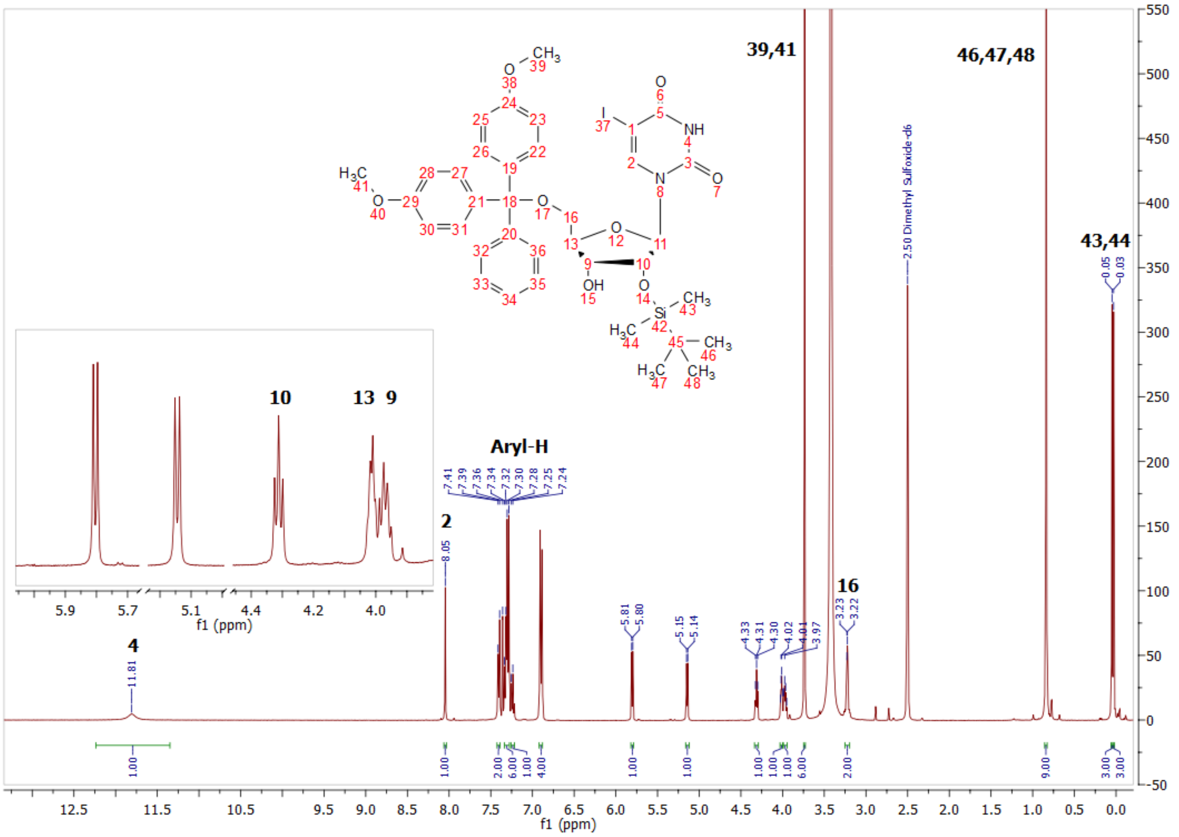

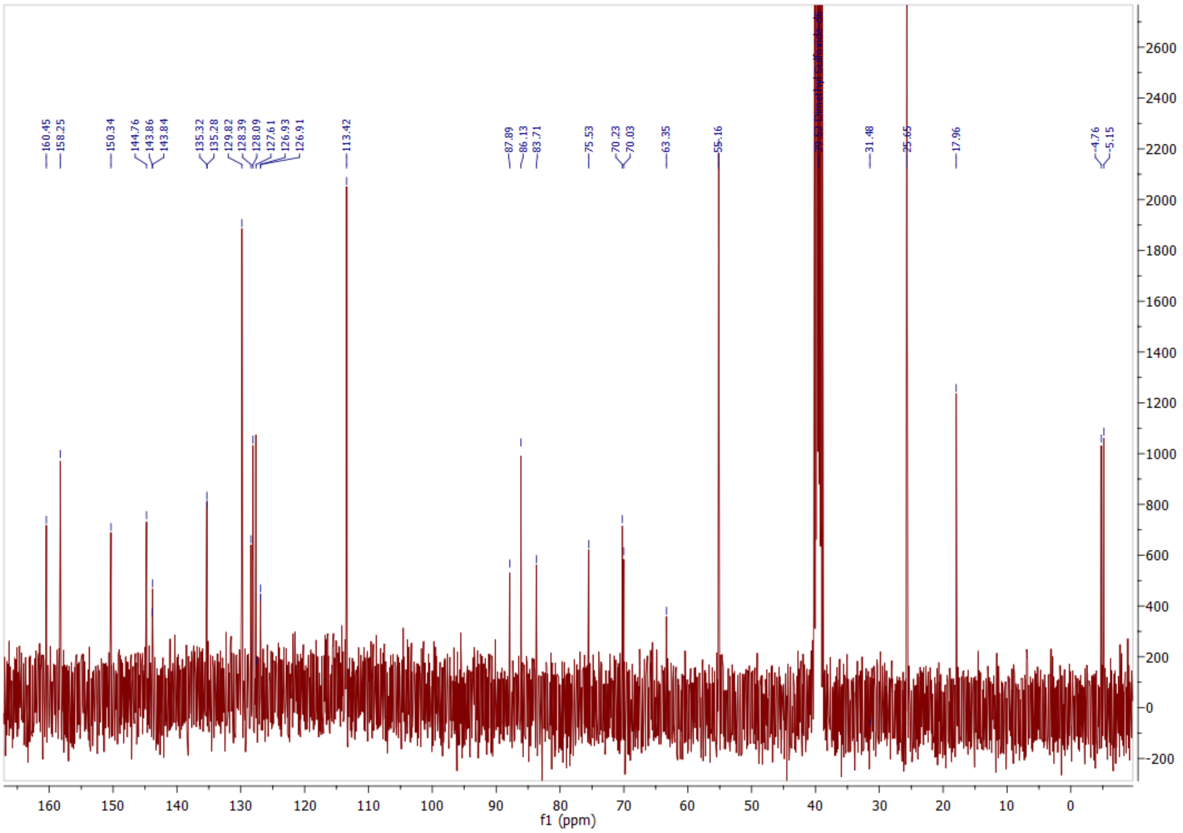

uridine with protected spin label (**20**)

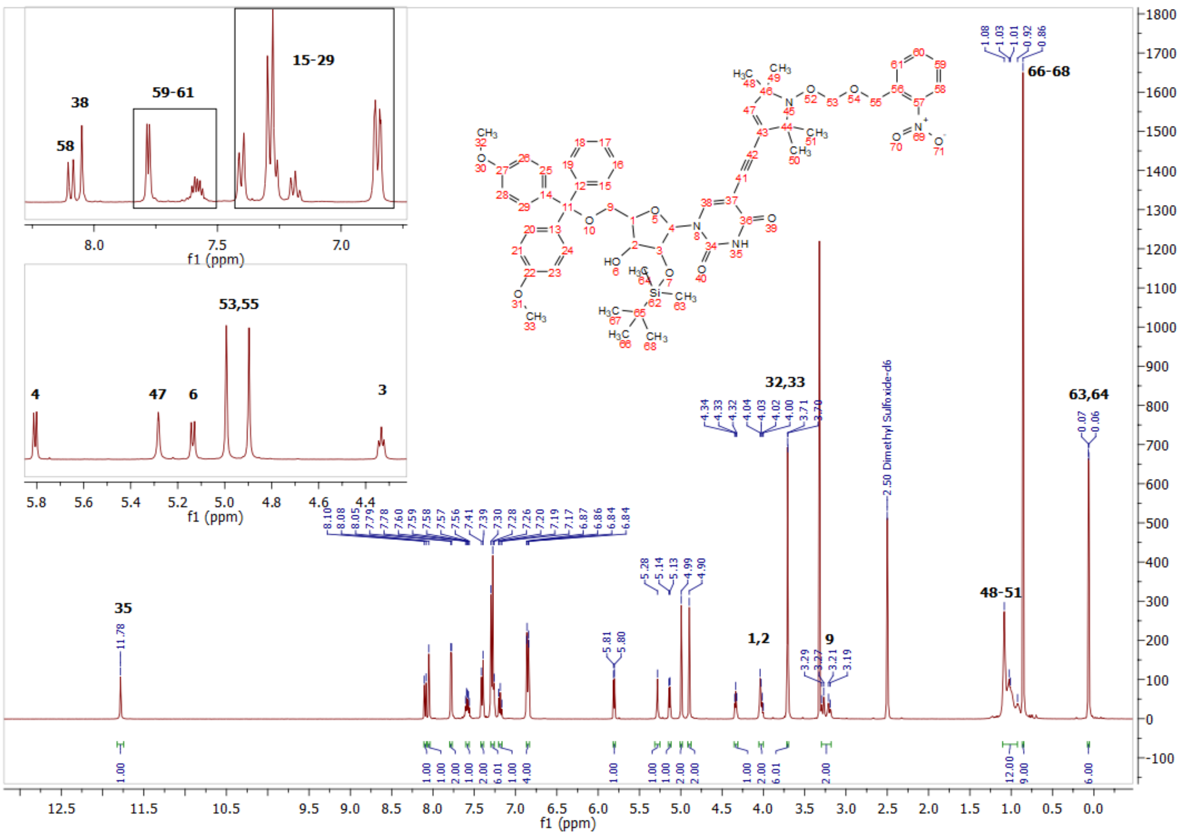

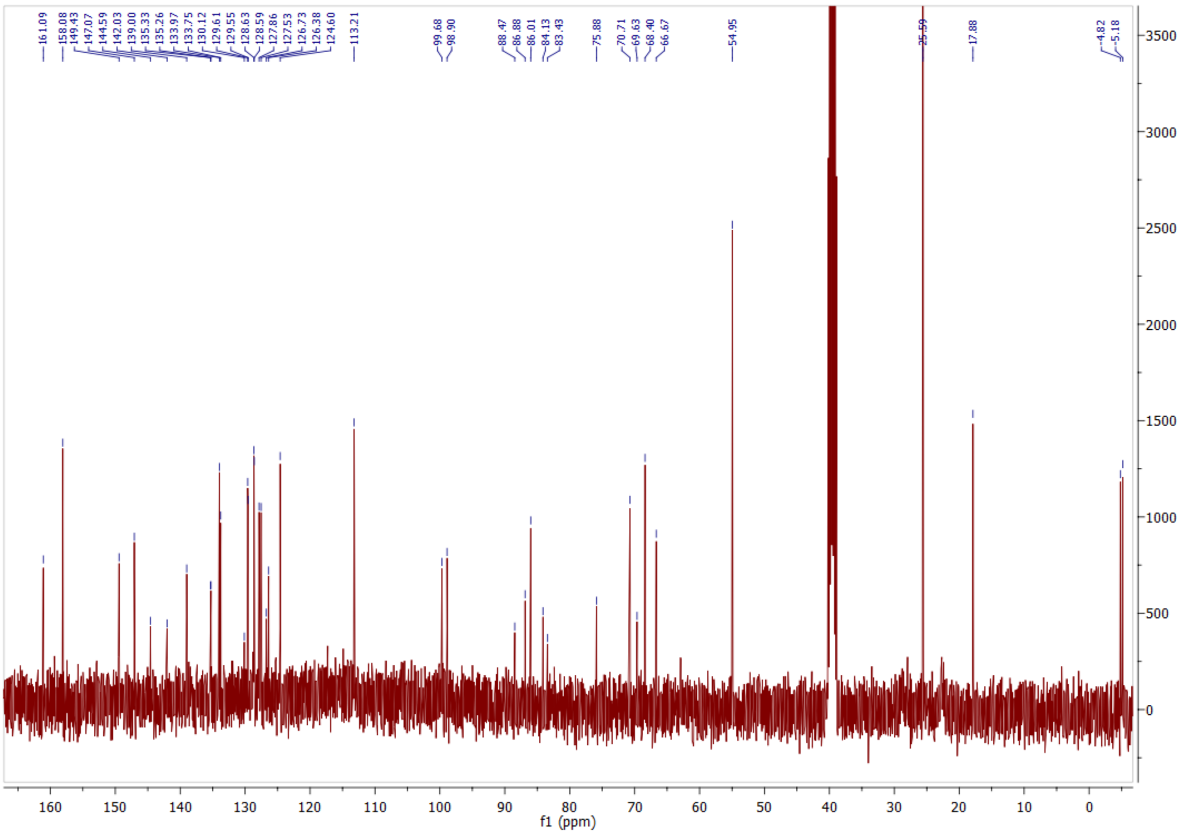

uridine-based phosphor amidite building block (**3**)

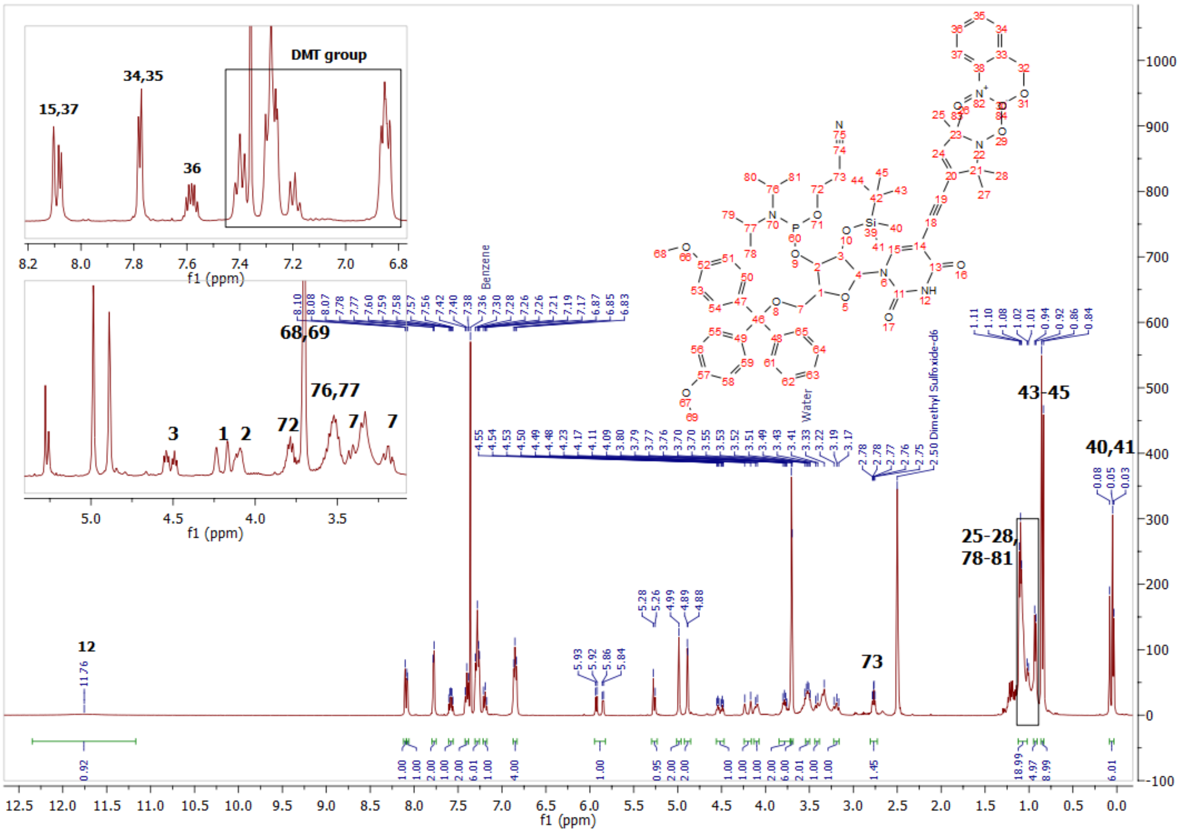

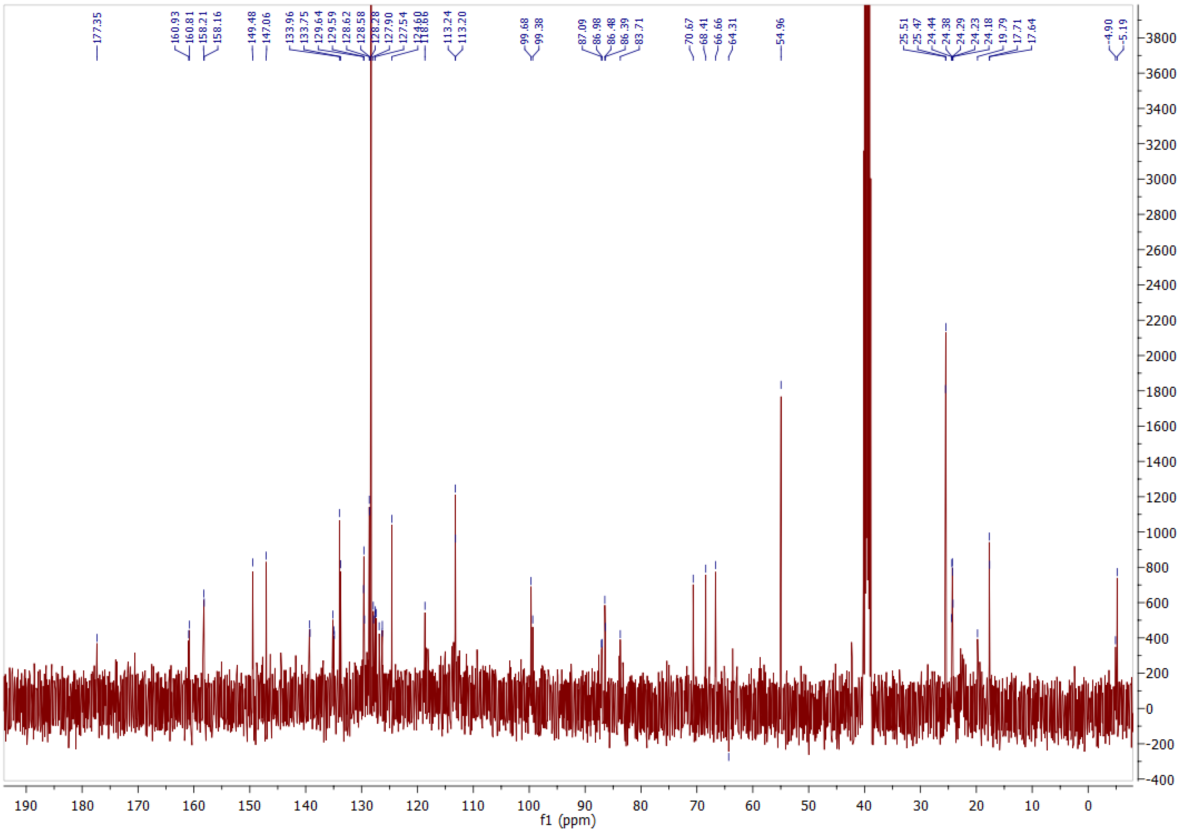

Supplement: Supplementary file 1 — Supporting Information [file CHEM-28-0-s001.pdf]
